# Selecting One of Several Mating Types through Gene Segment Joining and Deletion in *Tetrahymena thermophila*

**DOI:** 10.1371/journal.pbio.1001518

**Published:** 2013-03-26

**Authors:** Marcella D. Cervantes, Eileen P. Hamilton, Jie Xiong, Michael J. Lawson, Dongxia Yuan, Michalis Hadjithomas, Wei Miao, Eduardo Orias

**Affiliations:** 1 Department of Molecular, Cellular and Developmental Biology, University of California Santa Barbara, Santa Barbara, California, United States of America; 2 Key Laboratory of Aquatic Biodiversity and Conservation, Institute of Hydrobiology, Chinese Academy of Sciences, Wuhan, China; 3 Biomolecular Science and Engineering Program, University of California Santa Barbara, Santa Barbara, California, United States of America; 4 The J. Craig Venter Institute, Rockville, Maryland, United States of America; Donald Danforth Plant Science Center, United States of America

## Abstract

The unicellular eukaryote *Tetrahymena thermophila* has seven mating types. Cells can mate only when they recognize cells of a different mating type as non-self. As a ciliate, *Tetrahymena* separates its germline and soma into two nuclei. During growth the somatic nucleus is responsible for all gene transcription while the germline nucleus remains silent. During mating, a new somatic nucleus is differentiated from a germline nucleus and mating type is decided by a stochastic process. We report here that the somatic mating type locus contains a pair of genes arranged head-to-head. Each gene encodes a mating type-specific segment and a transmembrane domain that is shared by all mating types. Somatic gene knockouts showed both genes are required for efficient non-self recognition and successful mating, as assessed by pair formation and progeny production. The germline mating type locus consists of a tandem array of incomplete gene pairs representing each potential mating type. During mating, a complete new gene pair is assembled at the somatic mating type locus; the incomplete genes of one gene pair are completed by joining to gene segments at each end of germline array. All other germline gene pairs are deleted in the process. These programmed DNA rearrangements make this a fascinating system of mating type determination.

## Abstract 2

In *Tetrahymena*, a multi-sexed single-celled organism, the sex of the progeny is randomly determined by site-specific recombination events that assemble one complete gene pair and delete all others.

## Author Summary

*Tetrahymena thermophila* is a single-celled organism with seven sexes. After two cells of different sexes mate, the progeny cells can be of any one of the seven sexes. In this article we show how this sex decision is made. Every cell has two genomes, each contained within a separate nucleus. The germline genome is analogous to that in our ovaries or testes, containing all the genetic information for the sexual progeny; the somatic or working genome controls the operation of the cell (including its sex). We show that the germline genome contains a tandem array of similarly organized but incomplete gene pairs, one for each sex. Sex is chosen after fertilization when a new somatic genome is generated by rearrangement of a copy of the germline genome. One complete sex gene pair is assembled when the cell joins DNA segments at opposite ends of the array to each end of one incomplete gene pair; this gene pair is thus completed and becomes fully functional, while the remaining sex gene pairs are excised and lost. The process involves programmed, site-specific genome rearrangements, and the physically independent rearrangements that occur at opposite ends of the selected gene pair happen with high reliability and precision.

## Introduction

Unicellular eukaryotes reproduce asexually, but most also have a sexual stage to their life cycle that increases genotypic variability. Sexual partners are usually morphologically indistinguishable and mating types, as part of a self/non-self recognition system, foster outbreeding. Mating types were first discovered by Sonneborn in the ciliate *Paramecium aurelia* [1]. This discovery initiated the field of microbial genetics, as mating types were subsequently found in bacteria and a diversity of microbial eukaryotes. The number of mating types and the mechanisms of mating type determination vary widely among unicellular eukaryotes [2]–[6].

*T. thermophila* is a ciliate that segregates germline and somatic functions into two nuclei with distinct genome structures: the diploid micronucleus (germline) and the polyploid macronucleus (somatic). Starvation induces mating (conjugation) between two cells of different mating types. During conjugation (Figure S1) the parental somatic nucleus is destroyed while new somatic and germline nuclei are differentiated from a zygote nucleus. This differentiation includes extensive site-specific genome rearrangements, including fragmentation of the germline chromosomes, de novo telomere addition, and deletion of thousands of internal eliminated sequences (IESs) [7]. Mating type is also determined at this stage [8].

The *T. thermophila* germline *mat* locus was first described by Nanney et al. in 1953 [9] and remains the only locus known to control mating type specificity in this organism. These authors reported that the *mat* locus determines a spectrum of seven mating types (I–VII), one of which is stochastically and irreversibly expressed in each new somatic nucleus. Extensive field collections have revealed no additional mating types [10]. Two classes of germline *mat* alleles are known [10]–[13]. The *mat-1-*like alleles encode mating types I, II, III, V, and VI, while *mat-2*-like alleles encode mating types II, III, IV, V, VI, and VII [9]. All the strains used in this work are homozygous for the *mat-2* allele of inbred strain B. Alternative DNA deletions, rather than epigenetic gene silencing, were proposed to be responsible for mating type determination [14]. The work reported here, made possible by the molecular identification of the mating type genes, has revealed a type of programmed DNA rearrangement in the somatic nucleus that assembles a gene pair of one mating type and deletes the rest.

## Results

### Identification of the *mat* Genes

**Figure 1:**
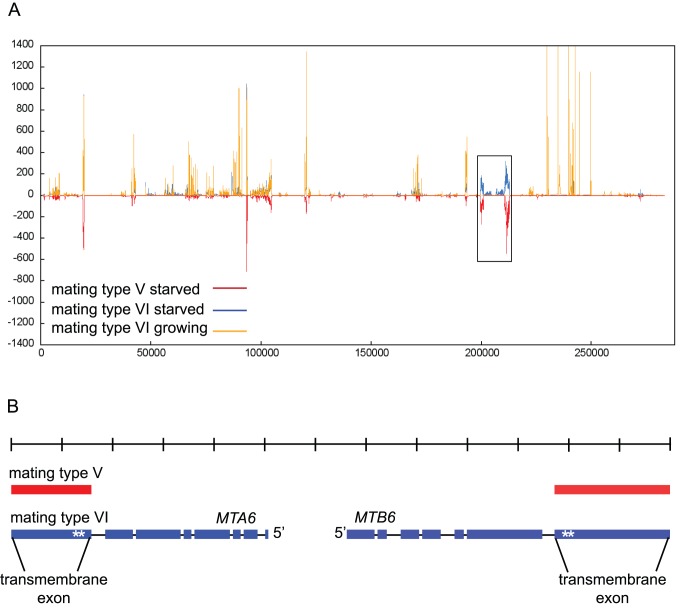
Molecular identification of the mating type locus using RNA-seq. (A) RNA-seq data from mt VI and mt V cells [18] mapped to a ∼300-kb region of the SB210 macronuclear reference genome (mt VI) (see Figure S2). The graph shows the number of RNA-seq reads (*y*-axis) from growing mt VI cells (orange, positive values), 3-h starved mt VI cells (blue, positive values) and 3-h starved mt V cells (red, shown as negative values) that mapped to the ∼300-kb region. Orange overlays blue. The box encloses a segment containing two genes with mating type-specific expression in starved cells and no expression in growing cells. *x*-axis: position within the 300-kb segment. (B) Transcripts (mt VI, blue) and transcript segments (mt V, red) were assembled from RNA-seq reads mapping to the boxed region in (A) and, for mt VI, from sequenced RT-PCR products. 5′ and 3′ untranslated regions are not included. The mt VI-derived transcripts correspond to a pair of divergently transcribed predicted genes (KC405257), now named *MTA6* and *MTB6*, respectively. Thin connecting lines represent introns. Both transcripts are drawn to scale, where each tick mark on the scale represents 1 kb. Each gene contains a TM exon and furin-like repeats (*).

**Figure 2:**
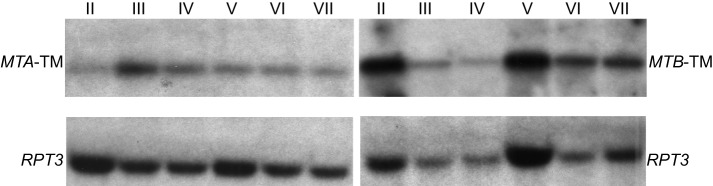
*MTA* (or *MTB*) transcripts share homologous sequence and are of similar size. Whole-cell RNA was extracted from starved mature strains of mating types II through VII (SB4208, SB4211, SB4214, SB4217, SB4220, and SB4223; see Table S1). Probes from within each conserved TM exon were hybridized to Northern blots. The *MTA6*-TM probe hybridized to a ∼5-kb transcript (left panel), while *MTB6*-TM probe hybridized to a ∼6-kb transcript (right panel). *RPT3*, a 26S proteasome subunit P45 family protein (XP_001007748) expressed during starvation, was used as a loading control. The *RPT3* probe hybridized to the expected ∼1.3-kb transcript. The complete blots are shown in Figure S3.

The genetically mapped *mat* locus [15]–[17] was assigned to a roughly 300-kb segment of a somatic chromosome sequence assembly (Figure S2). As cells must be starved to mate, we assumed that a candidate mating type gene would be expressed in a mating type-specific manner during starvation and not expressed during growth. In a previous whole-transcriptome RNA-seq study [18], mRNA was prepared and sequenced from starved SB4217 (mating type V or mt V) cells as well as from starved and growing SB4220 (mt VI) cells (Table S1). To identify mating type candidate genes, we mapped the RNA-seq reads to the 300-kb segment of the mt VI somatic reference genome [15],[19]. Two adjacent genes in this region showed mating type-specific expression in starved cells and no expression during growth (Figure 1A) making them good mating type gene candidates. We named these genes *MTA* and *MTB*. A transcript for each gene was assembled primarily from reads that mapped to the mt VI reference genome. Reads from mt VI covered both genes except for one small gap in *MTA*, which was filled in by cDNA sequencing (unpublished data). Northern blot analysis (Figures 2 and S3) confirmed a single transcript for each mt VI gene. Only the terminal exons of *MTA* and *MTB* could be assembled from the mt V reads that mapped to the mt VI reference genome (Figure 1B). In addition, a partial transcript was assembled de novo from the mt V RNA-seq reads (Text S1). Two thirds of this partial transcript has 99.9% identity with the terminal exon of mt VI *MTA* gene but the remainder is absent from the mt VI somatic reference genome and could encode a mating type-specific segment.

The *MTA* and *MTB* genes identified above are arranged head to head, are divergently transcribed (Figure 1B) and are predicted to code for unique proteins. The *MTA* gene (TTHERM_01087810, KC405257) is predicted to encode a 161-kD protein while the *MTB* gene (TTHERM_01087820, KC405257) is predicted to encode a 194-kD protein. Each terminal exon is unique in the somatic mt VI genome sequence and both are predicted to encode transmembrane (TM) helices. TM domain proteins that can localize to the cell surface could play a role in self/non-self recognition, since cell-cell contact is required to stimulate cells to mate [20]–[22].

### The *MTA* and *MTB* Genes Enable Efficient Mating

If the *MTA* and *MTB* genes determine mating type, they may also be essential for mating. This was addressed by removing the entire somatic gene pair of mt VI (SB210) by homologous gene replacement (Figure S4A and S4B) [23]. The gene pair knockout (MT–) abolished the cell's ability to pair or produce progeny when mixed with starved wild-type (wt) cells of a different mating type or with cells of the same mating type. Identical results were obtained with three independent knockout strains. In contrast, control assays of mating between two wt strains of different mating types showed high levels of pair formation and produced abundant (>85%) progeny.

Each gene of the mt VI gene pair was deleted independently to investigate the functional relationship between the two genes. For both single knockouts, RT-PCR showed that removal of one gene did not abolish expression of the remaining gene (Figure S4C). Three independent *MTB* knockouts (*MTB*–) gave the same results as the gene pair knockout. No progeny were produced when *MTB*– cells were mixed with wt cells of a different mating type. The *MTA* knockout (*MTA*–) retained mating specificity but very little mating competence. It paired extremely poorly and rarely produced progeny (0.16% on average) when mated with wt cells of a different mating type. No pairs or progeny were detected when it was mated to cells of the same (mt VI) mating type. Identical results were obtained with three independent knockout clones.

### The Germline Genome Contains a Tandem Array of Incomplete Mating Type Gene Pairs

To determine whether other mating types express genes containing the TM exons shared by mts V and VI, we isolated RNA from starved, mature strains of each mating type (Table S1). Northern blot analysis revealed that cells of every mating type have *MTA–* and *MTB*-like transcripts (Figures 2 and S3). The length of the transcripts is similar to the lengths of the RNA-seq assembled transcripts, 4.8 kb for *MTA6* (mt VI *MTA*) and 5.7 kb for *MTB6* (mt VI *MTB*). These results, in combination with the RNA-seq results, support the hypothesis that all mating types have *MTA* and *MTB* genes consisting of two segments: one encoding a highly conserved TM segment found in all mating types and the other encoding a larger mating type-specific segment.

**Figure 3:**
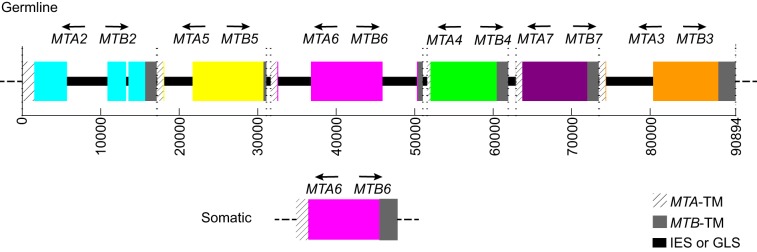
The germline *mat* locus contains six incomplete mating type gene pairs. The locus is a 91-kb tandem array of six incomplete, head-to-head mating type gene pairs, in the order II, V, VI, IV, VII, and III (order established as shown in Figure 4). Each gene pair begins to the left with the *MTA* conserved TM exon (diagonal lines) and ends with the *MTB* conserved TM exon (dark gray). Only the terminal genes (*MTA2* and *MTB3*) have full length versions of their TM exons. The mating type-specific, somatic-destined segment for each mating type gene pair, which includes the 5′ *MTA* and *MTB* segments and the intervening upstream spacer region (putative promoter), is shown as a single thick colored bar. Between the TM exon segments of adjacent gene pairs, there is a small amount of germline-limited sequence (GLS; black). Several IESs are located within the mating type-specific segments (also black). Excluding IES sequence, the mating type-specific segments are of comparable size: II, 8,673 bp; V, 9,132 bp; VI, 9,352 bp; IV, 8,450 bp; VII, 8,277 bp; and III, 8,384 bp. Exact coordinates of all these features are given in Table S4.

To identify the genes of the germline *mat* locus, we used the mt VI *MTA6* and *MTB6* gene pair sequence as query in a BLAST search of the SB210 germline genome sequence (*Tetrahymena* Comparative Sequencing Project, Broad Institute of Harvard and MIT, http://www.broadinstitute.org/). Multiple matching discontiguous segments were observed over a 91-kb region of the germline. The mating type-specific segments of *MTA6* and *MTB6* matched once in the middle of this region. Additional matches were due to the conserved TM exons of *MTA6* and *MTB6*, each of which matched six times within this region. This led us to identify five additional gene pairs containing sequences homologous to those of the TM exons of *MTA6* on the left and *MTB6* on the right. The genes are arranged in a tandem array of six similarly oriented gene pairs, the number of mating types encoded by the *mat-2* allele (Figure 3). Sequence immediately flanking the *mat* locus is identical in the germline and somatic genomes. Before carrying out detailed analysis of the *mat* locus, we filled all sequence gaps in this region and corrected sequence errors (Tables S2 and S3). In the finished sequence we found that each gene pair consists of an *MTA*- and an *MTB*-like gene. These are composed of a unique mating type-specific segment, and a terminal TM exon segment that is highly conserved among the *MTA* (or *MTB*) genes. The germline *mat* locus lacks a complete gene pair. The *mat* locus array begins and ends with the only complete genes within the array, later shown to be *MTA2* and *MTB3*, respectively (Figure 3). The TM exons of all the other mating type genes are truncated, indicated by the use of lower case “tm” (for example, *MTA*-tm or tm). Assembly of a somatic mating type gene pair requires joining of mating type-specific segments to the full-length copies of the *MTA2–* and *MTB3-*TM exons located at the ends of the array.

**Figure 4:**
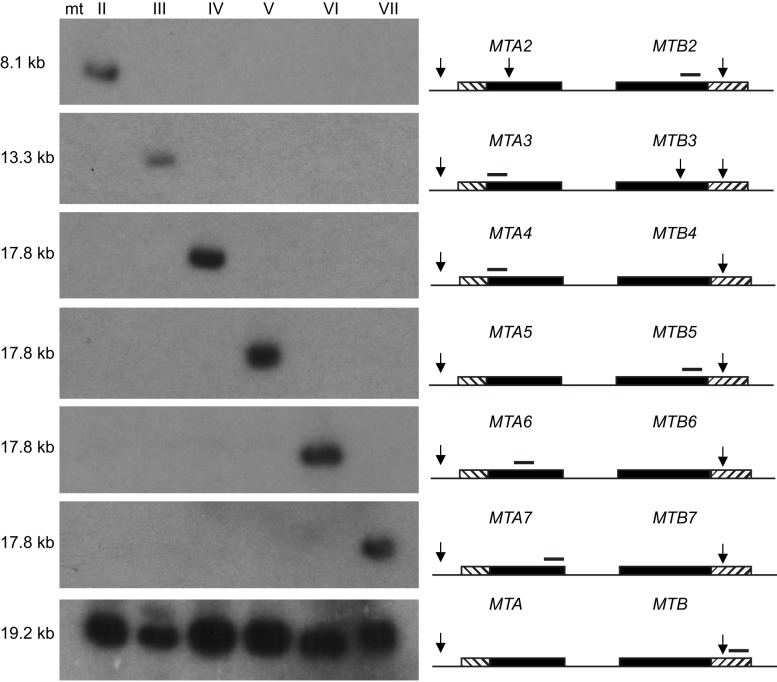
Only one mating type gene pair remains in the somatic nucleus. Southern blot analysis was carried out using whole-cell genomic DNA from a mature strain of each mating type (SB4208, SB4211, SB4214, SB4217, SB4220, and SB4223; see Table S1). The DNA was digested with *PvuII* restriction endonuclease and separated by pulsed-field gel electrophoresis. Black segments, mating type-specific segment of each gene pair; diagonally hatched segments, conserved TM exons; arrows, *PvuII* sites; thin black bars, probes; size (kb) shown is that of the relevant *PvuII* fragment in the somatic genome (the corresponding germline *PvuII* fragments are not visible due to differences in size and copy number).

A mating type was assigned to each germline gene pair segment by Southern blot analysis using probes from unique regions of each germline gene pair. Each probe was found to be mating type-specific, hybridizing to a single band from the somatic nucleus of one mating type (Figure 4). This result clearly shows that only one mating type gene pair remains in the somatic nucleus. The order of the mating type gene pairs in the germline was identified as II – V – VI – IV – VII – III (Figure 3).

Using the above information, the somatic *mat* locus of each mating type was sequenced from mature mating type strains (Tables S1 and S3) derived from a mating between strains SB210 mt VI and SB1969 mt II. The entire germline *mat* locus from SB1969 mt II was sequenced and found to be identical to that of SB210 (Table S3). In the mature mating type strains every somatic gene pair has full-length *MTA–* and *MTB*-TM exons joined to a mating type-specific segment, an arrangement identical to that of the somatic mt VI gene pair. The TM exons of the other mating types revealed several single nucleotide polymorphisms when compared to the mt VI gene pair (see below), but otherwise are identical.

The mating type genes represent two gene families. Predicted proteins within the MTA family are of similar size (1423–1494 aa). Clustal Omega alignment [24],[25] of the six predicted MTA proteins reveals their TM exons share 99.6% amino acid identity (Text S2A). Mating type-specific regions were compared by means of all-by-all pairwise alignments of every MTA mating type-specific amino acid sequence using BLASTP. On average, the alignments covered 98% (range 92%–100%) of the sequences, and showed 42% (range 38%–47%) sequence identity and 60% (range 58%–65%) sequence similarity (identical and conservative substitutions); expected values ranged from 1E-162 to less than 1E-200. Predicted proteins within the MTB family are also of similar size (1,733–1,749 aa). Clustal Omega alignment of the six MTB proteins shows their TM exons share 99.4% amino acid identity (Text S2B). Analogous pairwise alignments of every MTB mating type-specific amino acid sequence on average covered 99% (range 97%–100%) of the sequences, and showed 43% (range 41%–46%) sequence identity and 62% (range 60%–64%) sequence similarity; expected values were all less than 1E-200. The two protein families were compared by all-by-all BLASTP alignments of MTA versus MTB predicted amino acid sequences; in every case, the only significant match (expected value around 1E-08) was restricted to a ∼80 amino-acid cysteine-rich segment containing furin-like repeats, starting about 50 amino acids into the TM exon-encoded sequence. Clustal Omega alignment of the furin-like repeats within the 12 TM exons is shown in Text S3. Cysteines at 12 positions and other amino acids at 14 positions are absolutely conserved among the furin-like repeats of the 12 TM exons. The function of cysteine rich, furin-like repeat domains is not known, but they are found in some endoproteases and cell surface receptors [26].

The mating type-specific segments of the germline gene pairs differ in size by up to 8.5 kb. This variation is due to the presence of IESs, germline-specific sequences that interrupt a contiguous region of somatic-destined sequence, within the array. By comparing somatic sequences to the germline genome sequence, we identified six IESs, (Figure 3; Table S4). Each was confirmed by cloning and sequencing PCR products from the germline and somatic nuclei (unpublished data). The IESs lie within introns in mating type-specific segments or in an intergenic region; they range in size from 299 to 5,989 bp. No other differences were found between the germline and somatic sequences in the mating type-specific segments. Additional germline-limited sequence separates adjacent mating type gene pairs in the germline array (Table S4).

### Conservation of the *MTA*/*MTB* Gene Pair in Other *Tetrahymena* Species

**Figure 5:**
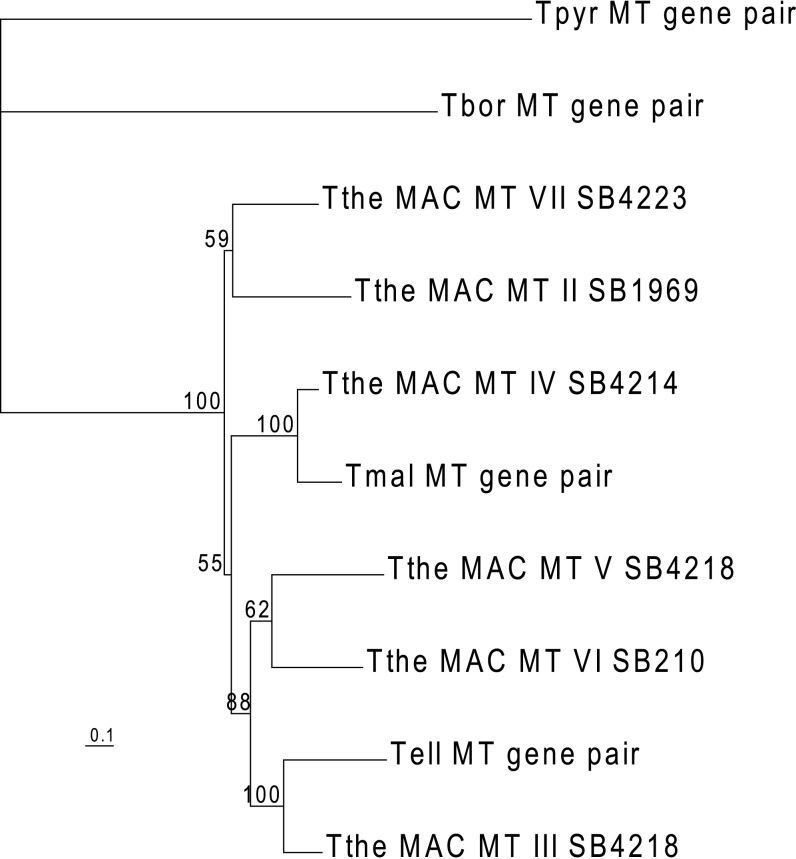
The *MTA* and *MTB* gene pairs are conserved in other *Tetrahymena* species. An unrooted phylogenetic tree of entire mating type gene pairs identified in somatic sequence assemblies of *T. elliotti*, *T. malaccensis*, and *T. borealis* (*Tetrahymena* Comparative Sequencing Project, Broad Institute of Harvard and MIT, http://www.broadinstitute.org/) and *T. pyriformis* strain GL (W. Miao, unpublished data) shows that the sequenced strain of *T. elliotti* can be assigned to mt III and that of *T. malaccensis* to mt IV. The scale bar represents 10% bp substitutions.

We identified homologs of the *MTA* and *MTB* genes in the somatic genome sequence of several additional species (Figure 5). Somatic genome sequence is available for two *Tetrahymena* species that are within the same subgroup [27] as *T. thermophila* (*T. malaccensis* and *T. elliotti*) and two more distantly related species (*T. borealis* and *T. pyriformis*) (*T. malaccensis*, *T. elliotti*, *T. borealis* at the Broad Institute website, *T. pyriformis* strain GL by W. Miao, unpublished data). *T. malaccensis* and *T. borealis* have systems with six and seven mating types, respectively, and like *T. thermophila*, mating type determination is stochastic, without influence of the parental mating types [28]. The mating type system of *T. elliotti* is unknown. The same is true of *T. pyriformis*, where the GL strain is sole representative of this species. This strain also lacks a germline nucleus and thus would be sterile if it could mate. Nucleotide and protein BLASTN and TBLASTN searches using the sequence of the conserved TM exons led us to identify single-copy, head-to-head *MTA* and *MTB* homologs of approximately the same length for all four related species (Text S4). The results of a phylogenetic analysis (Figure 5) and Clustal Omega alignment (Text S5) showed the mating type of the sequenced strain of *T.elliotti* to be most closely related to *T. thermophila* mating type III. Similarly, the mating type of the sequenced strain of *T.malaccensis* is most closely related to mt IV. Alignments of the predicted amino acid sequences are shown in Text S5. For the remaining species, specific mating type relationships could not be recognized either because they carry a homolog of the mt I gene of the *T. thermophila mat1* allele, which has not yet been sequenced, or the sequence divergence is too great. Neither *T. thermophila* MTA nor MTB protein show similarity to any of the other ciliate mating type protein deposited in GenBank, a total of 19 distinct proteins from four *Euplotes* species and one *Blepharisma japonicum* protein, as determined by BLASTP with expected value threshold = 10.

### Complete Somatic Gene Pairs Are Assembled by Joining within the TM Terminal Exons

**Figure 6:**
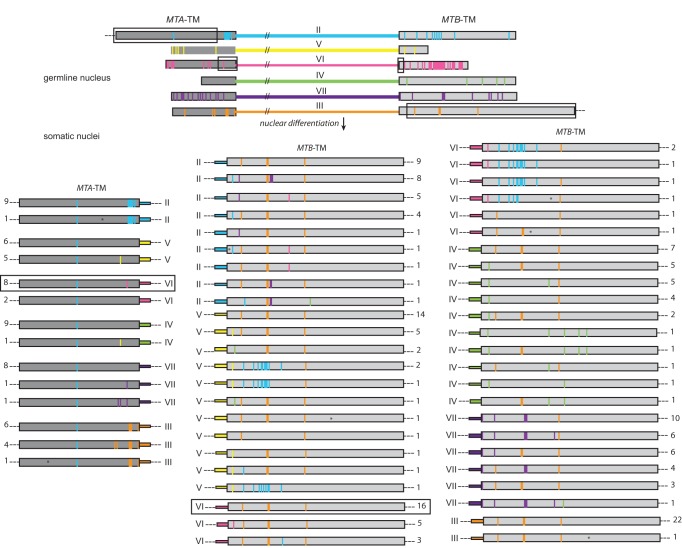
Most assembled somatic TM exons are generated by a single, simple joining event. The sequenced TM exons are from progeny that had not yet undergone their first division (see Figure S1, stage 3, and Materials and Methods). The top six lines represent the germline mating type gene pairs of SB210, shown in their germline order (from top to bottom). All TM exons are drawn to scale. The darker gray bars represent intact and truncated *MTA* TM exons, while the lighter gray bars represent truncated and intact *MTB*-TM exons. The mating type-specific segments are color-coded, as labeled, and are not drawn to scale as indicated by the double slash marks. The dashes beyond *MTA2*-TM and *MTB3*-TM indicate sequence adjacent to the *mat* locus, which is identical in all nuclei. Vertical bars of mating type-specific color within *MTA* and *MTB* TM exon segments represent the location of polymorphic nucleotides relative to the germline consensus sequence of each TM exon (the consensus sequence is shown in Text S6 and a complete list of polymorphisms is shown in Tables S5 and S6). As an example, the simplest possible germline origin of the most common somatic *MTA6*-TM and *MTB6*-TM exons is indicated by boxed regions within the germline mating type gene pairs and somatic exons. For each mating type, approximately ten *MTA*-TM exons and 30 *MTB*-TM exons were sequenced (see Texts S7 and S8 for details). Numbers to the left of *MTA* and to the right of *MTB* TM exons represent the number of times each combination of polymorphic nucleotides was found among the sequenced TM exons. *, location of a base not present in the germline; these changes could be due to either PCR errors or replication repair errors and occurred at a rate of 1 bp in 50 Kbp (see Texts S7 and S8 for details).

The six *MTA* TM exon segments of the germline SB210 *mat* locus were aligned, delineating the position at which each germline tm segment is truncated (Figure 6) and revealing 59 polymorphic sites (Table S5). The *MTB* TM exon segments were similarly examined and 52 polymorphic sites were found (Table S6). With only one exception, none of the polymorphic nucleotides generate stop codons or reading frame shifts and most are unique to a particular gene pair. Unique polymorphic nucleotides within the germline TM exon segments allow us to deduce the germline origin of somatic *MTA*-TM and *MTB*-TM exon DNA.

During differentiation of a new somatic nucleus a pair of intact *MTA* and *MTB* genes must be assembled from the germline genes. One possibility is that joining occurs between the ends of the mating type-specific segment and the start of the *MTA2* and *MTB3* full-length TM exons. If this were the case, all the progeny would have full-length TM exons identical to those of the *MTA2* and *MTB3* germline genes. Alternatively, joining could occur at internal locations within the germline TM exons. In this case, the somatic TM exons would contain novel combinations of the unique polymorphic nucleotides found in the germline tm segments. Somatic mating type gene pair sequences from the mature strains mentioned above, and the SB210 shotgun macronuclear genome sequence, were found to contain novel combinations of these polymorphic nucleotides (see below), suggesting that joining can occur within the germline TM exons.

To determine more precisely where the incomplete gene pairs are joined to full-length germline TM exons, we compared the sequences of TM exons from newly differentiated somatic nuclei to those of germline TM exon segments. We sequenced individual somatic TM exons from progeny that had not yet undergone the first cell division (exconjugants, Figure S1 stage 3). We constructed “collapsed alignments” to concisely represent all the polymorphisms in the somatic and germline nuclei (Texts S6–S8). Schematic representations of the complete set of sequenced exons are shown in Figure 6. Somatic *MTA2* and *MTB3* genes, which are already complete in the germline, showed no evidence of any joining event. The TM exons of every other somatic mating type gene showed polymorphic nucleotide combinations not present in the germline genome (Figure 6; Texts S7 and S8). A single, simple joining event connecting a truncated germline tm segment to the full-length germline *MTA*2-TM exon explains 98% of the somatic *MTA*-TM exons present in early progeny (Table S7A). The *MTA* join sites were mapped to a 269-bp segment near the start of the *MTA*2-TM exon. A single, simple event explains joining to the full-length germline *MTB*3-TM exon in 74% of the sequenced exons (Table S7A). This percentage varies from 42% for somatic *MTB2* to 89% for somatic *MTB6*. The *MTB* join sites mapped to intervals distributed throughout the germline TM exon sequence. The number of distinct join sites may have been exaggerated if PCR template switching [29] reshuffled nucleotide diversity in these sequenced TM exons (Text S9). These data confirm that many if not all of the joining events occur within the TM exon rather than exclusively between the TM exon and the mating type-specific segment. The frequency of novel nucleotides (not present in the germline) is less than one in 50,000 sequenced base pairs (Figure 6 legend), showing that the joining events are highly precise.

Analysis of the TM exons of twenty 120-fission strains (Table S7; Texts S10 and S11) shows that *MTB*-TM exons undergo additional recombination after the resumption of vegetative multiplication. Highly significant differences between 0- and 120-fission cells are observed for the *MTB*-TM exons, whether one compares the number of haplotypes explained by a single joining event, or by recombination events involving more than two germline genes, or by gene conversions (Table S7). PCR template switching is excluded as a spurious source of recombination in these results (Text S9). We believe these events largely represent intragenic secondary recombination, distinct from the single, simple recombination events responsible for mating type determination.

## Discussion

Our findings suggest that mating type determination in *T. thermophila* involves a remarkable type of programmed genome rearrangement. We have identified a pair of mating type genes that are arranged head-to-head. Each mating type is characterized by a similarly organized pair of somatic genes and each gene of the pair encodes a TM domain shared by all mating types. Starvation is required for mating and induces transcription of both genes. Both genes are required for wt levels of pair formation and progeny production. The germline genome contains an array of incomplete gene pairs, one for each mating type. During development of the somatic nucleus in progeny cells, the germline array undergoes rearrangement to assemble one complete gene pair and delete all others in the somatic chromosome. Thus, mating type determination occurs by deletion rather than by an epigenetic gene silencing mechanism. These findings account for the irreversibility of mating type determination. The mating type locus can be thought of as a multi-state developmental switch where the switch is stochastically and permanently set to one state in the somatic genome.

The removal of either or both genes caused a significant inhibition of pairing between cells of different mating types, suggesting the *MTA* and *MTB* genes are both fundamental for recognition of cells of a different mating type (allorecognition). This inhibition of pairing suggests that the gene products may be functioning cooperatively for allorecognition. In addition to allorecognition, the gene products could be distinguishing self to prevent homotypic pairing. If this were the case, homotypic pairing would be observed in the absence of one or both genes. This does not appear to be a function of the *MTA* and *MTB* genes because pairing between starved cells of the same mating type was not observed in our knockouts.

**Figure 7:**
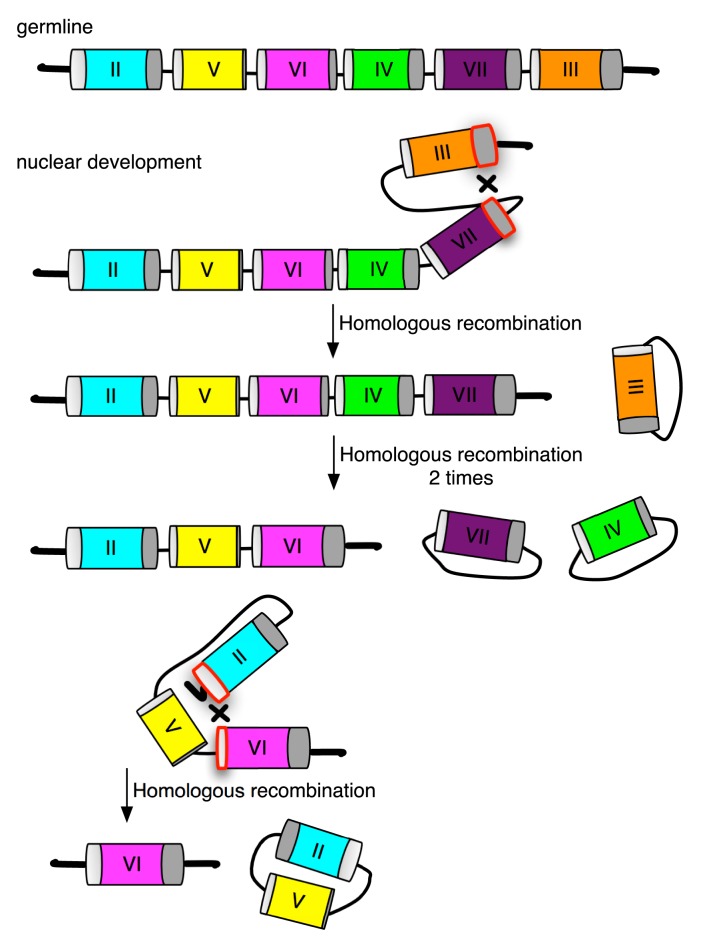
Model proposing that homologous recombination assembles a single mating type gene pair during somatic differentiation. In this model, intramolecular recombination events are initiated at both ends of the germline array; subsequent resolution results in removal of intervening gene pairs by looping out and joining of a gene pair to the full length TM exons at the ends of the array. Any number of gene pairs could be excised in a single recombination event; since the chromosomal product regenerates the recombination substrate, recombination steps can be reiterated until a single, complete gene pair remains, at which point the process has to stop. Sequestering or disabling the ability of side products to recombine again would minimize unproductive reversal of the process. Recombination events need not always involve a full length TM exon; two internal tm exons could also be involved at intermediate steps. The recombination process is labeled “homologous recombination” for simplicity, but identical results could be obtained by highly precise non-homologous end-joining. Side products containing a discrete number of gene pairs, shown here as circular, could also be linear depending on the details of the recombination and repair mechanism. A related DNA rearrangement model of *T. thermophila* mating type determination, also involving recombination and alternative deletion in a tandem array of germline mating type genes was proposed previously [14]. The key conceptual difference is that in the original model a unique segment was somatically attached at one end of an individual mating type gene, instead of attaching unique segments at both ends of a mating type gene pair, as reported here.

At least two events are required to assemble a complete somatic mating type gene pair from the *mat* germline array (see model shown in Figure 7). At the left end of the gene pair, the *MTA*-tm segment must be joined to the single copy, full-length *MTA*2-TM exon located at the far left end of the array. At the right end of the same gene pair, the *MTB*-tm segment must join to the single copy, full-length *MTB*3-TM exon located at the far right end of the array. The breakage and rejoining mechanism is highly precise. Since both joining events occur within translated exons segments, without this precision mating competence could be lost. Possible mechanisms include homologous recombination and precise nonhomologous end joining. The mechanism will become clearer once we experimentally determine which of the observed recombination events are essential to mating type determination and which are unrelated to this process. Regardless of the mechanism, an interesting question is how joining at opposite ends is coordinated to result in the assembly of a somatic gene pair. A stochastically selected germline gene pair may be epigenetically marked, its two ends cut, and full length TM exons joined coordinately. Alternatively, each end could be processed independently resulting in the deletion of one or more gene pairs from either end, until only one complete gene pair remains. Additional knowledge of the mechanism will be needed to understand how mating type frequencies are influenced by environmental conditions, such as temperature and nutritional state [30],[31].

In addition to the single, simple recombination events associated with mating type determination, we have observed secondary recombination events in somatic TM exons, especially *MTB* TM exons. These events are particularly frequent in the *MTB* TM exons of mature cell lines (Table S7). As explained in Text S9, artifacts of PCR template-switching are excluded in these results. Since the majority of joined TM exons from 24-h exconjugants show no evidence of secondary recombination, these events are probably unrelated to mating type determination. Presumably they chiefly reflect recombination between multiple somatic chromosome copies carrying independently differentiated TM exons prior to the purification brought about by assortment during vegetative multiplication (Figure S1). A number of recombination events, most simply interpreted as gene conversions, have also been detected among *MTB* exon haplotypes. We believe that these *MTB* gene conversions are also due to the secondary recombination described above and are unrelated to *Tetrahymena* mating type determination, in part because gene conversions are found in only a small minority of the sequenced TM exons in 24-h exconjugants. In addition, gene conversion per se cannot result in the loss of intervening mating type gene pairs. Gene conversion is responsible for mating type switching in yeast, but no intervening DNA is lost in yeast mating type switching [32].

Programmed somatic DNA rearrangements are well known among the ciliates [33],[34]. In *T. thermophila*, approximately 6,000 IESs in the germline genome are excised during differentiation of a new somatic nucleus [35]. The deletions that join TM exons to mating type-specific segments differ in several important ways. IES excision is imprecise; precision is not required, as nearly all IES are found in intergenic regions or within introns [36]. In contrast, the deletions involved in mating type determination are highly precise and occur within the coding segment of the TM exon. Furthermore, IES excision is maternally controlled; only sequences absent from the parental somatic genome are targeted for elimination [37],[38]. Mating type, on the other hand, is stochastically inherited; determination of mating type in each progeny cell occurs autonomously during the differentiation of the new somatic nucleus. Mating type-specific sequences absent from the parental somatic nucleus escape deletion by the IES excision mechanism and are retained in progeny somatic nuclei. Finally, preliminary experiments (unpublished data) indicate that mating type determination occurs several hours after excision of IES within the *mat* locus. All these considerations lead us to conclude that these two processes, which occur in the differentiating somatic nucleus, proceed by different mechanisms. In mating type determination, DNA breakage and rejoining occurs physically independently and precisely at both ends of one gene pair. This leads to the assembly of one complete gene pair and the excision of the other germline gene pairs from the somatic chromosome. To our knowledge, this type of programmed genome rearrangement is novel, at least in ciliate molecular biology.

The modular organization of the *T. thermophila* germline *mat* locus (Figure 3) in combination with rare unequal meiotic crossing-over between homologous *germline* TM/tm domains could facilitate rapid evolutionary change in the number of available mating types. This hypothesis is consistent with the existence of two *T. thermophila* germline *mat* allele classes specifying different numbers of mating types (five for *mat-1* and six for *mat-2*). *mat-1*-like alleles carry mt I but are missing mts IV and VII. *mat-2*-like alleles are the opposite, carrying mts IV and VII in adjoining gene pairs while missing mt I. Using somatic genome sequence data we assigned a mating type to the sequenced strains of two other *Tetrahymena* species by virtue of their similarities to *T. thermophila* mating types. This suggests that a similar mating type system is conserved in multiple *Tetrahymena* species. If so, the mechanism proposed above could also explain the finding that the number of mating types described in species of the genus *Tetrahymena* is dynamic, ranging from 3 to 9 (reviewed in [39]). Using the strong sequence conservation observed at the TM exons, it may be possible to isolate and sequence mating type genes from many species of the genus *Tetrahymena* to investigate the evolution of their mating type system.

*T. thermophila* is a model organism for eukaryotic biology [15]. Future research of this mating type system should advance our knowledge in several areas of biology. The biochemical functions of the *MTA* and *MTB* gene products are of interest for understanding the principle of self/non-self discrimination. The study of genomic rearrangements employed for mating type determination can inform mechanisms of genome dynamics in other systems.

## Materials and Methods

### Strains

All of the *T. thermophila* strains used here have the inbred strain B genetic background [40]. As such, they are *mat2*/*mat2* homozygotes and can be of any one of mating types II–VII. The somatic and germline genomes have been sequenced from strain SB210 [41]. A panel of mature strains of different mating types, F1s of a cross of SB210×SB1969, was obtained by propagating F1 cells for ∼120 fissions, subcloning and determining their mating type, all using established methods [42]. The germline *mat* locus alleles of SB210 and SB1969 are identical to the nucleotide, as determined by sequencing SB1969 (Table S3). This identity should extend to the germline of all members of the F1 panel. Strains are listed in Table S1 and are available through the National Tetrahymena Stock Center (http://tetrahymena.vet.cornell.edu).

### RNA-Seq to Identify Candidate Genes

An RNA-seq-based whole transcriptome analysis was done using strains SB4217 (mt V) and SB4220 (mt VI); detailed information of RNA extraction, library construction, and deep RNA sequencing can be found in our previous work [18]. For the work here, we compared three conditions: starved mating type VI cells, growing mating type VI cells, and starved mating type V cells. To look for transcription differences between mating type V (SB4217) and mating type VI (SB4220), RNA-seq reads were first mapped to the ∼300-kb region of SB210 (mt VI) that includes the *mat* locus (Figure S2) using TopHat [43]. Mapped reads were assembled as transcript fragments using Cufflinks [44]; the command lines of this mapping-then-assembly pipeline were described previously [18]. Gbrowse genome viewer (http://gmod.org/wiki/GBrowse) was setup to visually check the transcription differences using as input the TopHat mapping results and the Cufflinks assemblies. Necessary data format transformations were performed using SAMTools [45] and ad hoc Perl scripts. The ∼300-kb region was then manually examined in Gbrowse for significant mating type-specific transcription differences.

We de novo assembled a mating type V partial transcript using the Trinity transcriptome assembler (2011-08-20 release version) [46]. The command line used was: Trinity.pl –seqType fq –output output –left left.fq –right right.fq –run_butterfly –bflyHeapSpace 20000M. To identify de novo assembled mating type V partial transcripts related to the mating type locus, similarity searches (BLASTN) were performed against the de novo transcript assemblies using as query the sequences of genes TTHERM_ 01087810 and TTHERM_ 01087820. All identified mating type V transcripts were aligned with mating type VI transcripts of the two genes to discern conserved and mating type-specific sequences.

### PCR, Cloning, and Sequencing

Table S2 lists primers used to PCR amplify gaps in the SB210 germline sequence in the region of the *mat* locus, the *mat* locus from the SB1969 germline sequence, regions flanking IESs in the somatic genome, segments for knockout constructs, and the TM exons from the somatic nuclei of mature strains. DNA was prepared as described [47]. PCR products were amplified with Finnzymes Phusion High-Fidelity DNA Polymerase and cloned using the Zero Blunt TOPO PCR Cloning Kit for Sequencing (Invitrogen). Primers for sequencing were chosen using the SB210 genome sequence. Sequencing by Sanger dideoxy sequencing was carried out at Eton Bioscience Inc.

### Northern Blots

RNA was extracted using the Qiagen RNeasy kit from 10-ml cultures at 2×10^5^ cells/ml that had been starved for 3 h at 30°C in 10 mM Tris (pH 7.4). 15 µg of RNA was loaded per lane on a 1% gel, subject to electrophoresis for ∼2 h at 120 V in formaldehyde buffer, and set up for downward transfer in denaturation buffer to a charged nylon membrane. To prepare hybridization probe, 150 ng of PCR product was labeled with dATP^32^P by random primer labeling for 72 h at room temperature, followed by removal of unincorporated dATP^32^P nucleotides using the QIAquick nucleotide removal Kit (Qiagen). Pre-hybridization and hybridization with ULTRAhyb solution (Ambion) was at 45°C for 2 and ∼16 h, respectively. Blots were washed twice for 10 min in 0.1× SSC/0.1% SDS at 45°C and hybridization was visualized on film.

### Construction of *mat* Knockout Strains

The *MTA* and *MTB* genes in the somatic nucleus of mating type VI were replaced, separately or together, by a neo-cassette that confers Cd-inducible resistance to paromomycin [23]. Each construct contains the neo cassette, flanked by a minimum of 500 bp of sequence from each side of the coding sequence to be replaced. Replacement occurs by precise homologous recombination. In constructing the single knockouts, 8 bp of the spacer region before the start codon of *MTA6* and 67 bp before the start codon of *MTB* were removed in addition to the coding sequence. For the double knockout both coding sequences and the spacer region were replaced. Constructs were created by overlapping PCR [48].

Biolistic transformation was carried out as described [49]. To select for complete phenotypic assortment to the KO allele, transformants were initially selected in 0.1 µg/ml CdCl_2_ and 100 µg/ml paromomycin and were propagated for more than ∼100 successive cell divisions in the presence of increasing amounts of CdCl_2_ and paromomycin (final concentrations 0.2 µg/ml CdCl_2_ and 1 mg/ml paromomycin). These transformants were then screened by PCR using Choice-Taq DNA Polymerase (Denville Scientific Inc.) and IES-bracketing primers to verify that they had no remaining copies of the wt gene pair in their somatic nucleus (see Table S2 for primers).

### RT-PCR

RNA from the *MTA* knockout strains, *MTB* knockout strains, SB210, SB4208, SB4211, SB4214, SB4217, and SB4223 was prepared as for the Northern blots, and used in cDNA synthesis with the ThermoScript RT-PCR System (Invitrogen). Subsequent PCR was done with primers in Table S2.

### Testing *mat* Knockout Strains for Ability to Produce Sexual Progeny

Somatic *mat* knockout strains are derived from SB210 (mt VI) and thus are homozygous for 2-deoxygalactose-resistance in their germline, but have only the wt, 2-deoxygalactose-sensitive, allele in their somatic genome (Table S1). The fully assorted *mat* knockout strains were crossed to SB1969, a mating type II strain that is homozygous for cycloheximide-resistance in its germline, but has only the wt, cycloheximide-sensitive, allele in its expressed somatic genome. As a control, SB1969 was mated to SB210. Established methods for mating *Tetrahymena* strains and progeny selection were used [42]. The mixtures were allowed to mate for 24 h in starvation medium and upon re-feeding they were diluted and distributed to 96-well plates at 5, 50, 500, or 5,000 cells per well (50 µl per well). Progeny were serially selected for resistance to cycloheximide and 2-deoxygalactose to verify the expected double-resistant phenotype and to quantitate the fraction of cells producing progeny.

### Pulsed Field Electrophoresis and Southern Blots

Cells (∼8×10^5^ cells per plug) of mature strains of mating types II through VII (SB4208, SB4211, SB4214, SB4217, SB4220, SB4223) were washed in 10 mM Tris (pH 8.0), resuspended to 50 µl per plug, mixed with 1.5% low melting point agarose (SeaPlaque GTG), and distributed into plug molds (Bio-Rad). Plugs were incubated overnight at 55°C in NDS/proteinase K (1% NDS, 10 mM Tris [pH 7.6], 0.5 M EDTA, proteinase K 100 µg/ml), washed 2× for 2 h in 0.5 M EDTA at room temperature and stored at 4°C in 0.5 M EDTA. Plugs were then washed 2×10 min in TE at 4°C and digested with *PvuII* restriction endonuclease. A 1% gel (Pulsed Field Certified Agarose, Bio-Rad) was poured around plugs adhered to the teeth of a gel comb, and subjected to pulsed-field gel electrophoresis in CHEF-DR III Pulsed Field Electrophoresis System (0.5× TBE, ramping switch time of 0.5 to 3 s, 6 V/cm, angle 120° for 15 h). The gel was transferred under alkali conditions to positively charged nylon membrane following the protocol in CHEF-DR III Pulsed Field Electrophoresis Systems Instruction Manual, BioRad. Primers for the mating type-specific PCR products used as probes are listed in Table S2. Probes were prepared as for Northern blot analysis above. Pre-hybridization at 45°C for 2 h and hybridization at 60°C for 16 h were done in hybridization solution (6× SSC, 5× Denhardt's solution, 20 mM Tris-HCl [pH 8.0], 0.1% sodium dodecyl sulfate, 2 mM EDTA, and 27 µg/ml denatured salmon sperm DNA). Blots were washed once in 1× SSC/0.1% SDS at room temperature for 10 min, and twice in 0.1× SSC/0.1% SDS at 55°C for 10 min. Hybridization was visualized on film.

### Structural Annotation of the *mat* Genes

Structural annotation of *mat* genes was performed on genomic DNA sequences derived from the somatic genomes of each of six mature strains [SB1969, SB4213, SB4214, SB4218, SB210, and SB4223] expressing different mating types. The strategy was developed as part of an ongoing reannotation of the *T. thermophila* macronuclear and micronuclear genomes. The gene-finding algorithms AUGUSTUS [50] and GeneZilla [51] were re-trained for *T. thermophila* using a set of full-length RNA-seq transcripts and used to perform gene predictions. Additionally, we ran the AAT tool [52] against a JCVI in-house non-redundant protein database (AllGroup) to map known proteins to the loci and PASA [53] to map transcripts assembled from RNA-seq data [18] using Trinity [46]. Using this evidence, each gene, including 500 bp of predicted downstream sequence, was manually curated using Annotation Station (Neomorphic, Inc.). The genes for the *mat* loci of the other *Tetrahymena* species (see below) were predicted using a similar approach. In addition to evidence generated as above, we included predictions made by GeneZilla trained on the *T. borealis* genome and we used AAT to compare the six *T. thermophila mat* genes to each other.

### Identification of Mating Type Gene Pair Homologs in Other *Tetrahymena* Species

The SB210-derived sequence of the entire mt VI gene pair was aligned by BLASTN and TBLASTN to the Broad Institute assemblies of the somatic genomes of *T. malaccensis*, *T. elliotti*, and *T. borealis* (http://www.broadinstitute.org/annotation/genome/Tetrahymena/MultiHome.html). The same sequence was aligned to the *T. pyriformis* strain GL somatic genome sequence (W. Miao, unpublished data). These matches allowed us to delineate mating type gene pair homologs in the sequenced strains of all four species. Since the strongest matches were to the TM exons at the 3′ end of each mating type gene, the first in-frame stop codon after the end of the matching segment was tentatively defined as the 3′ end of the gene. The entire four gene pair sequences were then extracted and subjected to phylogenetic analysis in conjunction with the somatic gene pair sequences of *T. thermophila* strains of every mating type (SB1969, mt II; SB4218, mt V; SB210, mt VI; SB4214, mt IV; SB4223, mt VII; and SB4218, mt III). The entire gene pair sequences were aligned with ClustalW. Phylogenetic analysis was done with Maximum Likelihood, implemented in RAxML 7.2.8, Model = GTR+Gamma, 100 bootstrap replicates.

### Sequencing of TM Exons from Newly Differentiated Somatic Nuclei

The TM exons of somatic mating type genes were PCR amplified from progeny cells before the newly differentiated somatic nucleus has undergone its first division (Figure S1, stage 3). Two independent matings were necessary to avoid amplifying parental somatic TM exons from cells in the culture that failed to mate. Exons of mt IV, mt V, and VII were obtained from the DNA of progeny from a SB210 VI mating SB1969 II. Exons of mt II, mt III, and mt VI were obtained from DNA of progeny from a SB4216 IV mating SB4224 VII.

PCR primers were designed to amplify mating type-specific segments whose truncated tm had been joined to a full-length TM exon generating the complete somatic gene. Unlike the other mating type genes, the *MTA2* and *MTB3* genes have a full-length TM exon in the germline as well as in the somatic nucleus. The somatic and germline products for these two genes can be distinguished from each other by IESs, which are only present in the germline (see Table S4). In Table S2 primers for *MTA*-TM amplification begin with an A while those for *MTB*-TM amplification start with a B.

For PCR amplification of the *MTA*-TMs from the somatic nucleus, the primers and size of resulting PCR products are as follows: *MTA2*-TM: Two rounds of PCR, first round to amplify a product specific to the somatic nucleus - Primers A2, A1, Germline 10.9 kb Somatic 5.7 kb. The 5.7-kb product was gel purified with the QIAquick Gel Extraction Kit (Qiagen), and used for a second round of PCR to amplify a smaller TM exon product using primers A3, A4, Germline, and Somatic 1 kb; *MTA3*-TM: Primers A5, A6 Germline 81.4 kb Somatic 2.6 kb; *MTA4*-TM: Primers A7, A8 Germline 52.5 kb Somatic 1.99 kb; *MTA5*-TM: Primers A9, A10 Germline 21.5 kb Somatic 1.38 kb; *MTA6*-TM: Primers A9, A11 Germline 36.5 kb Somatic 1.38 kb; *MTA7*-TM: Primers A9, A12 Germline 63.4 kb Somatic 1.22 kb.

For PCR amplification of the *MTB*-TMs from the somatic nucleus, the primers and size of resulting PCR products are as follows: *MTB2*-TM: Primers B1, B2 Germline 74.7 kb Somatic 1.73 kb; *MTB3*-TM: Two rounds of PCR, first round to amplify a product specific to the somatic nucleus – Primers B3, B4 Germline 16.9 kb Somatic 11 kb. The 11-kb product was gel purified with the Qiagen QIAquick Gel Extraction Kit, and used for a second round of PCR to amplify a smaller TM exon product using primers B5, B4 Germline, and Somatic 3.0 kb; *MTB4*-TM: Primers B6, B2 Germline 30 kb Somatic 1.8 kb; *MTB5*-TM: Primers B7, B2 Germline 58.1 kb Somatic 1.7 kb; *MTB6*-TM: Primers B8, B2 Germline 42.9 kb Somatic 1.8 kb; *MTB7*-TM: Primers B9, B2 Germline 16.8 kb Somatic 1.7 kb.

Matings were carried out as for the knockout strains [42]. Starved cells of different mating types were mixed to start mating; after 24 h in starvation medium they were lysed and whole cell DNA prepared as described previously [47]. PCR products were amplified with Finnzymes Phusion High-Fidelity DNA Polymerase and cloned using the Zero Blunt TOPO PCR Cloning Kit for Sequencing (Invitrogen). Plasmid DNA was isolated using QIAprep Spin Miniprep kit (Qiagen). At least two clones of each PCR product were sequenced by Sanger dideoxy sequencing at Eton Bioscience Inc. CAP3 [54] was used to assemble exon sequences from multiple reads. ClustalW with default settings at EBI (http://www.ebi.ac.uk/Tools/msa/clustalw2/) was used to determine the germline consensus sequence (Text S6) by alignment of the sequences for the TM exon segments of the six *MTA* and six *MTB* germline genes. BLASTN (http://blast.ncbi.nlm.nih.gov) was used to align assembled sequences to the consensus sequence and thus identify polymorphic sites.

### Sequence Availability

NCBI Accession numbers for the complete sequence of the *mat* locus from the germline of SB210 and SB1969 and of the somatic *mat* gene pair for one strain of each mating type are listed in Table S3.

## Supporting Information

Figure S1 **Events of the** ***T. thermophila*** **life cycle related to mating type determination.** (1) Starved cells of different mating types (II and VI are shown as an example) have paired to initiate conjugation. (2) Cross-fertilization. A diploid fertilization nucleus has been formed by fusion of a haploid gametic nucleus from each parent cell. The fertilization nucleus will undergo two rounds of mitosis, generating four genetically identical diploid nuclei which differentiate into two polyploid somatic nuclei and two diploid germline nuclei. Before separation of conjugants, the parental somatic nucleus and one germline nucleus will be degraded. (3) Separation of conjugants. Each differentiating somatic nucleus has eight to 16 copies of every somatic chromosome [55]. Roman numerals represent chromosomes encoding the indicated mating type, only two copies are shown. Mating type is determined randomly, as shown. After nutrients are restored, the somatic nuclei will undergo additional chromosome endoreduplication and cell division will distribute the newly differentiated somatic nuclei, one to each daughter cell. (4) Vegetative phase. The thin arrow indicates the newly differentiated somatic nucleus being followed; it contains ∼45 somatic chromosome copies (four shown) encoding mt-IV or mt-V. The somatic nucleus divides amitotically (random distribution of chromosome copies). (5) The cell has undergone over 100 fissions. Successive amitotic divisions of the somatic nucleus have resulted in phenotypic assortants (all somatic chromosome copies carry the same allele) that are either mt-V (shown) or mt-IV (not shown); these cells are mature and express a single mating type. For additional details about conjugation see [7].
(EPS)

Figure S2 **The** ***mat*** **locus maps to a ∼300-kb somatic chromosome segment.** Based on meiotic recombination frequency, the *mat* locus has been genetically mapped to a linkage group on the left arm of germline chromosome 1, just to the left of the PM08 RAPD polymorphism [17],[56]. Two additional polymorphisms are tightly linked to the *mat* locus (“4711 indel” and “4711 MaeI SNP,” this work). Both map to the left of PM08 and were identified using sequence data from inbred strain C3 SB3543 (Trace archive at NCBI, database *Tetrahymena thermophila* C3 sb3543-WGS). When the somatic genome was sequenced, the PM08 polymorphism was mapped, by sequence alignment, to 111-kb scaffold 8254606 (top diagram, Somatic) [15]. This scaffold represents only a fraction of the entire somatic chromosome. Using HAPPY physical mapping [16], the entire 823-kb assembly of the somatic chromosome carrying PM08 (superscaffold 8254817, not shown) was subsequently accomplished (unpublished data). The top (Somatic) diagram shows the component scaffolds of superscaffold 8254817 in the ∼300-kb segment putatively containing the *mat* locus: 8254711 (telomere-containing, NW_002476476.1), 8254388 (NW_002476171.1), 8254758 (NW_002476517.1), and 8254606 (NW_002476376.1). Every HAPPY link (thin lines between scaffolds) was supported by a LOD score >5.5. The HAPPY links were independently verified by sequence alignment with germline supercontig 2.76 (*Tetrahymena* Comparative Sequencing Project, Broad Institute of Harvard and MIT, http://www.broadinstitute.org/), as indicated in the bottom (Germline) diagram. The actual location of the *mat* locus, as determined by this work, is indicated for both assemblies.
(EPS)

Figure S3 **Northern blots shown in** **Figure 2** **.** Uncropped Northern blots.
(EPS)

Figure S4 **Construction and testing of somatic mating type gene knockouts.** (A) Diagrammatic representation of three knock-outs. The coding sequence (black boxes) of *MTA6*, *MTB6*, or both genes together was replaced (knocked out, KO) in the somatic nucleus (*MTA–*, *MTB–*, and MT–, respectively) with a neomycin *(Neo*) cassette (diagonally hatched box) that confers paromomycin resistance (see Methods). Arrowheads represent PCR primer pairs used to test the KO strains. (B) Verification of complete assortment of the KO allele in the somatic nucleus. PCR primers (black arrowheads) within each gene failed to amplify the corresponding wt gene product (*MTA* 372 bp and *MTB* 560 bp) in every KO. The primers bracket IES within *MTA* and *MTB* so that the silent germline wt gene, still present in the KOs, gave the PCR products of 4.5 kb and 4.9 kb (arrow). Primers (one within the Neo cassette and the other beyond the flanking targeting sequence built into the construct, grey arrowheads) amplified a product that verifies replacement of the mating type gene(s). (C) Single gene KOs did not abolish transcription of the remaining mating type gene. Reverse transcriptase-PCR using primers flanking introns (arrowheads) failed to detect transcripts from the gene (s) that was knocked out. The *MTA6* primers only detected transcript for wt and the *MTB6* KO (mRNA 401 bp, DNA 671 bp), while the *MTB6* primers only detected transcript in wt and the *MTA6* KO (mRNA 733 bp, DNA 947 bp).
(EPS)

Table S1 **Strains.** *gal1-1*, partially recessive allele of *GAL1* that confers 2-deoxygalactose (2-dgal) resistance; *chx1-1*, dominant allele of *CHX1* that confers cycloheximide (cy) resistance.
(XLS)

Table S2 **Primer sequences listed by experiment.** Primers named by position within the *mat* locus of the germline or somatic nucleus.
(XLS)

Table S3 **GenBank accession numbers.**
(XLS)

Table S4 **Coordinates of features of the germline** ***mat*** **locus of SB210.** Numbering of nucleotides begins with the stop codon of *MTA2* and ends with the stop codon of *MTB3*. Roman numerals, mating type-specific regions, including *MTA* and *MTB* coding regions and putative bidirectional promoter; GLS, germline-limited sequence between gene pairs. TM, complete transmembrane exon; tm, truncated TM exon segment.
(XLS)

Table S5 **Polymorphisms found within germline copies of the** ***MTA2*** **-TM exon and** ***MTA-*** **tm exon segments.** Changes relative to the germline consensus sequence of the *MTA*-TM exon are listed in order of position (Text S6). For position in the germline *mat* locus, numbering of nucleotides begins with the stop codon of *MTA2* and ends with the stop codon of *MTB3*. For the somatic *mat* locus, numbering of nucleotides begins with the stop codon of the *MTA*-TM exon. del, deletion.
(XLS)

Table S6 **Polymorphisms found within germline copies of the** ***MTB3*** **-TM exon and** ***MTB-*** **tm exon segments.** Changes relative to the germline consensus sequence of the *MTB*-TM exon are listed in order of position (Text S6). For position in the germline *mat* locus, numbering of nucleotides begins with the stop codon of *MTA2* and ends with the stop codon of *MTB3*. For the somatic *mat* locus, numbering of nucleotides begins with the first nucleotide of the germline consensus *MTB*-TM exon. del, deletion; ins, insertion.
(XLS)

Table S7 **Comparison of sequenced** ***MTA*** **and** ***MTB*** **haplotypes derived from 0- and 120-fission cells.** The data summarized in this table are derived from Texts S7, S8, S10, and S11. Summary of homogeneity chi-square tests of statistical significance among the totals in each category: (1) The differences between *MTA* frequencies under all comparisons are not significantly different (probability >0.05). (2) The differences between *MTA* and *MTB* under all comparisons are highly significant (probability<<0.01). (3) The differences between *MTB* at 0 and 120 fissions under all comparisons are highly significant (probability<<0.01). ^a^All haplotypes showed the minimum necessary (zero) exchanges; counted as effectively 100%; ^b^Two haplotypes had a gratuitous exchange with another mating type; counted as having more than one exchange (more exchanges than necessary); ^c^The two genes considered in each case are: (1) the germline gene contributing the mt-specific segment and (2) the germline gene contributing the full-length TM exon (either *MTA*2 of *MTB*3, respectively). A few cases were found where a polymorphic nucleotide could be derived from more than one mating type gene. If possible the source gene was chosen that minimized the number of germline mating types contributing to the haplotype. ^d^All haplotypes had the minimum necessary (zero) exchanges; counted as effectively 0%. ^e^Counting gene conversions was somewhat arbitrary and was limited by the number of naturally available DNA polymorphisms among germline TM and tm exons. Two consecutive exchanges involving the same two mating type genes (for example mt III to VIII and back to III) were counted as gene conversions. Consecutive exchanges involving a third mating type before returning to the first (for example III to IV to VII to III) were not counted as gene conversions.
(XLS)

Text S1 **De novo assembled partial transcript for mt V.** See Methods for additional details.
(DOCX)

Text S2 **Multiple sequence alignment of MTA and MTB predicted proteins.** The TM regions of each protein are highlighted in yellow. (A) The MTA proteins were aligned using Clustal Omega (1.1.0) [24],[25]. (B) The MTB proteins were aligned similarly. Key: * (asterisk), positions that have a single, fully conserved residue; : (colon), conservation between groups of strongly similar properties, scoring >0.5 in the Gonnet PAM 250 matrix; . (period), conservation between groups of weakly similar properties, scoring ≤0.5 in the Gonnet PAM 250 matrix.
(DOCX)

Text S3 **Multiple sequence alignment of the furin-like repeats in the TM regions of the predicted MTA and MTB proteins.** Clustal Omega was used for the alignment. Key: aqua, cysteine residues; yellow, other amino acids shared by every furin repeat-like domain. Symbols in the bottom row are as in Text S2.
(DOC)

Text S4 **Predicted amino acid sequences of mating type genes from** ***T. malaccensis, elliotti, borealis*** **, and** ***pyriformis*** **.**
(DOC)

Text S5 **Sequence alignments of predicted mating type proteins from different species.** The MTA and MTB proteins from sequenced *T. malaccensis* (Tmal) or *T. elliotti* (Tell) strains were aligned to the MTA and MTB proteins of *T. thermophila* (Tthe) using Clustal Omega. In each case, only the mating type proteins with the highest degree of similarity are aligned. TM exon regions are highlighted in yellow. Symbols in the bottom row are as in Text S2.
(DOC)

Text S6 **Germline consensus sequences for the transmembrane exons of** ***MTA*** **and** ***MTB*** **.** Polymorphic sites are indicated by red font.
(DOC)

Text S7 **Collapsed alignments of germline and exconjugant somatic** ***MTA*** **-TM exon sequences.** Exconjugant progeny cells had not yet undergone their first division (Figure S1 stage 3). See Figure 6 for an overview of these results. The 3′ end of each exon sequence is at the left of the alignment, to preserve genomic orientation relative to the *MTB* gene. Alignment numbering (written vertically) begins at the 5′ end of the germline *MTA2*-TM exon. To collapse the alignment, extended regions of identical bases in all exon sequences were replaced with a single dot. Deletions are represented as “-”. Bases shown are those that deviate from the germline consensus sequence (Text S5). Bases in yellow are unique to a germline mating type tm segment. The switch from dots to blank spaces indicates the end of a tm segment. Germ, germline sequence. First row below numbering: consensus sequence of germline TM exons, followed by the germline TM/tm exon sequences in order as found at the germline *mat* locus. Rows beginning with *MTA*: somatic TM exon sequences grouped by mating type. x followed by number, number of sequenced inserts having that sequence. *, location of a base not present in the germline; these changes could be due to either PCR errors or replication repair errors. *MTA2, 1065 C>A×1; *MTA3, 421 G>A×1. Somatic TM exon sequences are from progeny of parent strains SB210 and SB1969.
(DOC)

Text S8 **Collapsed alignments of germline and exconjugant somatic** ***MTB*** **-TM exon sequences.** See Figure 6 for a diagrammatic overview of these results. Exon sequences were aligned as in Text S7. Symbols are as in Text S7. *MTB2, 134 C>T×1; *MTB3, 1365 T>C×1; *MTB5, 1368 G>T×1; *MTB6, 583 del ACA ×1; *MTB6, 977 G>T×1.
(DOC)

Text S9 **PCR template switching could only have affected the apparent diversity of TM exon haplotypes in 24-h exconjugants.**
(DOC)

Text S10 **Collapsed alignments of the somatic** ***MTA*** **-TM exon sequences from mature strains.** SB210 and SB1969 are the parents of the SB4200's F1 cell lines. Soma, somatic nucleus. Orientation and symbols are as in Text S7.
(DOC)

Text S11 **Collapsed alignments of the somatic** ***MTB*** **-TM exon sequences from mature strains.** Orientation is as in Text S8 and symbols are as in Text S7.
(DOC)

## Abbreviations

IES: internal eliminated sequence
mt: mating type (used only in reference to a cell's mating type)
TM or tm: transmembrane
wt: wild type
