# Correction: Selecting One of Several Mating Types through Gene Segment Joining and Deletion in *Tetrahymena thermophila*

**DOI:** 10.1371/journal.pbio.1002284

**Published:** 2015-10-21

**Authors:** Marcella D. Cervantes, Eileen P. Hamilton, Jie Xiong, Michael J. Lawson, Dongxia Yuan, Michalis Hadjithomas, Wei Miao, Eduardo Orias

The following information is missing from the Funding section: WM received a grant from the National Natural Science Foundation of China (91231118).

The funding statement is now as follows:

**Funding:** This work is supported by the National Science Foundation, USA (MCB–1025069) to EO and (U.S. NSF IGERT DGE-02-21715) to Linda Petzold in support of MJL; Knowledge Innovation Program of the Chinese Academy of Sciences (KSCX2-EW-G-6-4) and National Natural Science Foundation of China (31071993 and 91231118) to WM; Tri-Counties Blood Bank Postdoctoral Fellowship to MDC. The funders had no role in study design, data collection and analysis, decision to publish, or preparation of the manuscript.
