# Supplementary material for: Selecting One of Several Mating Types through Gene Segment Joining and Deletion in Tetrahymena thermophila
Source: PLoS Biol. 2013 Mar 26;11(3):e1001518. doi: 10.1371/journal.pbio.1001518 (PMC3608545; doi:10.1371/journal.pbio.1001518)
Supplement: Text S1 — De novo assembled partial transcript for mt V. See Methods for additional details. (DOCX) [file pbio.1001518.s012.docx]

**Text S1. *de novo* assembled transcript for mt V**

GAAACCAAACACTTAGATAATTTTAAATCTTTAAGAAGTTGCAGTAAGCTAGCTAAACTAAACATAAAATATGACAATATCAGTGCTTTTATCAAATAGAATAATTAATACTTTAAATAAAACTATAGATGCTTTGAAAAGTTTAACTTTTTAATAATGGATTACATTTTAAAAAGATAAATAGCTAGAAGTATCTAATACTTTGCTAGGATTTTAAAGAGTTAATTATACTTTTGCTATTTAATCTTTGCAATTACCTAAAAATTTAAATAGCAATTATTACCTGTCTTTGACTTTAGATCCCAATATTTAATTTAATACAACAGAAGTCTATTGTTCTCAACTTATACTAACAACTGATTCAAGTGGAGTGAATCAAGTTAAAGAAATATCTTTAAATTGTATTTATAATTTCTTTAACCAATTTCTGATAGCTATTTAGAGCATTTCAGTTTCTTAACCCTTTTAACTTAGAATAAGTGGTTTGAGAAATCCCTCTGGTATTGTTGAATAAAATGAATAAAATGTAGCCTAGACATATAATTTTTAATTGATATGGAGTACACAATAAGGAAATAGTTTCAAAAATGTTTGGGTTATATAATCAGTTAGTCTCCCAATCACTTCCAAATATACTTGCTCACCCAATTGCTAAGCTTGCGCATCAAATTACGCTGCTTGTACAGCTTGTGCTCCTGGATATCTCAAGTCGCAGTACAATCATCACGCCGTCCTTGCTTGTCTTCCTACATGCAGCCCTTAATATGTAGCCTATAATGGTACTTGTTTAGCTTGCTAGCTAAAAGACCCTTAATGCTTGTCATGTTCGCCTTCAAATCTCACTTAATGCTCAAGCTGCAATCAAGGATATACGCTGGTGCCTGAGTTCAACGGGTGCGTCGATAGTCACTTGCTTCAGACAGCAAGATCAAGATTATTAGATTACTCTTTATCTACAAATCTTGCAGATAATATAGACACTACTCATCCAGATGATGCACAAAAATCAGACAAAGCGCAATCCTTGACTAGAATGACTGAAGAAAGTTCAAGTGCTAAGGCCGATTAAAGGGAAGGCGGCGAAAGCAGCAGCAATACTGGCAGTAAGGTTATGGGCCAGTTGCAAGATACTGTAGGCGCTTTAAAAGGAGGAGGAGCAATCTTTATATGGATAGTGTTGGCAGCATTGGCAGTGAGTGTGAGTTAGAGCGTTCTCAGATACACCTACAATAAATTGAAAGGGAAGAGTCATTCGAATGGGAGCAGCAGCGGCATGAGGAGGAGCAGCAGCGGCAGTAGAAGCAGCAGCAGTGGTAGCAGTAGCGCTGGAAGGAATGCGAGTGGGAAAGAGCATAAAGGAGTGAGAGAAAGAGAAAAGGAAT
