# Supplementary material for: Selecting One of Several Mating Types through Gene Segment Joining and Deletion in Tetrahymena thermophila
Source: PLoS Biol. 2013 Mar 26;11(3):e1001518. doi: 10.1371/journal.pbio.1001518 (PMC3608545; doi:10.1371/journal.pbio.1001518)
Supplement: Text S2 — Multiple sequence alignment of MTA and MTB predicted proteins. The TM regions of each protein are highlighted in yellow. (A) The MTA proteins were aligned using Clustal Omega (1.1.0) [24],[25]. (B) The MTB proteins were aligned similarly. Key: * (asterisk), positions that have a single, fully conserved residue; : (colon), conservation between groups of strongly similar properties, scoring >0.5 in the Gonnet PAM 250 matrix; . (period), conservation between groups of weakly similar properties, scoring ≤0.5 in the Gonnet PAM 250 matrix. (DOCX) [file pbio.1001518.s013.docx]

**Text S2.** **Multiple sequence alignment of MTA and MTB predicted proteins**.

**A. MTA protein**

MTA3 -----------------------------------MMILIILICSL-FGALRSAKSLDQG

MT6 -------------------------------MLFLLVILNISIQ--QTLCDWRFVQILDG

MTA7 MKFYLLTFLVKCFIAFQIQFTIQAKNNLCYHLIFYQSLKQILVSRAFSYFDFNYFNIDRG

MTA2 ----------------------------------------MIVT---IVICSDIHFIRNG

MTA5 MKVLLLVA-----------------------------LLNIFIGLVK--PITRFYYTDLG

MTA4 ------------------------------------------------------------

MTA3 IIIEVKH-GDYKFIKIVTNDESSIKNFNGKLDQLNSKIFTINQIVFQQPKE-DYCFLPSP

MT6 LILQIKNDLSLAHVQIVTNDESAITNSTVNQDQF-QNVQIVNQLWFWAKNQ-QNYLLPFS

MTA7 IIFQFYT-LDLIYIQITTNDFASLNNSTL-NNYFSSKMMILSQPSFSIKQN-QTCFLPLA

MTA2 IMFQYTN-QNAKSFEIITNDMSPLSSATTQPDQYKQNVYVVNSLKFPQQQNNKYFFLPFP

MTA5 IVVQVYN-DTLTNVQIITNDEESILNNTIKSDQLNRNIQQINQLWYQQKDS-SFYLLPFS

MTA4 -MIQAFN-QGLTYCEVITNDEASILNQTKSFQNFNDKVYKVNQLAFWSKNL-NNTFFPFA

:.: :: *** : . . : :: :.. : . . ::*

MTA3 QNTFGRIISQEFYDENMNLINSAISNIHGQVLIKNTTQKLIDAGNGQKWLSVILNAY-QQ

MT6 SNTFKRRMTYNFSKDQLMGASEASS--------SQLLQTLINN----LKLSMT------Q

MTA7 QNTYNRVVGLDIYDRNNNLLYNLQVKNKVLIKLT--TQKLVYNQDNSMFLSLTFNVF-EQ

MTA2 PNTFNRLLSVKCLDQNENIIDQYFLQNIV--TISSQIQQLINSEDNNKYLQLTLSSSNNA

MTA5 LNTFKRTIIFNYYDSNMVLKGTGGISNYIKAKLNQILIGEVQS--NQAQLLMQISTI-KQ

MTA4 QNSFERNISLNFYDASMNFLGTSYIYSK--VMIKKVLQNYIQDANNNKMLQISLQTY-GQ

*:: * : . . . . : * :

MTA3 FQIISNQINSIFQIYTTIPNLTTNQSAQSIFSLLIDGKKMNCFFNQTNTGIKNGTLFTQN

MT6 MKITIS----------------KSKYK----------QQINSFQSCQS---NMLFSICQC

MTA7 FRIIDPNLQEY-FQIQISPQNKSQTYPQYQFRLLADNQEISSYQTVQGY-----SYFAKQ

MTA2 FRAIQSSTSGN-FKVQFTVENTLKSYPQNFINLIIDGQNITCIQSSYQITFRNQVQYTQY

MTA5 FKIINNNQQDDFQVSFKLSNTVQFPYPDKIFDFMIDGQKMAYSQCFKQVLYNNLVVFNQI

MTA4 FQIIDKTNFYDFFQIKFYTNSALLSSPQDLFSLQADGQTINSYSSYTEL-FITQVIYVKN

:: : : :

MTA3 FYLSDFPTQFQDLEIQFFNPFFPTFSLSISS-----GADFLQFQISNQLYSSYLLYNSNP

MT6 FKQTDFPQQFKNLEFVFQQYLISSIQIKMQLYNQVSSFESIDISTFNQFYSVQYLLSNDM

MTA7 YNQSSFPTQFTNLEIIFSDYSFQTVYLQLAL-----GAAQTSMTIYNQYYSVQQLQIANF

MTA2 FNYSEFPSQFQNFGFQFYGYFFLGFNLQFSV-----GYDFNVIQGWNQFYSIYVIQNQTN

MTA5 FQQSDIPISFQKMQLVFNQYQFQQFTIKIIF-----GSDMIQFDNFNQHFSTQFLMSISL

MTA4 FNQSDFPDKFQNLQLIFSEYRFQNINLQILF-----GSTNIKVTDYNQYYSALQLQNNNP

: :.:* .* .: : * : . :.: . . ** :* :

MTA3 SAMIIPQS-DNLIANQLVFILQSDIFSQCYVQQLDLPSNMNTQFSFSQPVLNVQNPQNRS

MT6 QALVRPSSLGKLLAYELAFNLNFTIFVNCDLYQIILPPSISQQFTKAAPTLMAQFQTNSN

MTA7 NALIKPAPQTNFLGNEIDVQLQFSIYQQCSLSQIIFPPEISAQFSQTQPVLQLQNVINNQ

MTA2 LAYVRPVQYGDFIANELAFNIQQSAFNFCNLQQIVLPQSISDQFQAIDPILLLQNTVNQN

MTA5 NATVNPSSQFNQIYNQIYFNIDQTVFFQCSIKQIILPPLLQSQYTNQLPSLIITNSQNSN

MTA4 QAIIRPSSFLSQIKNELAFNITDVVFLQCLLSSILLPSEFQNEFITAEPSLFVFYPTNKT

* : * . : :: . : : * : .: :* :. :: * * *

MTA3 QSSIIDSNFY-NGTISFVRNQNLRVLPSQVSYSDTLVAIS--SFLDVIGFDNNLTMTFVC

MT6 -FTEQVTDYYNNGTLILQKSLSFKSSNTLN---NIQILIQQFEFQDQIDDNSSSSIVFNC

MTA7 -IQYLETTFD-SRTLNLKQKQNTLQQQD-N---SNVLVIQ--SFNDVVGINKNLSFVFNC

MTA2 NFTSQEIIYVNNGTIMF-KNQNLQIISQLN---PIPFILK--QFVDVVGFNQNLSLTFSC

MTA5 -TTLMSTDYN-NGIISLQQTQVFQQFKYQA---YYSIIIT--PFNDVVGQNMNLTATFNC

MTA4 -FQNQETYYM-QGNISLKVSQNFFPNRANN---QIQILIS--PFSDIVGQNKQLNVAFNC

: . : : . . : * * : : . . .* *

MTA3 SQNLIQKKSIRIIKNEYLNANQTQVSIQVNNLVGTKNISIQISIPSVTIQNTSLIMQLPQ

MT6 SQNNLFVKNFTISKIQLPNANLTQTQIVLNNTD-IPTLNIIIQIPPINILSSVLTLELPN

MTA7 FQNSQFIKNVTLSATTYSNSNQTSALLQFYNYD-QKNLTINISIMKIYIQNTSLFLRLPQ

MTA2 NQNQLITKNYSIIQNEFLNANLTQALVQLNNNGNTPNLTIQIQIPVVQVQNSSFYLKLPL

MTA5 PQDVQVSKNITIYQSEFPNANQTITQIQFNNSG-IKNLTISIQIPRVNVLNTSLYLQLPQ

MTA4 QNNIQTIKNISISSNELLNANQTQTQIKFDNNQ-VKMLSINIQIPQVQVSNTSLFIQLPY

:: *. : *:* * . : . * :.* *.* : : .: : :.**

MTA3 SILFQDNTQQSLDVQGFVYSNFVWQQNNLAFFGANFSSTILRLSFTNIDVKNQTSNVDIL

MT6 GVQCLNQSQTNILTNGFVYSQYQSIDNSISFLSTNFTSASISITFTKVNKID-----GNQ

MTA7 GISYLDQTQNSILVNGFVFSQFQWQQGIISFSQANFSSTSIQISIKNVQLNVQSN-G-GY

MTA2 GINFLAMDQSQVSVQGFSFSQLNWSQNIISFLQSNFTSTSILISVSKVELQKQSN-G-KQ

MTA5 EVDYQQQTQQSIIVQGISFSQFNWNEQIIYFGQTNFSSNVISFQILNVQLKNSSN-QTSF

MTA4 GIDYLQMNQSSLSVQGFAYSQFNWDQNLISFYSTNFTSSSILISIKQVQLSSSSN-N-IQ

: * .: .:*: :*: : : * :**:* : : . :::

MTA3 NSTFIIAKCIFNQTFVFYVDSNTNQTVKIIQPT-PLPRSQITLSSFNQTKVNSLDVPESR

MT6 NLTKINVKCIFNSTVLFYVDANTQQTITIIQPPSPPPQTQIQINSFNQTSFSSLSIYENR

MTA7 NISTILVKCIQNSSFVFQVDSNSKQSYQIFELP-SPPTTLIKINSFNQTNANSLNILENR

MTA2 QITKIIAKCVFNSTFVFYVDENTNQTLQIIQPS-PTPITQIQINSFKQTSDVSLDIPEDK

MTA5 KINKIMAKCIFNQTFVFYVDSNTNQTTQIIQPT-KVPITLIQINNFNQTTVNSLSIPENR

MTA4 NVTKIIAKCILNQAFVFYVDSNSNQTIQILQPQ-PLPTTSIQINTFNQTQSTSLSSPQNR

: . * .**: *.:.:* ** *::*: *:: * : * :..*:** **. :.:

MTA3 TSVQSQIAFSFSVLNFLDTCSWLIVQIPFEFTIGFIQMSQFTLQDCFGNIYSYLQGSSIL

MT6 TSVLSQLAFSFSILKFLETCSWLIVSLPLEFTTSFQQNSSFNLFGCHGNNYQYTQVNPLS

MTA7 TSVQSQLIFSFSILQFLDTCSWLIVTIPNRFTIGFLKQSQFNLQDCVGNSYSYLQGNPIS

MTA2 TSMMSQLVISFSILKFQSTCSWIIIDLPQDFRIGFIQQSQFSLQDCFGNSYTYLQGSSIN

MTA5 QSVQSQLAFSFSILQFQDSCSWLIVSLPLEFTLNFMQQSVFNFQDCYGNTYSYQNGSPLS

MTA4 TSLQSQLAFSFSILNFQDTCSWLIVSLPYSFTIGFLDKSQFTLFDCFGNTYSYTQGNPIS

*: **: :***:*:* .:***:*: :* * * . * *.: * ** * * : . :

MTA3 PQSMISYTDNNNCMYISCSSLRLNSQM---NSSSQCMNNTVIIQSIKSPDQAVQTSSLNI

MT6 AQSVIGYTQNNNCIYISCLALQQFSQLNNNNNNNYCLNNTVTIQNVKSYDLATQTQGLKI

MTA7 YQSVISFTDNNNCIYISCSALKLVSQS---NNNNQCQNNIITIDNVKSPDLPTQTESLNL

MTA2 SQQTLSYTDNNSCIYISCQTLRQIAQN---HNTDQCLNNALTIKNVKSPDYATQTDNLNL

MTA5 NQSPIGYTDSNQCIYISCLNLRILSQA---NQSNQCLNNTVIIQQVRSPDLPIKTWSLDF

MTA4 SQSTIGYTDNNNCIYISCKALRSVSSS---HAANNCLNNTVTIRNVRSPDFPLQTAGIQF

*. :.:*:.*.*:**** *: :. . . * ** : * .::* * :* :.:

MTA3 YIANQNSSAPSNIGPPTFFTQNLNLKNNSLPYFFIPTEVYSHVGINITEADFSDLNFNIS

MT6 FVANRNSSNKQDLNPPTFFSENLDLNNNSLPYYFTNSEVSTYQGINLTEVDLSYLAFNTI

MTA7 IIANSKTPAQANNFPPTFYTSSLNLSDITLPYLFFSSEQQRYQGISLQQVDLSSLSFYAS

MTA2 YIANSYSSTPQNNYQPTFFNSSLNFTDSSLPYYFNPEEVFSYQGINVKPFNLSALSFQIS

MTA5 FVANSNSSMQSNNNPPIQFNRSLDLQNNSLPYFFIPTEISTYQGINITNFDLSQFSSQIS

MTA4 LIANSNTSSQPNSDPPTFFNQSTDLTNSSLPYFFIGSEVFTSKGINISQVDLSTLSTQIS

:** : : * :. . :: : :*** * * **.: ::* :

MTA3 SNYYGDVFNLTTTISYPFYLYQQHQISIQIPVRIL-ANQSVVCYPESFLSCSIKIPINLN

MT6 STYLRDIFNFTMTFSYPIYLWEKHHLNIQLPYQFL-GSNNITCQPASFVQCSLQMGTQQN

MTA7 SNYLGDVFNFSMTFSFPIYFSQQHKINVDLPVKLL-ANQTVQCFPQSKIQCSLQIGDQQI

MTA2 SNYYRDIFNFSMIFSYPIYLFPQHKVIVLLPFKVL-INQNLTCFPA-NIQCSLSIISQDI

MTA5 SNYQGDVFDFQMILAFPIYFWDQHLISVQVPFKIL-GNNSLSCQPSTNVICSILNIKGLD

MTA4 SNYFGDVFNFSMILAYPIYFWEQHQIDVQLPFRLFAGKSDIECQPISTVVCSLQASRDPQ

*.* *:*:: :::*:*: :* : : :* :.: .. : * * : **:

MTA3 F-TTILIQILQQVSPNTKIAFQLNQVVASQSLLQGKQAFALIQVSSTSKIINTKNKTIDV

MT6 Y-STLNLLILQQVLPDTKITIEVYQVVVVAENTI-QQKQAVIKLFLENRLINTKSKIIDV

MTA7 Q-TTIQIIFVDQVIPDIKITLQLFCAALQQSNQQNQQFFSLIRIFQQNQIINTQNKTIDV

MTA2 YPTSVLIQFTNQIL----------------------------------PSINSQNKTLDV

MTA5 F-TNVQIQIINQMKPNTQIILNLQEVAVSQ---LNQTQNMTISVLLSNRIINTLNKTIDA

MTA4 S-TIFRIQVLNLTAPNTQIGLQVSYVVLSQNFQLDQISFLIISVSLSNRIINYQNKTIDA

: . : . : ** .* :*.

MTA3 LNNLKLQSWINIDAIQPLQISNTLLGQEGVNYTFAILSLQIPSNQLDNYYLSLKIDQSLS

MT6 LSDLMPQNFIVFSTSSPLQLSNSLLGLSDVNYTFFIKQMNLPPKINDNYYLSLTLDQSFL

MTA7 FSNFTTTQWITFST-TPLQISNSLLGIQGNNYTFLISKLFTPPNLTENYFLGLELDSSLQ

MTA2 LTNSTQQNWINFENNLPLQLSNSLLGISGVNYTFFIQSLYLPQNLNSNYYVKINLDQNIQ

MTA5 LKSLTFQQWITFQKDKQLEVSNTLLGFQRVNYTFAIQSLQLPKNLNSNYYLSLTLDPNIQ

MTA4 FKNLAIQNLITLQNQQPLQISNPLLGITGVNYTFNIQQLQIPQNINNYYYLSLSLDPSFQ

:.. . * :. *::** *** **** * .: * : . *:: : :* .:

MTA3 YNSSNSNCYSLTQVT---GDNSQREEKVNCINFSSNTFLIPISKN-QISQPFQLRITGLR

MT6 LVSNNYNCSILTQGE----NQIYLEQQLKCLFYFPNNFIFSISSFAIMSQPFQLRISGLR

MTA7 FNQTSLNCYLLNITLDTS---LINENKIDCFFSSMNMLILPLSKI-LTSQSFQLRISGLR

MTA2 FNSSEISFFQIIQTQDG--KMTLSENQITNFYYASQAFFIPLINL-NLSKAFQLRISGLR

MTA5 FNTTEVYCSQLILTTDSSGVNQVKEISLNCIYNFFNQFLIAIQSI-SVSQPFQLRISGLR

MTA4 YNSSQIAFYQLIQSQ---NLNTFIENRIDFISNSSNLFLIPITSL-VPSYPFQLRISGLR

.. : * : : : ::: : . * *****:***

MTA3 NPSGIVEQ---NVAQTYNFQLIWSTQQGNSFKNVWVIQSVSLPITSKYTCSPNCQACASN

MT6 NPSGIVEQNEQNVAQTYNFQLIWSTQQGNSFKNVWLIQSVSLPITSKYTCSPNCQACASN

MTA7 NPSGIVEQNEQNVAQTYNFQLIWSTQQGNSFKNVWLIQSVSLPITSKYTCSPNCQACASN

MTA2 NPSG-------NVAQTYNFQLIWSTQQGNSFKNVWVIQSVSLPITSKYTCSPNCQACASN

MTA5 NPSGIVEQNEQNVAQTYNFQLIWSTQQGNSFKNVWVIQSVSLPITSKYTCSPNCQACASN

MTA4 NPSGIVEQNEQNVAQTYNFQLIWSTQQGNSFKNVWVIQSVSLPITSKYTCSPNCQACASN

**** ************************:************************

MTA3 YAACTACAPGYLKSQYNHHAVLACLPTCSPQYVAYNGTCLACQLKDPQCLSCSPSNLTQC

MT6 YAACTACAPGYLKSQYNHHAVLACLPTCSPQYVAYNGTCLACQLKDPQCLSCSPSNLTQC

MTA7 YAACTACAPGYLKSQYNHHAVLACLPTCSPQYVAYNGTCLACQLKDPQCLSCSPSNLTQC

MTA2 YAACTACAPGYLKSQYNHHAVLACLPTCSPQYVAYNGTCLACQLKDPQCLSCSPSNLTQC

MTA5 YAACTACAPGYLKSQYNHHAVLACLPTCSPQYVAYNGTCLACQLKDPQCLSCSPSNLTQC

MTA4 YAACTACAPGYLKSQYNHHAVLACLPTCSPQYVAYNGTCLACQLKDPQCLSCSPSNLTQC

************************************************************

MTA3 SSCNQGYTLVPEFNGCVDSHLLQTARSRLLDYSLSTNLADNIDTTHPDDAQKSDKAQSLT

MT6 SSCNQGYTLVPEFNGCVDSHLLQTARSRLLDYSLSTNLADNIDTTHPDDAQKSDKAQSLT

MTA7 SSCNQGYTLVPEFNGCVDSHLLQTARSRLLDYSLSTNLADNIDTTHPDDAQKSDKAQSLT

MTA2 SSCNQGYTLVPEFNGCVDSHLLQTARSRLLDYSLSTNLADNIDTTHPDDAQKSDKAQSLT

MTA5 SSCNQGYTLVPEFNGCVDSHLLQTARSRLLDYSLSTNLADNIDTTHPDDAQKSDKAQSLT

MTA4 SSCNQGYTLVPEFNGCVDSHLLQTARSRLLDYSLSTNLADNIDTTHPDDAQKSDKAQSLT

************************************************************

MTA3 RMTEESSSAKADQREGGESSSNTGSKVMGQLQDTVGALKGGGAIFIWIVLAALAVSVSQS

MT6 RMTEESSSAKADQREGGESSSNTGSKVMGQLQDTVGALKGGGAIFIWIVLAALAVSVSQS

MTA7 RMTEESSSAKADQREGGESSSNTGSKVMGQLQDTVGALKGGGAIFIWIVLAALAVSVSQS

MTA2 RMTEESSSAKADQREGGESSSNTGSKVMGQLQDTVGALKGGGAIFIWIVLAALAVSVSQS

MTA5 RMTEESSSAKADQREGGESSSNTGSKVMGQLQDTVGALKGGGAIFIWIVLAALAVSVSQS

MTA4 RMTEESSSAKADQREGGESSSNTGSKVMGQLQDTVGALKGGGAIFIWIVLAALAVSVSQS

************************************************************

MTA3 VLRYTYNKLKGKSHSNGSSSGMRRSSSGSRSSSSGSSSAGRNASGKEHKGVREREKELRG

MT6 VLRYTYNKLKGKSHSNGSSSGMRRSSSGSRSSSSGSSSAGRNASGKEHKGVREREKELRG

MTA7 VLRYTYNKLKGKSHSNGSSSGMRRSSSGSRSSSSGSSSAGRNASGKEHKGVREREKELRG

MTA2 VLRYTYNKLKGKSHSNGSSSGMRRSSSGSRSSSSGSSSAGRNASGKEHKGVREREKELRG

MTA5 VLRYTYNKLKGKSHSNGSSSGMRRSSSGSRSSSSGSSSAGRNASGKEHKGVREREKELRG

MTA4 VLRYTYNKLKGKSHSNGSSSGMRRSSSGSRSSSSGSSSAGRNASGKEHKGVREREKELRG

************************************************************

MTA3 QRIDCLMLFLLSVAEAIQMPYTLLWGFSVSGGDFESPVLQTLLGACALSTLLWIFDAYLL

MT6 QRIDCLMLFLLSVAEAIQMPYTLLWGFSVSGGDFESPVLQTLLGACALSTLLWIFDAYLL

MTA7 QRIDCLMLFLLSVAEAIQMPYTLLWGFSVSGGDFESPVLQTLLGACALSTLLWIFDAYLL

MTA2 QRIDCLMLFLLSVAEAIQMPYTLLWGFSVSGGDFESPVLQTLLGACALSTLLWIFDAYLL

MTA5 QRIDCLMLFLLSVAEAIQMPYTLLWGFSVSGGDFESPVLQTLLGACALSTLLWIFDAYLL

MTA4 QRIDCLMLFLLSVAEAIQMPYTLLWGFSVSGGDFESPVLQTLLGACALSTLLWIFDAYLL

************************************************************

MTA3 GSILANSENTPKTSCLLNPLPSSKREGCLSFLIVPARLAFCLIPKSVSLTLTNIFSVEGW

MT6 GSILANSENTPKTSCLLNPLPSSKREGCLSFLIVPARLAFCLIPKSVSLTLTNIFSVEGW

MTA7 GSILANSENTPKTSCLLNPLPSSKREGCLSFLIVPARLAFCLIPKSVSLTLTNIFSVEGW

MTA2 GSILANSENTPKTSCLLNPLPSSKREGCLSFLIVPARLAFCLIPKSVSLTLTNIFSVEGW

MTA5 GSILANSENTPKTSCLLNPLPSSKREGCLSFLIVPARLAFCLIPKSVSLTLTNIFSVEGW

MTA4 GSILANSENTPKTSCLLNPLPSSKREGCLSFLIVPARLAFCLIPKSVSLTLTNIFSVEGW

************************************************************

MTA3 FRYPYGEDVERATIVLTNFRKVLSSQIKSNVSGLCSLVSFLILQYPEVLSSYVQIYDLII

MT6 FRYPYGEDVERATIVLTNFRKVLSSQIKSNVSGLCSLVSFLILQYPEVLSSYVQIYDLII

MTA7 FRYPYGEDVERATIVLTNFRKVLSSQIKSNVSGLCSLVSFLILQYPEVLSSYVQIYDLII

MTA2 FRYPYGEDVERATIVLTNFRKVLSSQIKSNVSGLCSLVSFLILQYPEVLSSYVQIYDLII

MTA5 FRYPYGEDVERATIVLTNFRKVLSSQIKSNVSGLCSLVSFLILQYPEVLSSYVQIYDLII

MTA4 FRYPYGEDVERATIVLTNFRKVLSSQIKSNVSGLCSLVSFLILQYPEVLSSYVQIYDLII

************************************************************

MTA3 FDIVMTILCLTNIRNVDKLISTLNQIQESEGEL

MT6 FDIVMTILCLTNIRNVDKLISTLNQIQESEGEL

MTA7 FDIVMTILCLTNIRNVDKLISTLNQIQESEGEL

MTA2 FDIVMTILCLTNIRNVDKLISTLNQIQESEGEL

MTA5 FDIVMTILCLTNIRNVDKLISTLNQIQESEGEL

MTA4 FDIVMTILCLTNIRNVDKLISTLNQIQESEGEL

*********************************

**B. MTB protein**

MTB3 ------MTDCKIFIWFYILYLISKFCKCQVDCYLASSTQIGQQIQPIAYSQRQGQT---S

MTB6 ---------MQVYTLVFVLILLIRKNSAQTICYQISADKNLKQIQQLPLNQRLEFY---N

MTB7 -----MSGLRK-VSLLMAIYQILCLSYGQLSYYLVSLNQNTKQLNPLPLNQRQISS---N

MTB2 -----MNRA---IEFLFLVILIIPIIKGQLQCYQVNLNQSQQQIKQLPLNQRDGNP---V

MTB5 MTSFFIKRKIQIVYCLFYIQCIIPLTKQQINCYKVLVSQSQTQLTPLPLNQRVEYTQVDY

MTB4 -----MNASQIIYLLIFVAALLIERSQATLSCYLINPTQSLKQIYQLPFNYRQGSD---A

. : * : *: : . *

MTB3 FGAQYFFTIQLASNQTDIL-QGTSSVQFT-QYFLTSNKFVLSSMISKNM-GLLPTPQQYV

MTB6 KIPNYNFALDFSINNSDFLANYPPSLFFTYLFITTSNKQVLDKFTTFPS--IQQIQQNYG

MTB7 NLQQYYFVVEGN-ITQSND-LMGLGLDYTYRLLTTSNRVILYYFSNSD--QLSSIPTQFQ

MTB2 IVQTNYFGFEML-LGQDSD-LNQLTFTFTE-VFLTSNRNIISNQTIFYGNIVDPPYSIIN

MTB5 QSQLYYFALEYSQLFL-NN-LPSNDIQYTYQYLTTSNRQVIKIYDNTSQ--FFGVPDQFK

MTB4 APAQYNFALQYDQLSPDVQ-YTLQNIAYLKRYLITSNNQVISIIPNTIGDSLN--YIPPE

* .: . : : ***. :: .

MTB3 NSTYYVAAAFTYQ---QFISGALDFSSICNLDQYPLLTLKEFVQVNTQTQQMFVSVPLTS

MTB6 NQAFYITLVFDSSSFQQ-AIDSQALQAYSSITFFPMLTLPIFVQINSQSQQLMISSTLIS

MTB7 QNGFYINLLVSLQFSQGFNQVMQFINSNDALVQYDQLTLKKFVQLNTQSKQFLISTVLNS

MTB2 QVKYILILVNYQQFMQNF---QQSYSTLCGISQYSQLVQKEFVQVNSQNKQFIVSTVLSS

MTB5 QATFYVTQMINLK--NLNSNQLQNFFGICALNYFAQLHIKEFVLLSQQNQQFAISFDLIS

MTB4 LIQYTFNIVTIYQSIANFSDQPDFLTSYCQITQFPQMDLTTFRQVNKQNQQFLISAILLS

: . . : : : * :. *.:*: :* * *

MTB3 QINDVYYLTLFYPTSLASQPQNNNNLGCQLYTDYIVINQCSLVINS--TLTTVSFNLSSI

MTB6 QINDVSTLVLLYPTSFASQPQNSNNLGCQLYTDFVSINQCSFATNSSSGLSSISFNLSGQ

MTB7 QTDDISNLILQYPSSLATIAQNSQNIGCQLYTDYVVLNQCSLIANPSTGLTSISFNLSSI

MTB2 QISDQATLTIQYPTSFASQSQTTSNLGCQLYTDYGNLNSCSFSTNSSSGLSIVSFDLTNL

MTB5 QTDDLSYVVLQFPTSFAQKPQTNSNIGCQLYTDFITIDKCSLTTNATSGLTSVSFNLTNL

MTB4 QIEDLTSLVLSYPTSFALKSQNAANLGCQLYTDLGIINSCSFSLNAQQGLSLVTFNLTNF

* .* : : :*:*:* *. *:******* ::.**: * *: ::*:*:

MTB3 NI---RNISATLVLSTATFNQNFIKQNQPFQIQLLNQLQNKIAQSPAFGAKDYRQCQLT-

MTB6 NV-SLQNISATIVLNSTTFIQNKLS-TVKFQMQLLNYYQSQIAQSPLFAIQDQRKCQQTT

MTB7 PI-PLTNISATVAINYSSFNQNFIQ-AQPFSLQLFNIYNIKISQSSNFGGVDQRVCQQS-

MTB2 NF-YQQNISASVILNYSTFNQQMLL-KPQFKLTLLNQYSYAIGGTPTFSILDQRICQQT-

MTB5 TQNNLQNISAALVLNYSTFNQNKFQ-NQQFQVQLLNYYSYLIANTTQFAIVDYRVCQQA-

MTB4 NI-SLQNISATIVINYTTFNQNLLS-NKQFSLQLLNQYQSQISQSSQFQIVDQSICQQT-

****:: :. ::* *: : *.: *:* . *. : * * ** :

MTB3 NFQAQLIFNNSYSISEQRLQVQFATASPAISRILIEFSSQILPKFNLTNVLVMVSDLSKL

MTB6 SFSANLIDNTMQFLQMKRLQVSFTPATQKITRLMIDFLQQVNVKTNSSNIVLILSDTSQA

MTB7 SFSAVLVNDTQAYNQRPIWQVRFTPT-TNITRILIEFSQPIIAKIKGNTIPFTLSDLSGN

MTB2 GFSSILSPSILKLNQQDKLQINFTPVSNNITRLIFEFNQQIIPKFNLTKIFITLADSSLT

MTB5 SFSAEIINNVQDFSQNERLKIKFTPASSNITRILIDLNNQAIIKINLTKIVIELSDLNQS

MTB4 TFSANLINNLQTFGQNLKLKINFIPASSNITRIIIQFSSQIYAMIELSHPQLVFIDSSQT

*.: : . . :: * . *:*::::: . : . . . * .

MTB3 NPYAVFCQKAYQSIILCELFSPASPFDMSKGVILTLPYFQPMQAVPQNSYSFTVKYFTDS

MTB6 NSFVIFCNKVYQSLVLCDLTN-YQLFDFTKGILITLPYFQTVQQVQQPTFSFQIIYYGDL

MTB7 NQFLLSCNKIYKSIILCDLYG-YIPFDNSQPIILNFPQFDIISSTLQPQIQFSFKYFTDF

MTB2 NSVITQCYKAYLTMIVCDLEF-QLTFDITKSIYVILPYLQIPSLVTLNALTLSFKYFTDS

MTB5 NSLKILCSKVYQSLILCDLYN-AFDFDITQGILMTLPFFQLTQPLQLMNITISIKYYTDY

MTB4 TKFKVRCLKAYQFLLVCDLIG-GLPFDVSQGVYAELPYIQVKVPSYPQSLTFQIKYFTDL

. * * * :::*:* ** :: : :* :: : . *: *

MTB3 TYQTCYNTNITIPFIPQD-IESPVAFLFNNDSLLLQFSNVINLETSTTINIQLPSQLSFA

MTB6 SYSNCYVTTLTVQYLPIPSLFSPIAYLYSNNTLQMSLFDKLSLDSSMFIQIQLPNLLSFS

MTB7 SYQTCYSQNLMMQFQTIV-PQSAVGFLYNNNSLLLSFSNPIQIDNQMTININLPKQLAFS

MTB2 NIQNCYATNFTLQYQAIV-AQNPLSVLYNNGSLSITLFDTISLDNSMVINLSISNQLQFS

MTB5 SYKNCYFSNTTLQFQSET-SSSANAYLYSNNSFQISISNLIKLENQMTINIVLPSQLSFS

MTB4 SYQNCFVSNLTQKFQAAI-PTSPQAYLYNNSSLQIKLFDLIMLDNSTVINLSLPSQFSLS

. ..*: . : . . *:.* :: : : : : ::.. *:: : . : ::

MTB3 PTSQLAQITGISSLSKILISSSQNSKISQVTQSLQQLTTNQGIQFQLLNVTRSSIFCSLT

MTB6 QTTQLGNTSGISNLSKISVQSQQKAQLSQINQSIQSI--QSGISFSFTNVQQINTECNYN

MTB7 ASSQLNQIFGISNLSQISIKSQQSASISQITLPLNQLNAYLGISFQLTNISQSGLYCSVN

MTB2 VSSQLIQVSGISNLSQMTYKSQQSLQISQIAQQKSQQFA-YGISFQLTQLSQSSIFCNFS

MTB5 QNVQLNNAFGFNSTSRVQVVSQQQVKISQLVQSYQFIPTYSGISFQLINVTSNTVYCNLS

MTB4 SSTQINSIVGISNQSQITLSSQQNLQISQISLSLAQLMTYQGISFQLTKIIQNTVNCNLS

. *: . *:.. *:: *.*. .:**: **.*.: :: *. .

MTB3 NIASLQIQIVNSFGVVIMSGTVPIQIQSYSIIVTSINSNQILNLQ-------Q-QDNDLT

MTB6 STNLVRIQITNTQGLLIYNITAALQIQPKQINIVSINTIYLTPDSQSQS--QQLNQYFNV

MTB7 STYQAQIQIINSLGTIILSGNISIQIQPYPIQIVSTNTIQVPQSP---------------

MTB2 NPYSVQIQIINSFGNLILTGSIPIQIQPYPITILSLNPIYMPNSDSQQM-----QNSSKI

MTB5 TQLSIQFQVINSFGLIIISGSIPVQIQPYPIQIQSTNQIYLTNPQQQ------QQNNNKN

MTB4 SLSQIQIEIVNSFGVIVLSGSIPLQIQPYPIYIQQMNAVFTPLSQQQLLQYQQQQNSYTV

. :::: *: * :: . . :*** * : . *

MTB3 IPVSLEVNFQLKARYFPQSSAFVIQLPLQLTRDNRFSQVTVEIVNEFNFFCKSQTNSTKF

MTB6 SMTTLEINFQLQSSYYPQQSAFMIYMPPQLSRDSRISNVSVDISNEFLNYCKIQTNSTQI

MTB7 --TILQIDFQLQINYLPYSSAFVISLPPQVIRDSSISNVSVDISNQFNFYCKSQTNQTQF

MTB2 SQQILEVNFLLSGNYIPQSSAFVFYLPPQLIRDSRISNVQVDILNQFNFYCQSQTNSTKI

MTB5 PPVALEINFQLQTNYYPQSSVFAIYLPPQLTRDLRFSSVTVDIENSFNYYCVSQTNSTQF

MTB4 PFVTLEVNFQIQINYFPQSSAFVIYLPPQIIRDNRVNNISIEIANQFNFYCSSQTNSTQF

*:::* :. * * .*.* : :* *: ** ...: ::* *.* :* ***.*::

MTB3 STSKVTYQNTVQQRDTLVIQCQFSGNQTINISSDVFTAKIQGYQLPMQVQKPTDRVIINL

MTB6 SNQTVNYQNKIQQREVISIICQQSGNTN---TQSVFTVTIQGYQLPRQLQKPTDRLIIDL

MTB7 FTQSVTFQNGIQKRDIISILCQFNGNMTVNNSLSIFTAKIQGYLPPYQVQKPTDRITIDL

MTB2 FSSSIQYDQNLQQRDTISVVCQFYGNTTFKDPQSIFTAKIQGYLLPKQVLKPTDRIIIDL

MTB5 SSQSIQFENQQQQRDSVSIICQFNGNDTLNSQQNLFTVRIQGYQLPEQLYKSTDRVIINL

MTB4 FSSNITFQNKIQQRDSISILCSFSGNITTNNPQYLFTAKIQGYLLPKEFQKPTDRIIITL

...: ::: *:*: : : *. ** . :**. **** * :. * ***: * *

MTB3 VDFSQNSFNEQYYCQSSENQNIPDSLKNIAVFVKTQSTNFIVSQYSYQIT-SYINQNKVS

MTB6 IDFSQKQNQPLPLCLTTENPDISNQVKSSWVFTNTKQTSFFVSQYDFKFNGS---SDQNT

MTB7 FDYSQKQYLSQSNCQTTENPLIPTYISNKWTFSNNQSTSFQFYNYSQQ-LNSTINQNQNQ

MTB2 IDFQQNSYQRTFQCQTSESLNNKPQLKNQWVFTNTQSSNFVQNLYFYQINTNSSNQSQAN

MTB5 FDYSQKQFNQQSLCQSSENPNIQLQIKDKWVFTNTQVTSFQFSQYSYEGN-----DSQNL

MTB4 IDYSQKQYQTQYQCQSTENLSISEQIKSQWVFNNNQSTDLSQNQYYYYQNSTTLNSNQSQ

.*:.*:. * ::*. :.. .* :.: :.: * ..:

MTB3 FTAQSLINITTNYSIQDGDLIEIEFGKQAFFKFEISSSSQQQISSVSEVKNLACSVIYPL

MTB6 LTLQSLLNMTTYYNISDGDLLFISFSKMNFFKIETLNGKLLPNQNSTENKDLNCSLVYPQ

MTB7 TSPYSIINITTNFNINDGDLIQLQFPKEQFLKSEATSEIYSSINASPEYKNLSCLTLFPQ

MTB2 TSVISIINITTSNQIQDGDDIVVQFPKQQFLKYESNQLAS--SQYFSELKNLTCIAIFPQ

MTB5 SSLKSQINITTSQNIQDGDQIVIQFDKQNFLKLENISLTQNN---IIQNESLNCSSIMPL

MTB4 NVPYSLINLTTRYPIQDGDYIVVSFSKLQFLKNEVINGIQ--ALAYPELKNLNCSFLQPQ

* :*:** *.*** : :.* * *:* * . : :.* * : *

MTB3 PAQPNSVSFIPTCVLVEKASSFSIQFQLYPSQLGQVLNWNNKDVVLNISGLAFQENLQQN

MTB6 SSAQ--SSALSQCILLETPSNYSIQITLKTANLNSISGWNSKDIFITINSLAFQQLLP-S

MTB7 SNGIASSSLLFSCTLSEGPSYYSINLGLRPSTAGVNSSWNNKDLVVNIIGIGFYPNFL-N

MTB2 ATL---QTNFISCILSEVSSYFSIQLTIQPNQL-SQIDWNKQDLVIQFTGVAFQPNLQ-N

MTB5 SDPFPSSNFISSCVLLGGPLSYSIQIVLYPSKLGQISSWNYRDLIISFTGFVFQPSTQ-C

MTB4 SSF---LNLFSTCNLSEGSSQYSINLQLYPTQLDSNLSWNYKDLALSVAGLQFFPNQQ-Y

. : * * :**:: : ** :*: : . .. *

MTB3 YQSEVLFSHSSSDLYLLSQSNQTFNNQLSVTANSVYQVSQISTLSS--LAQDYLNYKFDF

MTB6 NNTKIQFTHFSSDQYLLSQSNYLLNS-QQTTSNVFYSINQISSVTQQTITSFVQTIKFDF

MTB7 QYSLVQFSHFSSGQYLLSQSQFSYNNTQPIITNVFYQLNKVSSEQLFSLLYQNQNLKFDE

MTB2 ESSFIQFLHISNDNYLISQSNLSFTSNGQIITNIEYQVNKISFYQLQSLNDAIQYLKYDY

MTB5 FNCIIYFQHYQNSNQLVSQSNFTYFTNQQLLTNLSYSINKIDYLSQ--GINSASSLQFDF

MTB4 YNSSVQFQHISSNKYIISQSDYNFISENNVVAQAFYSFNKINSEQFQSLDSQSQRLTFDY

: * * .. ::***: . :: *...::. :*

MTB3 IQLQFSQSILVPQSNSNFQLTLNFSQQIYLSTLSYCSLDSLCSQRTQIVQCNLSSNSTSL

MTB6 IKIQFLMQTLMSTLDSGLNLAINFSQQVYFNVQTFCSLDNICSQSSHQIKCSVSSDGFTI

MTB7 IKLQYLSSAQIQPPNQPLTLIIVFSQSITFSNQTFCSINGFCSSKTSQIQCNISPDGFTL

MTB2 IQLQFIGQVQIQQLNQVLTLNLAFSQPIYFNELSFCSIETDCSKQKQIVNCSLSQDGILL

MTB5 IQIQYLTYLSLPSSNSSITLFLDFSQQLSFGPQSFCSIDSTCSQKTFITQCDLVSNGLTI

MTB4 IQLQFQTTLNLSSSSQNLSLTLQFSQPIYLSNLSFCSIDVGCSQSTLISTCSLSADGLLV

*::*: : .. : * : *** : : ::**:: **. . *.: :. :

MTB3 TLDNIGSWITCS-SVLSSFNITIHNPEINNQNSTGGAAQSIAINWNLTSTTSSQTLLSGH

MTB6 LLSQIDQNAFCSPPVLTNFNITIHNPEINNQNSTGGAAQSIAINWNLTSTTSSQTLLSGH

MTB7 LINSIEQYSFCGFSTLNNFNITIHNPEINNQNSTGGAAQSIAISWNLTSTTSSQTLLSGH

MTB2 LVQQIQLYATCNSSALSNFNITIHNPEINNQNSTGGAAQSIAINWNLTSTTSSQTLLSGH

MTB5 QINGIDKYVTCSTPNLTSFNITIHNPEINNQNSTGGAAQSIAISWNLTSTTSSQTLLSGH

MTB4 QINKLDQFSTCASSVLNNFNITIHNPEINNQNSTGGAAQSIAISWNLTSTTSSQTLLSGH

:. : * *..*************************.****************

MTB3 TSLTTNTSQCPQPHCATCTSPPSICIHCTQGYYLLPDQNSCVQTCPPPTVAHQQTATCQP

MTB6 TSLTTNTSQCPQPHCATCTSPPSICIHCTQGYYLLPDQNSCVQTCPPPTVAHQQTATCQP

MTB7 TSLTTNTSQCPQPHCATCTSPPSICIHCTQGYYLLPDQNSCVQTCPPPTVAHQQTATCQP

MTB2 TSLTTNTSQCPQPHCATCTSPPSICIHCTQGYYLLPDQNSCVQTCPPPTVAHQQTATCQP

MTB5 TSLTTNTSQCPQPHCATCTSPPSICIHCTQGYYLLPDQNSCVQTCPPPTVAHQQTATCQP

MTB4 TSLTTNTSQCPQPHCATCTSPPSICIHCTQGYYLLPDQNSCVQTCPPPTVAHQQTATCQP

************************************************************

MTB3 CFQHQECLQCQSQNPAACTSCSPTYSLNSTLLPYCYVPLPPSSSASSSVTKDVVNRTPSN

MTB6 CFQHQECLQCQSQNPAACTSCSPTYSLNSTLLPYCYVPLPPSSSASSSVTKDVVNRTPSN

MTB7 CFQHQECLQCQSQNPAACTSCSPTYSLNSTLLPYCYVPLPPSSSASSSVTKDVVNRTPSN

MTB2 CFQHQECLQCQSQNPAACTSCSPTYSLNSTLLPYCYVPLPPSSSASSSVTKDVVNRTPSN

MTB5 CFQHQECLQCQSQNPAACTSCSPTYSLNSTLLPYCYVPLPPSSSASSSVTKDVVNRTPSN

MTB4 CFQHQECLQCQSQNPAACTSCSPTYSLNSTLLPYCYVPLPPSSSASSSVTKDVVNRTPSN

************************************************************

MTB3 STFSGALNRPEPGQPSQKQQQQQQQQQQQEQQ-QQQQQQQEAQASDQRGFAHFLAQTKSY

MTB6 STFSGALNRPEPGQPSQKQQQQQQQQQQQEQQQQQEQQQQEAQASDQRGFAHFLAQTKSY

MTB7 STFSGALNRPEPGQPSQKQQQQQQQQQQQEQQQQQEQQQQEAQASDQRGFAHFLAQTKSY

MTB2 STFSGALNRPEPGQPSQKQQQQQQQQQQQEQQ-QQQQQQQEAQASDQRGFAHFLAQTKSY

MTB5 STFSGALNRPEPGQPSQKQQQQQQQQQQQEQQQQQEQQQQEAQASDQRGFAHFLAQTKSY

MTB4 STFSGALNRPEPGQPSQKQQQQQQQQQQQEQQQQQEQQQQEAQASDQRGFAHFLAQTKSY

******************************** **:************************

MTB3 TKGFILTLLIPLSILGACLTRLVTFCLKKREKKVHPPLPSESRSAQIAQNLDERQETQKD

MTB6 TKGFILTLLIPLSILGACLTRLVTFCLKKREKKVHPPLPSESRSAQIAQNLDERQETQKD

MTB7 TKGFILTLLIPLSILGACLTRLVTFCLKKREKKVHPPLPSESRSAQIAQNLDERQETQKD

MTB2 TKGFILTLLIPLSILGACLTRLVTFCLKKREKKVHPPLPSESRSAQIAQNLDERQETQKD

MTB5 TKGFILTLLIPLSILGACLTRLVTFCLKKREKKVHPPLPSESRSAQIAQNLDERQETQKD

MTB4 TKGFILTLLIPLSILGACLTRLVTFCLKKREKKVHPPLPSESRSAQIAQNLDERQETQKD

************************************************************

MTB3 GNGGDEEQMRASSRVDTANMCPLNSRRGEQLQLEGVQQQSDGGVGGGESEGNGYLAFCWI

MTB6 GNGGDEEQMRASSRVDTANMCPLNSRRGEQLQLEGVQQQSDGGVGGGESEGNGYLAFCWI

MTB7 GNGGDEEQMRASSRVDTANMCPLNSRRGEQLQLEGVQQQSDGGVGGGESEGNGYLAFCWI

MTB2 GNGGDEEQMRASSRVDTANMCPLNSRRGEQLQLEGVQQQSDGGVGGGESEGNGYLAFCWI

MTB5 GNGGDEEQMRASSRVDTANMCPLNSRRGEQLQLEGVQQQSDGGVGGGESEGNWYLAFCWI

MTB4 GNGGDEEQMRASSRVDTANMCPLNSRRGEQLQLEGVQQQSDGGVGGGESEGNGYLAFCWI

**************************************************** *******

MTB3 AVILLLGNVGDLVEVPYIIFSQQNSFSNKSTTNVFDLQFSEADMGQICCLSYIALNAVCY

MTB6 AVILLLGNVGDLVEVPYIIFSQQNSFSNKSTTNVFDLQFSEADMGQICCLSYIALNAVCY

MTB7 AVILLLGNVGDLVEVPYIIFSQQNSFSNKSTTNVFDLQFSEADMGQICCLSYIALNAVCY

MTB2 AVILLLGNVGDLVEVPYIIFSQQNSFSNKSTTNVFDLQFSEADMGQICCLSYIALNAVCY

MTB5 AVILLLGNVGDLVEVPYIIFSQQNSFSNKSTTNVFDLQFSEADMGQICCLSYIALNAVCY

MTB4 AVILLLGNVGDLVEVPYIIFSQQNSFSNKSTTNVFDLQFSEADMGQICCLSYIALNAVCY

************************************************************

MTB3 LICIVMMVKAIIFETGSGEPLFCIYEVKLSSSSSFCGEEMKKDKSEAAACKGDSNAQNEN

MTB6 LICVVMMVKAIIFETGSGEPLFCIYEVKLSSSSSFCGEEMKKDKSEAAACKGDSNAQNEN

MTB7 LICIVMMVKAIIFETGSGEPLFCIYEVKLSSSSSFCGEEMKKDKSEAAACKGDSNAQNEN

MTB2 LICIVMMVKAIIFETGSGEPLFCIYEVKLSSSSSFCGEEMKKDKSEAAACKGDSNAQNEN

MTB5 LICVVMMVKAIIFETGSGEPLFCIYEVKLSSSSSFCGEEMKKDKSEAAACKGDSNAQNEN

MTB4 LICVVMMVKAIIFETGSGEPLFCIYEVKLSSSSSFCGEEMKKDKSEAAACKGDSNAQNEN

***:********************************************************

MTB3 GEKHKKVLSGRKLWKLIIDVFLRCLVVVGGKAFCMVYSNVANVKGWLTCQVDKNLRAFRL

MTB6 GEKHKKVLSGRKLWKLIIDVFLRCLVVVGGKAFCMVYSNVANVKGWLTCQVDKNLRAFRL

MTB7 GEKHKKVLSGRKLWKLIIDVFLRCLVVVGGKAFCMVYSNVANVKGWLTCQVDKNLRAFRL

MTB2 GEKHKKVLSGRKLWKLIIDVFLRCLVVVGGKAFCMVYSNVANVKGWLTCQVDKNLRAFRL

MTB5 GEKHKKVLSGRKLWKLIIDVFLRCLVVVGGKAFCMVYSNVANVKGWLTCQVDKNLRAFRL

MTB4 GEKHKKVLSGRKLWKLIIDVFLRCLVVVGGKAFCMVYSNVANVKGWLTCQVDKNLRAFRL

************************************************************

MTB3 FYMTLCIHAIFNMISAVFFTLMLTHFSFASWTAVAQDSLTSQGSDGGVEFSFFVDILAFK

MTB6 FYMTLCIHAIFNMISAVFFTLMLTHFSFASWTAVAQDSLTSQGSDGGVEFSFFVDILAFK

MTB7 FYMTLCIHAIFNMISAVFFTLMLTHFSFASWTAVAQDSLTSQGSDGGVEFSFFVDILAFK

MTB2 FYMTLCIHAIFNMISAVFFTLMLTHFSFASWTAVAQDSLTSQGSDGGVEFSFFVDILAFK

MTB5 FYMTLCIHAIFNMISAVFFTLMLTHFSFASWTAVAQDSLTSQGSDGGVEFSFFVDILAFK

MTB4 FYMTLCIHAIFNMISAVFFTLMLTHFSFASWTAVAQDSLTSQGSDGGVEFSFFVDILAFK

************************************************************

MTB3 FLMSLICFLNCLHIQQLISACKSPNLPGPQNPVSHRLQSPSTPSSSASPADAVERDACKV

MTB6 FLMSLICFLNCLHIQQLISACKSPNLPGPQNPVSHRLQSPSTPSSSASPADAVERDACKV

MTB7 FLMSLICFLNCLHIQQLISACKSPNLPGPQNPVSHRLQSPSTPSSSASPADAVERDACKV

MTB2 FLMSLICFLNCLHIQQLISACKSPNLPGPQNPVSHRLQSPSTPSSSASPADAVERDACKV

MTB5 FLMSLICFLNCLHIQQLISACKSPNLPGPQNPVSHRLQSPSTPSSSASPADAVERDACKV

MTB4 FLMSLICFLNCLHIQQLISACKSPNLPGPQNPVSHRLQSPSTPSSSASPADAVERDACKV

************************************************************

MTB3 SYFENTPNAAEKTTPTAVTLASYRQQETSGTGSQNLIEGRPRRKKPSKLSLLLKRDSGQK

MTB6 SYFENTPNAAEKTTPTAVTLASYRQQETSGTGSQNLIEGRPRRKKPSKLSLLLKRDSGQK

MTB7 SYFENTPNAAEKTTPTAVTLASYRQQETSGTGSQNLIEGRPRRKKPSKLSLLLKRDSGQK

MTB2 SYFENTPNAAEKTTPTAVTLASYRQQETSGTGSQNLIEGRPRRKKPSKLSLLLKRDSGQK

MTB5 SYFENTPNAAEKTTPTAVTLASYRQQETSGTGSQNLIEGRPRRKKPSKLSLLLKRDSGQK

MTB4 SYFENTPNAAEKTTPTAVTLASYRQQETSGTGSQNLIEGRPRRKKPSKLSLLLKRDSGQK

************************************************************

MTB3 SGSSLSSRQQETPSPNLPSYSPNLYPSQAYI

MTB6 SGSSLSSRQQETPSPNLPSYSPNLYPSQAYI

MTB7 SGSSLSSRQQETPSPNLPSYSPNLYPSQAYI

MTB2 SGSSLSSRQQETPSPNLPSYSPNLYPSQAYI

MTB5 SGSSLSSRQQETPSPNLPSYSPNLYPSQACI

MTB4 SGSSLSSRQQETPSPNLPSYSPNLYPSQAYI

***************************** *
