# Supplementary material for: Selecting One of Several Mating Types through Gene Segment Joining and Deletion in Tetrahymena thermophila
Source: PLoS Biol. 2013 Mar 26;11(3):e1001518. doi: 10.1371/journal.pbio.1001518 (PMC3608545; doi:10.1371/journal.pbio.1001518)
Supplement: Text S3 — Multiple sequence alignment of the furin-like repeats in the TM regions of the predicted MTA and MTB proteins. Clustal Omega was used for the alignment. Key: aqua, cysteine residues; yellow, other amino acids shared by every furin repeat-like domain. Symbols in the bottom row are as in Text S2. (DOC) [file pbio.1001518.s014.doc]

**Text S3. Multiple sequence alignment of the furin-like repeats in the TM regions of the predicted MTA and MTB proteins.**

Tthe_MTA2_962-1049 ----PNCQACASNYAACTACAPGYLKSQYNHHAVLACLPTCSPQYVAYNG--TCLACQLK

Tthe_MTA3_1011-1098 ----PNCQACASNYAACTACAPGYLKSQYNHHAVLACLPTCSPQYVAYNG--TCLACQLK

Tthe_MTA4_981-1068 ----PNCQACASNYAACTACAPGYLKSQYNHHAVLACLPTCSPQYVAYNG--TCLACQLK

Tthe_MTA5_1012-1099 ----PNCQACASNYAACTACAPGYLKSQYNHHAVLACLPTCSPQYVAYNG--TCLACQLK

Tthe_MTA6_1014-1101 ----PNCQACASNYAACTACAPGYLKSQYNHHAVLACLPTCSPQYVAYNG--TCLACQLK

Tthe_MTA7_1033-1120 ----PNCQACASNYAACTACAPGYLKSQYNHHAVLACLPTCSPQYVAYNG--TCLACQLK

Tthe_MTB2_1054-1132 QCPQPHCATCTSPPSICIHCTQGYYLLPDQN----SCVQTCPPPTVAHQQTATCQPC-FQ

Tthe_MTB3_1055-1133 QCPQPHCATCTSPPSICIHCTQGYYLLPDQN----SCVQTCPPPTVAHQQTATCQPC-FQ

Tthe_MTB4_1067-1145 QCPQPHCATCTSPPSICIHCTQGYYLLPDQN----SCVQTCPPPTVAHQQTATCQPC-FQ

Tthe_MTB5_1062-1140 QCPQPHCATCTSPPSICIHCTQGYYLLPDQN----SCVQTCPPPTVAHQQTATCQPC-FQ

Tthe_MTB6_1057-1135 QCPQPHCATCTSPPSICIHCTQGYYLLPDQN----SCVQTCPPPTVAHQQTATCQPC-FQ

Tthe_MTB7_1051-1129 QCPQPHCATCTSPPSICIHCTQGYYLLPDQN----SCVQTCPPPTVAHQQTATCQPC-FQ

*.* :*:* : * *: ** :. :*: ** * **:: ** * ::

Tthe_MTA2_962-1049 DPQCLSCSPSNLTQCSSCNQGYTLVPEFNGCVDS

Tthe_MTA3_1011-1098 DPQCLSCSPSNLTQCSSCNQGYTLVPEFNGCVDS

Tthe_MTA4_981-1068 DPQCLSCSPSNLTQCSSCNQGYTLVPEFNGCVDS

Tthe_MTA5_1012-1099 DPQCLSCSPSNLTQCSSCNQGYTLVPEFNGCVDS

Tthe_MTA6_1014-1101 DPQCLSCSPSNLTQCSSCNQGYTLVPEFNGCVDS

Tthe_MTA7_1033-1120 DPQCLSCSPSNLTQCSSCNQGYTLVPEFNGCVDS

Tthe_MTB2_1054-1132 HQECLQCQSQNPAACTSCSPTYSL----------

Tthe_MTB3_1055-1133 HQECLQCQSQNPAACTSCSPTYSL----------

Tthe_MTB4_1067-1145 HQECLQCQSQNPAACTSCSPTYSL----------

Tthe_MTB5_1062-1140 HQECLQCQSQNPAACTSCSPTYSL----------

Tthe_MTB6_1057-1135 HQECLQCQSQNPAACTSCSPTYSL----------

Tthe_MTB7_1051-1129 HQECLQCQSQNPAACTSCSPTYSL----------

. :**.*. .* : *:**. *:*
