# Supplementary material for: Selecting One of Several Mating Types through Gene Segment Joining and Deletion in Tetrahymena thermophila
Source: PLoS Biol. 2013 Mar 26;11(3):e1001518. doi: 10.1371/journal.pbio.1001518 (PMC3608545; doi:10.1371/journal.pbio.1001518)
Supplement: Text S4 — Predicted amino acid sequences of mating type genes from T. malaccensis, elliotti, borealis , and pyriformis . (DOC) [file pbio.1001518.s015.doc]

**Text S4. Predicted amino acid sequences of mating type genes from *Tetrahymena malaccensis, elliotti, borealis* and *pyriformis*.**

>T.malaccensis predicted MTA

MIQAPDQGLTYCQIQTNDEASILNQPKNQNYFNDKVNTVNQLAFWSKNLSKTFLPFPQNAFERNITLNFYDANMSFLGPSYIYSKVMIKKVLQNYIQDASNNKMIQITLQTYGQFQIIDKTNFYDFFQVKFYTNSILQSSPQNIFSLQADGQVYIYVMINSYSSYTELFITQLIYVKKFNQSDFPDKFQNLQFTFSEQTFQSINIQILFGSTNIKVTDYNQYYSFVQLQNSNFSAVIRPSLAFSQIQNEIAFNITDIVLSQCLLQSILLPSGLQNQFMKSEPNFFVFNPTNNTYQNYGTNYLNGNASLQKSQGFQSTKGSNQIQMLISSFSDVVGQNSQLTLGFNCQNGIKTLKNLSISSNELQNANQTQVFILFNNSQAKSLSIQIQIPDIKVSNTNLFIQLPQGIDYQQKNQSSLSVQGFAFSSFQWDQNIVSFYSTNFSSSSIAVSIQQVQLQSSSNNIQNVTKIIAKCILNQAFVFYVDSNSNQTIQIIQPQPLPTTSIQINTFNQTQFTSFTSPENRASLQSQLAFSFSILNFQDTCSWLIVSLPSSFTIGFLQNSQFNLFDCFGNTYSYTQGNPISSQSTIGYTDNNHCIYISCKVLRSVSSSHAATSCLNNTVTIQNVRSPDFPLQTAGLQFLIANSNTSSQPSSDPPTFFNQSIDLTDSSLPYFFIGSEVFTHEGLNITQADLSSLSTQISSNYFGDVFNFSMTFAYPIYFWEQHQIDVQLPFRLVGKQGIECWPMYAVVCSVKGIGELQSQSQWTTFRIQFVNLTAPNTVITLQISYGALNKNIQLGQQLFLGISVNLQNRIINYQNKSIDAFNNLTVQSLVTLQKEQPLQISNPLIGMLGVNYTFNVQQFQIPQKVDQNYYLSLNIDSSFQLNSSQISFYQLTQSQNSSGTNNLIEHSIEFIYNSTNSFLIPIISLVPTYPFQLRISGLRNPSGIVEQNEQNVAQRYNFQLIWSTQQGNSLKNVWVIQSVSLPISSKYTCSPNCQACASNYAACTACAPGYLKSQLQQQQQYNDHAAVLACLPTCSPQYVAYNGTCLACQLKDPQCLSCSPSNLTQCSSCNQGYTLVPEFNGCVDSHLLQTPRSRLLDYSLSTNLPDNIDTHPDDDNDDEDDEEQEEDDDEADEEEEDEADADDDDDEEDEEEDAQKSDKAQSLTRMTQESSSAKADQREGGESSSNTGSKVMGQLRDTVGALKGGGAIFIWIVLAALAVSVSQSVLRYTYNKLKCKSPSNGRSSSGSSSNSSSTKNNNNKNNNIRRSSMRRSSSSRSSSGSSSGARNGSWKEHKGVSERAEELHGQRIDSLMLFLLSIAEAIQMPYTILWGFSVTGGDFESPVLQTLLGACALSTLLWIFDAYLLGSILANSENTPKTSCLLNPLPFSKRKGSLSFVIVPARLAFSLIPKSVSLTLTNIFAVEGWFRYPYGEDVGRATIVLTNFRKVLSSQIKSNVSGLCSLVSFLILQYPEVLTSYVQIYDLIIFDVVMAILCLTSIRNVDKLISILNQIQENEGELWTNAK

>T.malaccensis predicted MTB

MNTTYKIAFLLIFASLLIDRSQEALICYPINPIQSMKQIYQLPYNYRLGSDPVPSQYNFALQFDQLSPDLLYTLSSIAYLKRYLITSNNQVISIIPNSIGDSLNYIPLELTQYTFSIVAIFSSLSSFSDNQDFLTSYCSITQFPQTDLTIFRQINKQNQQFLISTMLISQIQDLNSLVLSYPTSFAIKSQNAANLGCQLYTDYGIINDCSVSLNAQSGLSFVTFNLTNFNFSLLNVSATIVLNYSTFNQNSLSNKQFSLQLLNQYQSQIAQYSSFQIIDQRICQQTTFSASLINNVPSFGQNLKLKINFVPASANITRIIFEFSSQIYTKIDLSFPYLTILDSTQTNQQKVRCLKAYQFMLVCDLIGGNLLFDVTKGVNIEFPYVQLNVTSYSQTLSFQIKYFTDLTFQTCFVSNFTQKFQAVSPSSPQAFLYSNNSLQIRLFDLIMLDNSSLINISLPSQLAFTSTSKLSQIAGISNLSQTQQKTQQNIQISQIGLSLAQLKTYQGISFQLTNNTIYCSLSSLNQIQIEIVNSLGVIVLSGSIPLQNEPYPIQIQQINPIFTPLNQQQQLQYQQQQNSYSIPFVTLEINFQIQINYFPQSSAFVIYLPPQIIRDNRVNNISVEISNQFNFYCSSQTNSTQFFSSNVTYQNSIQQRDSISILCQFSENITTNNPQYLFTARIQGYLLPKQVQKPTDRIIINLLDYSQKQYQPQYQCLSTESSSTSEQIKSQWVFNNNQGTDLLQTYYSTLSISNQNQSIPYSFVNLTTRYPIQDGDTIIIRFSKLQFFKNEVINGNQQQLIAFPELKNLNCSFLQPQSNLSTFPNLFSICNLSESSTWYSINLQLYPTQLASNLSWNYKDLALSLVGLQFYPNQLDQNQTVQFQHISSDNFLISQSNYNFTIKNNIASNALFALNKIKSGQFQSLDQQASKLLFDYIQLQFQSSIFLLPSIQNITLTLQFSQEIQLSDDSFCSIDIGCSQRTLILKCNLSLDGLYIQIDQLGQYSTCTSSVLNNFNITIHNPEINNSNSTGGAQSIAINWNLTSTTTSQTLLSGHTSLTTNTSQCPQPHCATCSSLPSICLHCTQGYYLLPDQTSCVQTCPPPTVPHQQTPTCQPCFQQQECLQCQSQNPAACTSCSPNYSLNSTLLPYCYVPLPPSSSGPSSSSTSSSPSSSPSSPSSSSSSSSSVTKDVVNRTSSNSTFSGELNRLERGGQQQQQSQKQQEAQASDEGGFASFLAQTKSYTKGLILTLLIPLSILGACLTRLVTFCLKKREKKVHAQPSESRSAQIAQNLDERQETQKDGNGGDEEQMRASSRVDTGNMCPLNSRRGEQLQLGSVEQQSDGAGGGEREGNGHLAFCWVAVILLLGNLGDLVEVPYIMFSQQSSFSKKSTSVFELQFWEADMGQICCLSYIALNGICYLICVVLMVKAIIFESGSGEPLFSIYEVRLSSRSSFCSVGGEEMKKDKSEVEWKGDSNRQNENKEKHKKGVSGRKLWKLTIDVLLRCLVVVGGKAFCMVYSNVANVKGWLTCQVNKNLRAFRLFYMILCIHAIFNMISAAFFTFVLTHFSFVSWAAVVGQDSLTSQGIDGRVEFSFFVDILAFKFLMSLICFLNCMHIQQLIAACKSSNLPGHQHSTSHPVQSPSTPSSSPSPAGAVARGVGKASYFENTPNAEKTTPTTVTLASYRQQDASGTGSQNLIEGRPRRKKPSKLSLLLKGDSGQKPGGSSSSRQQETPSPNLPSYSPNLYPSQAYI

>T.elliotti predicted MTA

MMIVFLFVISTLLKVNIQARSIDQGIITEVKPQEYKYLRITTNDESAITRFDGKIELFNSKIYKTNKLLFDKPQENFYFLPLTQNTFGRLVSQDFLDESMNVLNAGISNIQSQVIIKKQNQKLIDGNNGKKWLSIIFNTYQQFKVMSNQINSRFDINVLIPYIKSSQPSIDIFSLLIDGKKINSFLNQTNNAVKDATLFSQSFNLADFPLQFNDLEIQFLDPFFPSLSITLSSGADYQQIQIVNQFYSAYQLYNSDYSAIILPQIENPIISQLVFSLKSNIFNQCLVKQIGLPQEFISQFPLTNSLVLQTQNSESSSQIFAIDFFNFINGTIFFPQNQTFTGLPSQKPTSNAITNIIISSFLDLNGLDKNLTMALFCSETETANLSVEIVKNEYQFANQTQAAILINNSGTKSIKIEISIPSINIQKASLFLQLPRTIICQNTTQESFTVEGFTYSSFVWQNYQIVFNSVSFTSTTLRLTFDNIEIKSKTTEADIFNNSKIIAKCIFNQTFVFYVDSRTTQTVQIIQPPPLQLTQMQINSFNQTKADSLNTLENRTSVQSQLAFSFSILDFQSTCQWVIIQLPLEFTIGFMKMSQFNLQDCSGSQYSYLQGSTQSSLSTIIYTDNNSCIYISCQSLRKASQHNSKTQCLDNIVTISSVKSPDLAYLTLPLKMFIANQNSTINDDAGLPTFFNEDLNLQNQSLPYYFIPNEIFTYPGINITEVDLSTVYFNITSNFYRDVFNFSMVFAYPIYLHDQHQINIKIPIKIMGNQNIQCQPSSFLYCSLKIDTSENSQTIVQIKFLQQIMPDTKIYFSLDQVVANNDPLQNQQSFAFVQLVLSNRIVNTKNITIDILNGIKYYKFISLDETSPLQISNSYLNFANVYYTFNIKSLEIPPEEQNNYYLTLKMDSSIQFNSSSSSCYILTQTTNKESPDSSFNELNLNCQVDSQNNFLIPVSQILFSEKFQLRISGLRNPSGILEQNEQNLAQTYNFSLIWSREQANRVRNVWLIQSVSLPITYKYTCSHNCQGCASNYAACTTCAAGYLKSHHNDHTVLACVATCRPFHVAYNGSCVACQLKDPYCLSCSPSNLTECSSCNQGYTLMPEFNQCLDSRLLKTGRSRLLDDSLSTNLAHNIDTHPDDDNDDDEDEEDYAATTAADDNDAHKSHKAQSFTRMTQESSSAKADQTEGGESSNSGSKVMGQLQDTVGALKGGGAIFIWLVVAAFAVSVSQSVLRYIYNKLKCKTHSKGSGSSDHSTMRRNSNSQSGEVSGFEARNKSGKDHKRVSEKEKELSGQRIDCLMLFLLSLAEVCQMPYTLIWALSVTGGDFESPVLQTLLGACALSTLLWIFDAYQLGSILANTESTPKTSSLLNPLPSSRRTGSLSFVIVPTRLIFCLIPKSLSLTLTNIFPLEGWFRYPFAEDFEGAAILLTNFRKVLSSQIKSNITSLCSFISFLILQYPEVLTSYVQLYDLIIFNFVMTVLCLTNTRNLDKIISILNEIQDNE

>T.elliotti predicted MTB

MKFSPLNNQKVAFYFMILHLVRKLAEGKGSCFLTSSSTQTWQQIQPVAYSQREGFTNFGPQYLFTVQLSTNNTDALLGTESTQLTQFFLTSNQFMLSKIVSKVAGFQQPPQLQKTTFYIAAAFTSQQVSSGLVDFGSVCDMSTYPQLALREFVLVNSQTQQLFIQVPIISQINDIYYLNLQYPTSLALHTQDNNNIGCQLYTDYIVINECQLISSSGYTTVSFDLSKMIARNISATVVLSSGTFNQNLINQNKPFRVTLLNQLEYLIAQSPNFNAINYNQCSQTNFSAQVLYNSSYQINEQRLQVKFTPVSSTISRILLEFSTPIIPKFSLTSITVIMSDYSLQNSFTVLCQKVYDSIILCDGVFTSVPTFDISKGLILNLPYFEARQAVPPSFHTFTLKYFPDYSFQTCFYTNSTIPFMQQKVQTPFAYLFNNNTLQVSFSNVINLENATIIDIQLPTKLSFPSTAEINQISGISILSQISKSSASNAKITQVTQSLEQLTTFKGISFSLQNISPNSIQCNPTNPEVLNIQIINSFGVVILSGSILIIIEPYSIQIQSKKINQLPDTQQQQQIQQNGNFTDPVSLEINFQLNADYFPQQSAFVIYVPPQLIRDTRFQTVTVQIANQFDFYCKSQTNSTKFLDLAVPFSNTIQQRDTIVVLCQFSGNQTISSSSDVFTAKIQGYKLDYQVEKPTDRIIVDLFDYSQNQFQKQYLCQTTESKNNPYSMKNQWVFVKTNASNFIISKYLYQVSTAIYQDNGVKPIYSLINITTSYCIQDGDTIQINFEKQQFLKFETTPTSQSQIPSISVVKNLVCSTIYPLSSQPNSNNFITNCILVEKASSFSVQIQLKSSEIGQLSDWNNKDVVFKVTGLAFQENFQQSSQAEVLFQHFSNDQYLVSQSTQKFANQQPMTVNLEYQINQASTFQTLTQNNQKISFDFIQLIFLQSILLPNSNQNVQLNLSFSQQINLSSSSFCSINSFCSQLTQTVQCNISQNSMSITINSIDTYSKCRSELNAFNITIHNPEINYSNSGGGGQPIRINWSLTSSTTSQTLLQGDTSLTTNNTFQCPQPHCTACTSLPQICIQCTQGYYLLPHQNSCVDTCPPPTVAHPQTATCKPCLQEQECLQCQSQDQAACTSCAPTYSLNSTALPYCYVPLPSGSSSPISKGVATRKSSDSRFSGGYNRLEQGQLSQKQQVQASDEGGFVDLLGEMKHFTKGFILTLLIPLSILGAWLTRLLSFCLKKGGKKVSAVPSASRNGQVGVENLDERQETQKDVNGGDEEQMRPSSRLDTANGNMYPLNSRGEQLLLQSVQSQGDGAVQSEGTAPLAFCWIAVILLFSNLGDLVEVPYIIFSQQNSSSKQSINIFDLSFSEADMGQIFCLSYIVLNAVCYLICVVMMVKAILFENGSDSPLFSIYKVSLSNSSSYFSAGLIKKEKIEVEGKGNLSSLNQKTNKQKVNNEPKLWQLILDILVRCLMVLCGKAFCMVYSNMGNVKGWLTCQVNINVRIFRQFYIILCIHALFNMAATAFFTLMLTHLSFASITADAAGAAQDSLTTQSSDGNVEFSFFTDVLTFKFLMSLICFFNCLHIQQLINSCKSLNLPIKERAAFQPVQNPSSPSSSSSPAAAAPGTATMGKASSFENTPNTEKMTPTAVTLSSYRQQETPGTNYQSQFEGKARKKNSSKLSILLKRDPSQKSGNSLSSRNQETPSPNLPTYSPNLYPSQAYI

>T.borealis predicted MTA

MNIPQNSIGQILYVSLLDKNNQTIRKQQMIQSIVSDQKQNEEFQIFIQSKEDDNTYAGVLVQSIPNKIFKKQCNDCNNITIKLEVFYYPYLNGTNNIIPYENEIDTKFDGENIVLKKYTKEIIKTSTYTNATYQLVIDYQKIPDSFKIIQFSLRNYQFEKINYYFQSFGISLKKIINSNNIGPYISTNIVQNALILPYYSYNPTWNDLINAHASIIQIDFYLQPQWSCRLIQLTIPQIFQKQINGNDQVYFYYKLDSDNQLRGNIVKYNNITGAYTFRDFSFSFENQYRYLILSSFSSPYLEELSIELQMKCDNFDPLIIKNISISQNTKDKIGSSINQISMIFDDRDEKYFELQFSIKNITNIDSVLLLQMPLQLYFTDNDINNVDIFGFIFSSYKWDQNNILFENTTFNSNQIHLKFKQVKLKQNKNIKWIINIRCFNKRQFIFYTTESTVQDLQILETKIDLPTDLTTLLVKTFQLRKPFPNDQILENRTLVPSSLFIQFDVTRFTDSCAWIIFTVPQDIVIGRIENPLVSFQDCQNQQHSYQSSNDDQSKQVILINKNSNNIFFSCKYLKSIATLTQIGSEQILQCLNNTVSIQHTENPQEQKLTNNLTLYFANLKTPDQTDDSPPIQFNPQTYNQQQNYAYQAYETQSFTGIYIEQEFEPIHLSLQAISNFTYDVTTYNLSFILPIYLHKEDYQVKVKLPIKYLGSNPTKDILCGPTPDYEGQLASQVVNQISIEINVHSLLQPGFLFNCVFANSARINQTDLSINQLEEDSSLNMLFIQVYQEGKLLSEIQKNLNMSGSQPLSEWIYIAKYDPLTLSSYYMGQIGAEYTFSFDQIILPESNDQQQRFIEIQIDNSLMISEQTKCFLIIFSKDIPENLKEFKLEVNCQIIQPNILLLPSQALFSIKQPFKIQLSSIRNPIQDIGSSNKINLKLKFQFSYIWQRSLGIEEQTHYITQTQFSIDIFFNCNSSCQGCTQNYQNCIKCSSEFKLQQIKGNRKECVKKCDQLQAIIGNECTNCQVLEQNCSTCSSENLKQCNGCSEGYIYIQKWQSCVDQSLFPRRILITQNYENEEKIENTKSDVLQYEPNKNHDNFRLIQENSSSDKDQVINKEQNQNSQLLNKFQESLEGLKSGKIIFLYFICSAAAFSIFLNTLQEIFKKWKVSHQYVNNNSDQNKSKNPTQNQNIDQFNNHNIQIKEILEQIYSYRYTSLMLFILSVTEIIQIPYILFWGLAISDSNLAHPVMQTLIGTCALSGISWIFDSYQLGFILANKNNTPKNSCIFNPLPLSKREGLLSFTIIITRIIFSLVPKSVCITLSNAFNINGWFCYPYFEDQGRSSVLLTNLRKILGQQIKCNIAGITAFITLLNLQYPEIMESFTLIYDIIIFNIIMAILCFFNIRTIDSILLKLHELTENS

>T.borealis predicted MTB

MRRKQILLNLFIILLIFKFAKQFQLNYGFLKISKDLKLIEIRKDTQLSKGVLINSAYSYQWYIYFSISEFTEDDDLSYLYSSLSLCLDSQSLGQASIDSFTVEQDTSIIDYKNQGIEKCFNGSYDSPVTLGQNFEILITFQVSLTSTDDITDKITLTTNLSQTKFQLLLIGITESRFNLIEHKLFIYGEFFSLGSIPSQIIVRFPKVILNSNQDLTKLNVQILNQNSGTIHGLQTYSDEETISITFLMDEWKFFSDFFSFALIFQEDNFKTDLQTLGNFQIDSIDEYGSLVSQSDQIQIKLQKCSQSLQIIQSTQSIIIGQLSEYGITVQIDNLQLNNYRLVLQTAKNISIMGELNKAYFTFESIDKSLKKYWICSQFSSILLTICDSSQFISQNINGQVKFSIKNIFLKSDELKPYQMKLILENKSDSSCYSTDILQIQGDPHPTNEITIQYSEYNSRKLTIQFLTEIDLLDISIITLEIPSNLQLNSLTLYDTINLNKQANLLEISPNTIQISNFTESKVLTKLNWIRFSFKNAVASSALYKLQANNNFKLTISTNGVEDQTQGVINLQINPDPLFIEKSYLALEIPNENIMSYSVALTINFKILFENFPRINEDYQNLVSPSEFHILLPPQLEIDLTKSLISIKFEGAFNEFCYEHNQFKIEKINDPLNKENTTNRYVVIIFCTQPALFYQKSSNSYNLLIQGVIMPKYAQQTDRLIIQMKESNQNNTNQKIKLYFTSEHEIFDIEELNKWIFQNEIQLEFKHLFYKYQQDFNCSSYDEAIMKNNKIDFIIENNIQFYEGSVLKIVFDKQNFQLFENDQSKIFNNLLTYQQKLLICSFQSKQGVSLNQIIQVCMLQETEESFSIKIQIFPSSSTEILEWDRKYISVQINEIAFIPIIRTQTNQITFTLSTMNDEQINQSIYQIQNEQYFQLTILDYLQNNQSIEEQNNDYYFDYVLFLFESFAQIENLNLILAFNKNFNMNQDSQCLIEICGIKNIQINFILNLNNQSITIPNVKEQLQPYIPQNFIMQNLKLKITRISIDLITSNSQLENLEIKWSVSSLINGSILTKIQSLPIQLKCQQEECQSCVNNGYNCIECKQGYHLFVEEKKCVLQCPSHTVLDQATNSCRICLTQQQNCISCHPKLLKTCSQCAQNYFLSLTNPSYCYLPPSSSPLKSLLPSSLLNKTSEVSSSLSSTNEESTVSSSSNQLNKEDQIKEQKQSQDQNEKQKRGIGGLFGEIKTQARGLFLTLTIPISLIAATITKLINSCLRKNEGNKNVSIQIGQSSPEKCGQLQSLQQQHQQNKSLIKYQRREYGHFSWIATLLFFMNLGDIIEIPYILFMIMSETQSIPFTLSLFQAEQIPILVYLVVSFFCYLYCFLIIIKPMVFDQTALSIFYIFSINNNRNSINNRSYNKNHTQTQDQIQKEDFDQKLGMAQNKNGQNKKSECTLMLCAPKQIINFITRCFIAFLGKAFCMIYTNAGDVSGWFFIQVKEQLKLFRAFHHILCIHTMFNILSGILITTILTQFPFTSFSIYQQKEIQIQYSFLLDILIFKIIMSFICYANCLHIQALIQTTLSPISLEQQTPITSTVTANSFTLPQSGIYSTYHQVSQLPSELKCQEQQNKIQQQYYQGSHSPYQTSCPSPITTSM

>T.pyriformis predicted MTA

MTDLKYGIILVSHKIQQRFEKIPQLTYLANSISIDVIIDQKKITRKEIKTKAKAKAKPLPHYFLSKQILRECSKQNSLSISLLLITSLKKQFLIGSLLTCIALSTNFTSSCQMVGNSQSCAVGYQQIKLASYLVIQSNDDLANYPTTSYIYNELYTYMRLNCSNINSNSYRLDLSQFNQTYFNLSIYLTYHLLLSIPVNNFGRSLYAILYNSTGFEQNRYALTPVITNQFNQSQIKLQNGSVNYFKFYYKSQNNFLFTPQISNQSLTFLITLNTLPVKSMNNIQNVNDLLADINFLIDDQHYIQVMSTQFPSTFSQISLQFKYPVYTQIQLLLQGQQFTWKITQNLYFSSSYFLRENNYTASILPYFPSRTNYSNSLISNGSSYLQIQLSRSNLFLCDINKIILPQNYQQEIINGSPLLVQTLDSATNLTVNQNIAFNSSTNELTFQSLGYSLSQGNGTFFISIFSFNSFYVTENFLSISFSCILEKYTQTIPIQQKRQQNLREVQVIYDERSLNNIQILFSYTPFVASNSAFFYLSLPSNIYYQQQNKTAIITQGFSYTELQWVKDEVLFMNCLFDPSQQINITIFQVNITSDASIGNPNIKSFSQDQYYFYTDDSTSYSIKTYYKSQSPITNVSVSSFQQFSGFVYSGPQILESRTSVPSHLAVNFSVLSFTQNCAWLILIVPLDFTIGPQNPPLISMTDCAQNRYSIDLSTTSQSGNSVFYSSSSQQIFISCQTLIQSAVQSIGGGTQTPLQSLQCNNNTLTITQVESPKVLRQSQQLQTVIVEFDPTLPSTPNPPSPPLWYSPNLDATSFQPGYVFPLNEHQYQLQIPITQYFQPQQVSLVQSTSYQNDVSNFTLQFSLPVFMRANNHTLTVILPLTLILNREGDVSCTGNSTIFLLNPTNRQDSFPQISLTLKVTQNTSPSYSVFCQFYSAARYQTVNSISSLSQSSDPLQVIVYYLNEITSNTTTSVNMTNGLLQGPWIDFIPNQPLLISSNLVGKRGVDYTFSIVRVTVPNSIDPQNRIAIIQLDASLTLDPSFQCQILQSSNNQPFPCYQLQSNQIYFNSSIISSSGPQTLTITAIKNPQINLASVNQISSNLTFSFLYAWAQDIGLPNQTIYTIQNNTFTTPIQFSCSDSCSGCYSLYTNCTQCSSASPFIRINNSTNSISCLQSCGVLEVPVNSICTTCQATTQYCSSCSSSNLTSCSSCQSGYSYSSDWSECLDSAFFSKGSTRTLLKDISAKGPPTSLQNSEDQNSNKRITQQNDTMSGQSNLDSKTQGNSNSSQTSSLADSLKAITNGGRVVFAWAIGGAALICVGVKIAHVFQEKTKRKRDSQTDRRECLPPSEMNVIMFGQTDYRATALFLCLLSVVEVVEMPYILFWAYSASEGDLEHPVLQSLICVCSLSVILWIIDTSLLFSLLVNKQKTPITCSLFNPLPTSSRQNAGMMLQGLTTICRIIACLIPKSISLMLSNLCLCEGWTSFPCKEDEGRNSVLLTNFRKVLSSQIKSNATSIIAFITFFLLAYPAVLSSPALVYDIILFNVVMLGLCGINLRTADQILQRR

>T.pyriformis predicted MTB

MRLGQTSQYLMKEWLQNEQYNSTWIYGIQFFLNEIQTNPDYGNNIITTQSICNEISFLKTGFSASLAYFEDNFFQYLISPQQSECFQYNSQLNPNSNGYLLYFYHASNLVMFDESSTERVEISLPMMDNAKYYARFIGTQPNSIDLNFFYTLENLWVIKFYLDLNGPLLSTLNLSFPQSAVNSSFSIFTLFLKLKLEWENLNIENISNSTQLTIENNIYTITINIIQPTESITMKNINLLTDFILQFSNSGFIQQDDNTIGPFTIEIFDIDNMMLFQNSGFSLSLPYCELQIPSIVIQTPTLIIGSYQSLSLLINSNVLMRFMSSIDIQFSNDIKVRSSFGEQVYNFSSRDNYLNEIQEFGLLNQIANDFTFQQGISNLNFTTFNISFQIYINSNQINQNSNLLIQIQDNLNTSHCIRSVIQPMVLTPLLFQDLLIEYKLNQKLEIQFESQIQILATQQIRIKLPSCFQFNNSTDIQIYSGIQLNSIMQYVASNEILLLNIVDNSIGYIEQYSKIAFSLNQILYNGCQLDFSQKMIFQFENNNGVQLQKGIQQVYIDPFPLQITKIFSSYVPILNQVYNLNNQSAKIELSFIIQYNYLPLTPVVLVLLPPEVVRDNRIQNVQVSFYGDFILMCGSSNEFNITTINYFSQRTKQIQNRDAIVISCPTGQQYLQSSTNSSYSLNITGYQLPKLIQKPSDSVVIYLAQSAPSYTTTQQIFQQSDHSSIPYQIQQQWVLINEKPLDFLNSTYNFSQESITQQEIESSFSIQLQDSLSEGDLLTLSVYKEDIQTLGIASKQTGSQLTPINQILNCTAILPVVISSSVLNECIFTEDNSHYNIIASMHIPSNTSWDQQELQLQANLLLQPIIYDSVPSIVFSQSSPDLYLISQSKDSVSFASSLTSGLSISEQISTGTDIIEEHNTFYFEQILINFSSFFDLAQRDSLQMRLQLFSTVLFNESSYCTLSLPNQAIVEVPCGFQQESNQILIPELQAFFSNFDTATISEFSLAIFNTSMDQIASLQPPYTLTVNWTLEAMLNSTVILSGSPSSALPLSCTQSQCSLCVNSGEICNECINGYYLYEDISFCVQQCPPNTSVDEVTKTCKSCNTQQSGCQSCLSTDINACTQCTAGFSINAGQPASYCYIPQPVILVYVPEKTVINSSSSNSGNSKTSSESSNAIMEFIQNSTKGLLLSSILPVAGLCSVLSFLVHKLLKIKGKAQRPNLQLDSEVPQSDRSSMDSQVSIRSDQNSIPFSWVAVLLFFTNFGDLLEVPYILLANLNSSSVTDVSLSKESALQISLWIYIGLHFLSYLSCLFISFQAFVFTSYTSFPVFSIFTTSDTANSMNQHIPFARSHTPQAADSPPHRRDRDSHLESSRSFNFPKNAPFKTSYSKYMKQAFNFATRFLIALLGQAFSMLFTNIANAQGWFTISFKQNYQLIRQLYRILVIHTMLNILKCIFFSFILTDSPLSSLFSTSSSKSSSIFSHSENGILFSPFFDLLLFKLVMSVISFSNCSHVRSLLNNQDQLMQLNYSPNLTSQTSPLSDSNTGGITAFIKQSGIKAQSSSKIRHTSNDRQSSPLKIEDFQLDNRNEQQGFKERSLSPYSKSSTPNLLATSSHQTSPYRLKL
