# Supplementary material for: Selecting One of Several Mating Types through Gene Segment Joining and Deletion in Tetrahymena thermophila
Source: PLoS Biol. 2013 Mar 26;11(3):e1001518. doi: 10.1371/journal.pbio.1001518 (PMC3608545; doi:10.1371/journal.pbio.1001518)
Supplement: Text S5 — Sequence alignments of predicted mating type proteins from different species. The MTA and MTB proteins from sequenced T. malaccensis (Tmal) or T. elliotti (Tell) strains were aligned to the MTA and MTB proteins of T. thermophila (Tthe) using Clustal Omega. In each case, only the mating type proteins with the highest degree of similarity are aligned. TM exon regions are highlighted in yellow. Symbols in the bottom row are as in Text S2. (DOC) [file pbio.1001518.s016.doc]

**Text S5. Sequence alignments of predicted mating type proteins from different species.**

**A. Tmal *MTA* vs. Tthe *MTA*4**

Tthe_MTA4 MIQAFNQGLTYCEVITNDEASILNQTKSFQNFNDKVYKVNQLAFWSKNLNNTFFPFAQNS

Tmal_MTA MIQAPDQGLTYCQIQTNDEASILNQPKNQNYFNDKVNTVNQLAFWSKNLSKTFLPFPQNA

**** :******:: ********** *. : ***** .***********.:**:** **:

Tthe_MTA4 FERNISLNFYDASMNFLGTSYIYSKVMIKKVLQNYIQDANNNKMLQISLQTYGQFQIIDK

Tmal_MTA FERNITLNFYDANMSFLGPSYIYSKVMIKKVLQNYIQDASNNKMIQITLQTYGQFQIIDK

*****:******.*.*** ********************.****:**:************

Tthe_MTA4 TNFYDFFQIKFYTNSALLSSPQDLFSLQADGQT-----INSYSSYTELFITQVIYVKNFN

Tmal_MTA TNFYDFFQVKFYTNSILQSSPQNIFSLQADGQVYIYVMINSYSSYTELFITQLIYVKKFN

********:****** * ****::********. **************:****:**

Tthe_MTA4 QSDFPDKFQNLQLIFSEYRFQNINLQILFGSTNIKVTDYNQYYSALQLQNNNPQAIIRPS

Tmal_MTA QSDFPDKFQNLQFTFSEQTFQSINIQILFGSTNIKVTDYNQYYSFVQLQNSNFSAVIRPS

************: *** **.**:******************* :****.* .*:****

Tthe_MTA4 SFLSQIKNELAFNITDVVFLQCLLSSILLPSEFQNEFITAEPSLFVFYPTNKTFQNQETY

Tmal_MTA LAFSQIQNEIAFNITDIVLSQCLLQSILLPSGLQNQFMKSEPNFFVFNPTNNTYQNYGTN

:***:**:******:*: ****.****** :**:*:.:**.:*** ***:*:** *

Tthe_MTA4 YMQGNISLKVSQNFFPNRANNQIQILISPFSDIVGQNKQLNVAFNCQNNIQTIKNISISS

Tmal_MTA YLNGNASLQKSQGFQSTKGSNQIQMLISSFSDVVGQNSQLTLGFNCQNGIKTLKNLSISS

*::** **: ** * .:..****:*** ***:****.**.:.***** *:*:**:****

Tthe_MTA4 NELLNANQTQTQIKFDNNQVKMLSINIQIPQVQVSNTSLFIQLPYGIDYLQMNQSSLSVQ

Tmal_MTA NELQNANQTQVFILFNNSQAKSLSIQIQIPDIKVSNTNLFIQLPQGIDYQQKNQSSLSVQ

*** ******. * *:*.*.* ***:****:::****.****** **** * ********

Tthe_MTA4 GFAYSQFNWDQNLISFYSTNFTSSSILISIKQVQLSSSSNNIQNVTKIIAKCILNQAFVF

Tmal_MTA GFAFSSFQWDQNIVSFYSTNFSSSSIAVSIQQVQLQSSSNNIQNVTKIIAKCILNQAFVF

***:*.*:****::*******:**** :**:****.************************

Tthe_MTA4 YVDSNSNQTIQILQPQPLPTTSIQINTFNQTQSTSLSSPQNRTSLQSQLAFSFSILNFQD

Tmal_MTA YVDSNSNQTIQIIQPQPLPTTSIQINTFNQTQFTSFTSPENRASLQSQLAFSFSILNFQD

************:******************* **::**:**:*****************

Tthe_MTA4 TCSWLIVSLPYSFTIGFLDKSQFTLFDCFGNTYSYTQGNPISSQSTIGYTDNNNCIYISC

Tmal_MTA TCSWLIVSLPSSFTIGFLQNSQFNLFDCFGNTYSYTQGNPISSQSTIGYTDNNHCIYISC

********** *******::***.*****************************.******

Tthe_MTA4 KALRSVSSSHAANNCLNNTVTIRNVRSPDFPLQTAGIQFLIANSNTSSQPNSDPPTFFNQ

Tmal_MTA KVLRSVSSSHAATSCLNNTVTIQNVRSPDFPLQTAGLQFLIANSNTSSQPSSDPPTFFNQ

*.**********..********:*************:*************.*********

Tthe_MTA4 STDLTNSSLPYFFIGSEVFTSKGINISQVDLSTLSTQISSNYFGDVFNFSMILAYPIYFW

Tmal_MTA SIDLTDSSLPYFFIGSEVFTHEGLNITQADLSSLSTQISSNYFGDVFNFSMTFAYPIYFW

* ***:************** :*:**:*.***:****************** :*******

Tthe_MTA4 EQHQIDVQLPFRLFAGKSDIECQPISTVVCSLQASRD----PQSTIFRIQVLNLTAPNTQ

Tmal_MTA EQHQIDVQLPFRLV-GKQGIECWPMYAVVCSVKGIGELQSQSQWTTFRIQFVNLTAPNTV

*************. **. *** *: :****::. : * * ****.:*******

Tthe_MTA4 IGLQVSYVVLSQNFQLDQISFLIISVSLSNRIINYQNKTIDAFKNLAIQNLITLQNQQPL

Tmal_MTA ITLQISYGALNKNIQLGQQLFLGISVNLQNRIINYQNKSIDAFNNLTVQSLVTLQKEQPL

* **:** .*.:*:** * ** ***.*.*********:****:**::*.*:***::***

Tthe_MTA4 QISNPLLGITGVNYTFNIQQLQIPQNINNYYYLSLSLDPSFQYNSSQIAFYQLIQSQN--

Tmal_MTA QISNPLIGMLGVNYTFNVQQFQIPQKVDQNYYLSLNIDSSFQLNSSQISFYQLTQSQNSS

******:*: *******:**:****:::: *****.:* *** *****:**** ****

Tthe_MTA4 -LNTFIENRIDFISNSSNLFLIPITSLVPSYPFQLRISGLRNPSGIVEQNEQNVAQTYNF

Tmal_MTA GTNNLIEHSIEFIYNSTNSFLIPIISLVPTYPFQLRISGLRNPSGIVEQNEQNVAQRYNF

*.:**. *:** **:* ***** ****:************************** ***

Tthe_MTA4 QLIWSTQQGNSFKNVWVIQSVSLPITSKYTCSPNCQACASNYAACTACAPGYLKSQY---

Tmal_MTA QLIWSTQQGNSLKNVWVIQSVSLPISSKYTCSPNCQACASNYAACTACAPGYLKSQLQQQ

***********:*************:******************************----

Tthe_MTA4 ----NHHAVLACLPTCSPQYVAYNGTCLACQLKDPQCLSCSPSNLTQCSSCNQGYTLVPE

Tmal_MTA QQYNDHAAVLACLPTCSPQYVAYNGTCLACQLKDPQCLSCSPSNLTQCSSCNQGYTLVPE

----:* *****************************************************

Tthe_MTA4 FNGCVDSHLLQTARSRLLDYSLSTNLADNIDTTHP-------------------------

Tmal_MTA FNGCVDSHLLQTPRSRLLDYSLSTNLPDNIDTHPDDDNDDEDDEEQEEDDDEADEEEEDE

************ ************* *****----------------------------

Tthe_MTA4 -------------DDAQKSDKAQSLTRMTEESSSAKADQREGGESSSNTGSKVMGQLQDT

Tmal_MTA ADADDDDDEEDEEEDAQKSDKAQSLTRMTQESSSAKADQREGGESSSNTGSKVMGQLRDT

-------------:***************:***************************:**

Tthe_MTA4 VGALKGGGAIFIWIVLAALAVSVSQSVLRYTYNKLKGKSHSNGSSSGMRRSS--------

Tmal_MTA VGALKGGGAIFIWIVLAALAVSVSQSVLRYTYNKLKCKSPSNGRSSSGSSSNSSSTKNNN

************************************ ** *** **. *.--------

Tthe_MTA4 -------------SGSRSSSSGSSSAGRNASGKEHKGVREREKELRGQRIDCLMLFLLSV

Tmal_MTA NKNNNIRRSSMRRSSSSRSSSGSSSGARNGSWKEHKGVSERAEELHGQRIDSLMLFLLSI

-------------*.* *******..**.* ****** ** :**:*****.*******:

Tthe_MTA4 AEAIQMPYTLLWGFSVSGGDFESPVLQTLLGACALSTLLWIFDAYLLGSILANSENTPKT

Tmal_MTA AEAIQMPYTILWGFSVTGGDFESPVLQTLLGACALSTLLWIFDAYLLGSILANSENTPKT

*********:******:*******************************************

Tthe_MTA4 SCLLNPLPSSKREGCLSFLIVPARLAFCLIPKSVSLTLTNIFSVEGWFRYPYGEDVERAT

Tmal_MTA SCLLNPLPFSKRKGSLSFVIVPARLAFSLIPKSVSLTLTNIFAVEGWFRYPYGEDVGRAT

******** ***:*.***:********.**************:************* ***

Tthe_MTA4 IVLTNFRKVLSSQIKSNVSGLCSLVSFLILQYPEVLSSYVQIYDLIIFDIVMTILCLTNI

Tmal_MTA IVLTNFRKVLSSQIKSNVSGLCSLVSFLILQYPEVLTSYVQIYDLIIFDVVMAILCLTSI

************************************:************:**:*****.*

Tthe_MTA4 RNVDKLISTLNQIQESEGEL-----

Tmal_MTA RNVDKLISILNQIQENEGELWTNAK

******** ******.****-----

**B. Tmal *MTB* vs. Tthe *MTB*4**

Tthe_MTB4 MNASQIIYLLIFVAALLIERSQATLSCYLINPTQSLKQIYQLPFNYRQGSDAAPAQYNFA

Tmal_MTB MNTTYKIAFLLIFASLLIDRSQEALICYPINPIQSMKQIYQLPYNYRLGSDPVPSQYNFA

**:: * :*::.*:***:*** :* ** *** **:*******:*** *** .*:*****

Tthe_MTB4 LQYDQLSPDVQYTLQNIAYLKRYLITSNNQVISIIPNTIGDSLNYIPPELIQYTFNIVTI

Tmal_MTB LQFDQLSPDLLYTLSSIAYLKRYLITSNNQVISIIPNSIGDSLNYIPLELTQYTFSIVAI

**:******: ***..*********************:********* ** ****.**:*

Tthe_MTB4 YQSIANFSDQPDFLTSYCQITQFPQMDLTTFRQVNKQNQQFLISAILLSQIEDLTSLVLS

Tmal_MTB FSSLSSFSDNQDFLTSYCSITQFPQTDLTIFRQINKQNQQFLISTMLISQIQDLNSLVLS

:.*::.***: *******.****** *** ***:**********::*:***:**.*****

Tthe_MTB4 YPTSFALKSQNAANLGCQLYTDLGIINSCSFSLNAQQGLSLVTFNLTNFNISLQNISATI

Tmal_MTB YPTSFAIKSQNAANLGCQLYTDYGIINDCSVSLNAQSGLSFVTFNLTNFNFSLLNVSATI

******:*************** ****.**.*****.***:*********:** *:****

Tthe_MTB4 VINYTTFNQNLLSNKQFSLQLLNQYQSQISQSSQFQIVDQSICQQTTFSANLINNLQTFG

Tmal_MTB VLNYSTFNQNSLSNKQFSLQLLNQYQSQIAQYSSFQIIDQRICQQTTFSASLINNVPSFG

*:**:***** ******************:* *.***:** *********.****: :**

Tthe_MTB4 QNLKLKINFIPASSNITRIIIQFSSQIYAMIELSHPQLVFIDSSQTTKFKVRCLKAYQFL

Tmal_MTB QNLKLKINFVPASANITRIIFEFSSQIYTKIDLSFPYLTILDSTQTNQQKVRCLKAYQFM

*********:***:******::******: *:**.* *.::**:**.: **********:

Tthe_MTB4 LVCDLIGG-LPFDVSQGVYAELPYIQVKVPSYPQSLTFQIKYFTDLSYQNCFVSNLTQKF

Tmal_MTB LVCDLIGGNLLFDVTKGVNIEFPYVQLNVTSYSQTLSFQIKYFTDLTFQTCFVSNFTQKF

******** * ***::** *:**:*::* ** *:*:*********::*.*****:****

Tthe_MTB4 QAAIPTSPQAYLYNNSSLQIKLFDLIMLDNSTVINLSLPSQFSLSSSTQINSIVGISNQS

Tmal_MTB QAVSPSSPQAFLYSNNSLQIRLFDLIMLDNSSLINISLPSQLAFTSTSKLSQIAGISNLS

**. *:****:**.*.****:**********::**:*****::::*::::..*.**** *

Tthe_MTB4 QITLSSQQNLQISQISLSLAQLMTYQGISFQLTKIIQNTVNCNLSSLSQIQIEIVNSFGV

Tmal_MTB QTQQKTQQNIQISQIGLSLAQLKTYQGISFQLT---NNTIYCSLSSLNQIQIEIVNSLGV

* .:***:*****.****** ********** :**: *.****.*********:**

Tthe_MTB4 IVLSGSIPLQIQPYPIYIQQMNAVFTPLSQQQLLQYQQQQNSYTVPFVTLEVNFQIQINY

Tmal_MTB IVLSGSIPLQNEPYPIQIQQINPIFTPLNQQQQLQYQQQQNSYSIPFVTLEINFQIQINY

********** :**** ***:* :****.*** **********::******:********

Tthe_MTB4 FPQSSAFVIYLPPQIIRDNRVNNISIEIANQFNFYCSSQTNSTQFFSSNITFQNKIQQRD

Tmal_MTB FPQSSAFVIYLPPQIIRDNRVNNISVEISNQFNFYCSSQTNSTQFFSSNVTYQNSIQQRD

*************************:**:********************:*:**.*****

Tthe_MTB4 SISILCSFSGNITTNNPQYLFTAKIQGYLLPKEFQKPTDRIIITLIDYSQKQYQTQYQCQ

Tmal_MTB SISILCQFSENITTNNPQYLFTARIQGYLLPKQVQKPTDRIIINLLDYSQKQYQPQYQCL

******.** *************:********:.*********.*:******** ****

Tthe_MTB4 STENLSISEQIKSQWVFNNNQSTDLSQNQYYYYQNSTTLNSNQSQNVPYSLINLTTRYPI

Tmal_MTB STESSSTSEQIKSQWVFNNNQGTDLLQTYYS-----TLSISNQNQSIPYSFVNLTTRYPI

***. * **************.*** *. * * ***.*.:***::********

Tthe_MTB4 QDGDYIVVSFSKLQFLKNEVINGIQ--ALAYPELKNLNCSFLQPQS---SFLNLFSTCNL

Tmal_MTB QDGDTIIIRFSKLQFFKNEVINGNQQQLIAFPELKNLNCSFLQPQSNLSTFPNLFSICNL

**** *:: ******:******* * :*:*************** :* **** ***

Tthe_MTB4 SEGSSQYSINLQLYPTQLDSNLSWNYKDLALSVAGLQFFPNQQYYNSSVQFQHISSNKYI

Tmal_MTB SESSTWYSINLQLYPTQLASNLSWNYKDLALSLVGLQFYPNQLDQNQTVQFQHISSDNFL

**.*: ************ *************:.****:*** *.:********::::

Tthe_MTB4 ISQSDYNFISENNVVAQAFYSFNKINSEQFQSLDSQSQRLTFDYIQLQFQTTLNLSSSSQ

Tmal_MTB ISQSNYNFTIKNNIASNALFALNKIKSGQFQSLDQQASKLLFDYIQLQFQSSIFLLPSIQ

****:*** :**:.::*::::***:* ******.*:.:* *********::: * * *

Tthe_MTB4 NLSLTLQFSQPIYLSNLSFCSIDVGCSQSTLISTCSLSADGLLVQINKLDQFSTCASSVL

Tmal_MTB NITLTLQFSQEIQLSDDSFCSIDIGCSQRTLILKCNLSLDGLYIQIDQLGQYSTCTSSVL

*::******* * **: ******:**** *** .*.** *** :**::* *:***:****

Tthe_MTB4 NNFNITIHNPEINNQNSTGGAAQSIAISWNLTSTTSSQTLLSGHTSLTTNTSQCPQPHCA

Tmal_MTB NNFNITIHNPEINNSNSTGG-AQSIAINWNLTSTTTSQTLLSGHTSLTTNTSQCPQPHCA

**************.***** ******.*******:************************

Tthe_MTB4 TCTSPPSICIHCTQGYYLLPDQNSCVQTCPPPTVAHQQTATCQPCFQHQECLQCQSQNPA

Tmal_MTB TCSSLPSICLHCTQGYYLLPDQTSCVQTCPPPTVPHQQTPTCQPCFQQQECLQCQSQNPA

**:* ****:************.*********** **** *******:************

Tthe_MTB4 ACTSCSPTYSLNSTLLPYCYVPLPPSSSA-----------------------SSSVTKDV

Tmal_MTB ACTSCSPNYSLNSTLLPYCYVPLPPSSSGPSSSSTSSSPSSSPSSPSSSSSSSSSVTKDV

*******.********************. ********

Tthe_MTB4 VNRTPSNSTFSGALNRPEPGQPSQKQQQQQQQQQQQEQQQQQEQQQQEAQASDQRGFAHF

Tmal_MTB VNRTSSNSTFSGELNRLERGGQQ----------------QQQSQKQQEAQASDEGGFASF

**** ******* *** * * . ***.*:********: *** *

Tthe_MTB4 LAQTKSYTKGFILTLLIPLSILGACLTRLVTFCLKKREKKVHPPLPSESRSAQIAQNLDE

Tmal_MTB LAQTKSYTKGLILTLLIPLSILGACLTRLVTFCLKKREKKVHA-QPSESRSAQIAQNLDE

**********:******************************* ***************

Tthe_MTB4 RQETQKDGNGGDEEQMRASSRVDTANMCPLNSRRGEQLQLEGVQQQSDGGVGGGESEGNG

Tmal_MTB RQETQKDGNGGDEEQMRASSRVDTGNMCPLNSRRGEQLQLGSVEQQSD-GAGGGEREGNG

************************.*************** .*:**** *.**** ****

Tthe_MTB4 YLAFCWIAVILLLGNVGDLVEVPYIIFSQQNSFSNKSTTNVFDLQFSEADMGQICCLSYI

Tmal_MTB HLAFCWVAVILLLGNLGDLVEVPYIMFSQQSSFSKKS-TSVFELQFWEADMGQICCLSYI

:*****:********:*********:****.***:** *.**:*** *************

Tthe_MTB4 ALNAVCYLICVVMMVKAIIFETGSGEPLFCIYEVKLSSSSSFC---GEEMKKDKSEAAAC

Tmal_MTB ALNGICYLICVVLMVKAIIFESGSGEPLFSIYEVRLSSRSSFCSVGGEEMKKDKSE-VEW

***.:*******:********:*******.****:*** **** ********** .--

Tthe_MTB4 KGDSNAQNENGEKHKKVLSGRKLWKLIIDVFLRCLVVVGGKAFCMVYSNVANVKGWLTCQ

Tmal_MTB KGDSNRQNENKEKHKKGVSGRKLWKLTIDVLLRCLVVVGGKAFCMVYSNVANVKGWLTCQ

***** **** ***** :******** ***:*****************************

Tthe_MTB4 VDKNLRAFRLFYMTLCIHAIFNMISAVFFTLMLTHFSFASWTAV-AQDSLTSQGSDGGVE

Tmal_MTB VNKNLRAFRLFYMILCIHAIFNMISAAFFTFVLTHFSFVSWAAVVGQDSLTSQGIDGRVE

*:*********** ************.***::******.**:** .******** ** **

Tthe_MTB4 FSFFVDILAFKFLMSLICFLNCLHIQQLISACKSPNLPGPQNPVSHRLQSPSTPSSSASP

Tmal_MTB FSFFVDILAFKFLMSLICFLNCMHIQQLIAACKSSNLPGHQHSTSHPVQSPSTPSSSPSP

**********************:******:**** **** *. .** :********* **

Tthe_MTB4 ADAVERDACKVSYFENTPNAAEKTTPTAVTLASYRQQETSGTGSQNLIEGRPRRKKPSKL

Tmal_MTB AGAVARGVGKASYFENTPNA-EKTTPTTVTLASYRQQDASGTGSQNLIEGRPRRKKPSKL

* ** * . *.********* ******:*********::*********************

Tthe_MTB4 SLLLKRDSGQKSGSSLSSRQQETPSPNLPSYSPNLYPSQAYI

Tmal_MTB SLLLKGDSGQKPGGSSSSRQQETPSPNLPSYSPNLYPSQAYI

***** ***** *.* **************************

**C. Tell MTA vs. Tthe MTA3**

Tthe_MTA3 MMI-LIILICSLFGALRSAKSLDQGIIIEVKHGDYKFIKIVTNDESSIKNFNGKLDQLNS

Tell_MTA MMIVFLFVISTLLKVNIQARSIDQGIITEVKPQEYKYLRITTNDESAITRFDGKIELFNS

*** ::::*.:*: . .*:*:***** *** :**:::*.*****:*..*:**:: :**

Tthe_MTA3 KIFTINQIVFQQPKEDYCFLPSPQNTFGRIISQEFYDENMNLINSAISNIHGQVLIKNTT

Tell_MTA KIYKTNKLLFDKPQENFYFLPLTQNTFGRLVSQDFLDESMNVLNAGISNIQSQVIIKKQN

**:. *:::*::*:*:: *** ******::**:* **.**::*:.****:.**:**: .

Tthe_MTA3 QKLIDAGNGQKWLSVILNAYQQFQIISNQINSIFQIYTTIPNLTTNQSAQSIFSLLIDGK

Tell_MTA QKLIDGNNGKKWLSIIFNTYQQFKVMSNQINSRFDINVLIPYIKSSQPSIDIFSLLIDGK

*****. **:****:*:*:****:::****** *:* . ** :.:.* : .*********

Tthe_MTA3 KMNCFFNQTNTGIKNGTLFTQNFYLSDFPTQFQDLEIQFFNPFFPTFSLSISSGADFLQF

Tell_MTA KINSFLNQTNNAVKDATLFSQSFNLADFPLQFNDLEIQFLDPFFPSLSITLSSGADYQQI

*:*.*:****..:*:.***:*.* *:*** **:******::****::*:::*****: *:

Tthe_MTA3 QISNQLYSSYLLYNSNPSAMIIPQSDNLIANQLVFILQSDIFSQCYVQQLDLPSNMNTQF

Tell_MTA QIVNQFYSAYQLYNSDYSAIILPQIENPIISQLVFSLKSNIFNQCLVKQIGLPQEFISQF

** **:**:* ****: **:*:** :* * .**** *:*:**.** *:*: **.:: :**

Tthe_MTA3 SFSQPVLN-VQNPQNRSQS-SIIDSNFYNGTISFVRNQNLRVLPSQVSYSDT--LVAISS

Tell_MTA PLTNSLVLQTQNSESSSQIFAIDFFNFINGTIFFPQNQTFTGLPSQKPTSNAITNIIISS

::: :: .** :. ** :* ** **** * :**.: **** *:: : ***

Tthe_MTA3 FLDVIGFDNNLTMTFVCSQNLIQKKSIRIIKNEYLNANQTQVSIQVNNLVGTKNISIQIS

Tell_MTA FLDLNGLDKNLTMALFCSETETANLSVEIVKNEYQFANQTQAAILINN-SGTKSIKIEIS

***: *:*:****::.**:. : *:.*:**** *****.:* :** ***.*.*:**

Tthe_MTA3 IPSVTIQNTSLIMQLPQSILFQDNTQQSLDVQGFVYSNFVWQQNNLAFFGANFSSTILRL

Tell_MTA IPSINIQKASLFLQLPRTIICQNTTQESFTVEGFTYSSFVWQNYQIVFNSVSFTSTTLRL

***:.**::**::***::*: *:.**:*: *:**.**.****: ::.* ...*:** ***

Tthe_MTA3 SFTNIDVKNQTSNVDILNSTFIIAKCIFNQTFVFYVDSNTNQTVKIIQPTPLPRSQITLS

Tell_MTA TFDNIEIKSKTTEADIFNNSKIIAKCIFNQTFVFYVDSRTTQTVQIIQPPPLQLTQMQIN

:* **::*.:*::.**:*.: *****************.*.***:**** ** :*: :.

Tthe_MTA3 SFNQTKVNSLDVPESRTSVQSQIAFSFSVLNFLDTCSWLIVQIPFEFTIGFIQMSQFTLQ

Tell_MTA SFNQTKADSLNTLENRTSVQSQLAFSFSILDFQSTCQWVIIQLPLEFTIGFMKMSQFNLQ

******.:**:. *.*******:*****:*:* .**.*:*:*:*:******::****.**

Tthe_MTA3 DCFGNIYSYLQGSSILPQSMISYTDNNNCMYISCSSLRLNSQMNSSSQCMNNTVIIQSIK

Tell_MTA DCSGSQYSYLQGSTQSSLSTIIYTDNNSCIYISCQSLRKASQHNSKTQCLDNIVTISSVK

** *. *******: * * *****.*:****.*** ** **.:**::* * *.*:*

Tthe_MTA3 SPDQAVQTSSLNIYIANQNSSAPSNIGPPTFFTQNLNLKNNSLPYFFIPTEVYSHVGINI

Tell_MTA SPDLAYLTLPLKMFIANQNSTINDDAGLPTFFNEDLNLQNQSLPYYFIPNEIFTYPGINI

*** * * *:::******: .: * ****.::***:*:****:***.*:::: ****

Tthe_MTA3 TEADFSDLNFNISSNYYGDVFNLTTTISYPFYLYQQHQISIQIPVRILANQSVVCYPESF

Tell_MTA TEVDLSTVYFNITSNFYRDVFNFSMVFAYPIYLHDQHQINIKIPIKIMGNQNIQCQPSSF

**.*:* : ***:**:* ****:: .::**:**::****.*:**::*:.**.: * *.**

Tthe_MTA3 LSCSIKIPINLNFTTIL-IQILQQVSPNTKIAFQLNQVVASQSLLQGKQAFALIQVSSTS

Tell_MTA LYCSLKIDTSENSQTIVQIKFLQQIMPDTKIYFSLDQVVANNDPLQNQQSFAFVQLVLSN

* **:** . * **: *::***: *:*** *.*:****.:. ** :*:**::*: :.

Tthe_MTA3 KIINTKNKTIDVLNNLKLQSWINIDAIQPLQISNTLLGQEGVNYTFAILSLQIPSNQLDN

Tell_MTA RIVNTKNITIDILNGIKYYKFISLDETSPLQISNSYLNFANVYYTFNIKSLEIPPEEQNN

:*:**** ***:** :* .:*.:* .******: * * *** * **:** :: :*

Tthe_MTA3 YYLSLKIDQSLSYNSSNSNCYSLTQVTGDNS----QREEKVNCINFSSNTFLIPISKNQI

Tell_MTA YYLTLKMDSSIQFNSSSSSCYILTQTTNKESPDSSFNELNLNCQVDSQNNFLIPVSQILF

***:**:*.*:.:***.*.** ***.* .:* .* ::** *.*.****:*: :

Tthe_MTA3 SQPFQLRITGLRNPSGIVEQ---NVAQTYNFQLIWSTQQGNSFKNVWVIQSVSLPITSKY

Tell_MTA SEKFQLRISGLRNPSGILEQNEQNLAQTYNFSLIWSREQANRVRNVWLIQSVSLPITYKY

*: *****:********:** *:******.**** :*.* .:***:********* **

Tthe_MTA3 TCSPNCQACASNYAACTACAPGYLKSQYNHHAVLACLPTCSPQYVAYNGTCLACQLKDPQ

Tell_MTA TCSHNCQGCASNYAACTTCAAGYLKSHHNDHTVLACVATCRPFHVAYNGSCVACQLKDPY

*** ***.*********:** *****::*.*:****: ** * :*****:*:*******-

Tthe_MTA3 CLSCSPSNLTQCSSCNQGYTLVPEFNGCVDSHLLQTARSRLLDYSLSTNLADNIDTTHPD

Tell_MTA CLSCSPSNLTECSSCNQGYTLMPEFNQCLDSRLLKTGRSRLLDDSLSTNLAHNIDTHPDD

**********:**********:**** *:**:**:*.****** *******.**** *

Tthe_MTA3 --------------------DAQKSDKAQSLTRMTEESSSAKADQREGGESSSNTGSKVM

Tell_MTA DNDDDEDEEDYAATTAADDNDAHKSHKAQSFTRMTQESSSAKADQTEGGES-SNSGSKVM

--------------------**:**.****:****:********* ***** **:*****

Tthe_MTA3 GQLQDTVGALKGGGAIFIWIVLAALAVSVSQSVLRYTYNKLKGKSHSNGSSSGMRRSSSG

Tell_MTA GQLQDTVGALKGGGAIFIWLVVAAFAVSVSQSVLRYIYNKLKCKTHSKGSGSSDHSTMRR

*******************:*:**:*********** ***** *:**:**.*. : :---

Tthe_MTA3 SRSSS--SGSSSAGRNASGKEHKGVREREKELRGQRIDCLMLFLLSVAEAIQMPYTLLWG

Tell_MTA NSNSQSGEVSGFEARNKSGKDHKRVSEKEKELSGQRIDCLMLFLLSLAEVCQMPYTLIWA

. .*. . *. .** ***:** * *:**** *************:**. ******:*.

Tthe_MTA3 FSVSGGDFESPVLQTLLGACALSTLLWIFDAYLLGSILANSENTPKTSCLLNPLPSSKRE

Tell_MTA LSVTGGDFESPVLQTLLGACALSTLLWIFDAYQLGSILANTESTPKTSSLLNPLPSSRRT

:**:**************************** *******:*.*****.********:*-

Tthe_MTA3 GCLSFLIVPARLAFCLIPKSVSLTLTNIFSVEGWFRYPYGEDVERATIVLTNFRKVLSSQ

Tell_MTA GSLSFVIVPTRLIFCLIPKSLSLTLTNIFPLEGWFRYPFAEDFEGAAILLTNFRKVLSSQ

*.***:***:** *******:******** :*******:.**.* *:*:***********

Tthe_MTA3 IKSNVSGLCSLVSFLILQYPEVLSSYVQIYDLIIFDIVMTILCLTNIRNVDKLISTLNQI

Tell_MTA IKSNITSLCSFISFLILQYPEVLTSYVQLYDLIIFNFVMTVLCLTNTRNLDKIISILNEI

****::.***::***********:****:******::***:***** **:**:** **:*

Tthe_MTA3 QESEGEL

Tell_MTA QDNE---

*:.*---

**D. Tell MTB vs. Tthe MTB3**

Tthe_MTB3 -----MTDCKIFIWFYILYLISKFCKCQVDCYLAS-STQIGQQIQPIAYSQRQGQTSFGA

Tell_MTB MKFSPLNNQKVAFYFMILHLVRKLAEGKGSCFLTSSSTQTWQQIQPVAYSQREGFTNFGP

:.: *: ::* **:*: *:.: : .*:*:* *** *****:*****:* *.**

Tthe_MTB3 QYFFTIQLASNQTDILQGTSSVQFTQYFLTSNKFVLSSMISKNMGLLPTPQQYVNSTYYV

Tell_MTB QYLFTVQLSTNNTDALLGTESTQLTQFFLTSNQFMLSKIVSKVAGFQQ-PPQLQKTTFYI

**:**:**::*:** * **.*.*:**:*****:*:**.::** *: * * ::*:*:

Tthe_MTB3 AAAFTYQQFISGALDFSSICNLDQYPLLTLKEFVQVNTQTQQMFVSVPLTSQINDVYYLT

Tell_MTB AAAFTSQQVSSGLVDFGSVCDMSTYPQLALREFVLVNSQTQQLFIQVPIISQINDIYYLN

***** **. ** :**.*:*::. ** *:*:*** **:****:*:.**: *****:***.

Tthe_MTB3 LFYPTSLASQPQNNNNLGCQLYTDYIVINQCSLVINSTLTTVSFNLSSINIRNISATLVL

Tell_MTB LQYPTSLALHTQDNNNIGCQLYTDYIVINECQLISSSGYTTVSFDLSKMIARNISATVVL

* ****** : *:***:************:*.*: .* *****:**.: ******:**

Tthe_MTB3 STATFNQNFIKQNQPFQIQLLNQLQNKIAQSPAFGAKDYRQCQLTNFQAQLIFNNSYSIS

Tell_MTB SSGTFNQNLINQNKPFRVTLLNQLEYLIAQSPNFNAINYNQCSQTNFSAQVLYNSSYQIN

*:.*****:*:**:**:: *****: ***** * * :*.**. ***.**:::*.**.*.

Tthe_MTB3 EQRLQVQFATASPAISRILIEFSSQILPKFNLTNVLVMVSDLSKLNPYAVFCQKAYQSII

Tell_MTB EQRLQVKFTPVSSTISRILLEFSTPIIPKFSLTSITVIMSDYSLQNSFTVLCQKVYDSII

******:*: .* :*****:***: *:***.**.: *::** * * ::*:***.*:***

Tthe_MTB3 LCE-LFSPASPFDMSKGVILTLPYFQPMQAVPQNSYSFTVKYFTDSTYQTCYNTNITIPF

Tell_MTB LCDGVFTSVPTFDISKGLILNLPYFEARQAVPPSFHTFTLKYFPDYSFQTCFYTNSTIPF

**: :*: . **:***:**.****: **** . ::**:*** * ::***: ** ****

Tthe_MTB3 IPQDIESPVAFLFNNDSLLLQFSNVINLETSTTINIQLPSQLSFAPTSQLAQITGISSLS

Tell_MTB MQQKVQTPFAYLFNNNTLQVSFSNVINLENATIIDIQLPTKLSFPSTAEINQISGISILS

: *.:::*.*:****::* :.********.:* *:****::*** *::: **:*** **

Tthe_MTB3 KILISSSQNSKISQVTQSLQQLTTNQGIQFQLLNVTRSSIFCSLTNIASLQIQIVNSFGV

Tell_MTB QISKSSASNAKITQVTQSLEQLTTFKGISFSLQNISPNSIQCNPTNPEVLNIQIINSFGV

:* **:.*:**:******:**** :**.*.* *:: .** *. ** *:***:*****

Tthe_MTB3 VIMSGTVPIQIQSYSIIVTSINSNQILNLQ-----QQDNDLTIPVSLEVNFQLKARYFPQ

Tell_MTB VILSGSILIIIEPYSIQIQSKKINQLPDTQQQQQIQQNGNFTDPVSLEINFQLNADYFPQ

**:**:: * *: *** : * : **: : * **: ::* *****:****:* ****

Tthe_MTB3 SSAFVIQLPLQLTRDNRFSQVTVEIVNEFNFFCKSQTNSTKFSTSKVTYQNTVQQRDTLV

Tell_MTB QSAFVIYVPPQLIRDTRFQTVTVQIANQFDFYCKSQTNSTKFLDLAVPFSNTIQQRDTIV

.***** :* ** **.**. ***:*.*:*:*:********** * :.**:*****:*

Tthe_MTB3 IQCQFSGNQTINISSDVFTAKIQGYQLPMQVQKPTDRVIINLVDFSQNSFNEQYYCQSSE

Tell_MTB VLCQFSGNQTISSSSDVFTAKIQGYKLDYQVEKPTDRIIVDLFDYSQNQFQKQYLCQTTE

: *********. ************:* **:*****:*::*.*:***.*::** **::*

Tthe_MTB3 NQNIPDSLKNIAVFVKTQSTNFIVSQYSYQITSYINQNKVSFTAQSLINITTNYSIQDGD

Tell_MTB SKNNPYSMKNQWVFVKTNASNFIISKYLYQVSTAIYQDNGVKPIYSLINITTSYCIQDGD

.:* * *:** *****:::***:*:* **::: * *:: *******.*.*****

Tthe_MTB3 LIEIEFGKQAFFKFEISSSSQQQISSVSEVKNLACSVIYPLPAQPNSVSFIPTCVLVEKA

Tell_MTB TIQINFEKQQFLKFETTPTSQSQIPSISVVKNLVCSTIYPLSSQPNSNNFITNCILVEKA

*:*:* ** *:*** : :**.** *:* ****.**.**** :**** .** .*:*****

Tthe_MTB3 SSFSIQFQLYPSQLGQVLNWNNKDVVLNISGLAFQENLQQNYQSEVLFSHSSSDLYLLSQ

Tell_MTB SSFSVQIQLKSSEIGQLSDWNNKDVVFKVTGLAFQENFQQSSQAEVLFQHFSNDQYLVSQ

****:*:** *::**: :*******::::*******:**. *:****.* *.* **:**

Tthe_MTB3 SNQTFNNQLSVTANSVYQVSQISTLSSLAQDYLNYKFDFIQLQFSQSILVPQSNSNFQLT

Tell_MTB STQKFANQQPMTVNLEYQINQASTFQTLTQNNQKISFDFIQLIFLQSILLPNSNQNVQLN

*.*.* ** :*.* **:.* **:.:*:*: : .****** * ****:*:**.*.**.

Tthe_MTB3 LNFSQQIYLSTLSYCSLDSLCSQRTQIVQCNLSSNSTSLTLDNIGSWITCSSVLSSFNIT

Tell_MTB LSFSQQINLSSSSFCSINSFCSQLTQTVQCNISQNSMSITINSIDTYSKCRSELNAFNIT

*.***** **: *:**::*:*** ** ****:*.** *:*::.* :: .* * *.:****

Tthe_MTB3 IHNPEINNQNSTGGAAQSIAINWNLTSTTSSQTLLSGHTSLTT-NTSQCPQPHCATCTSP

Tell_MTB IHNPEINYSNS-GGGGQPIRINWSLTSSTTSQTLLQGDTSLTTNNTFQCPQPHCTACTSL

******* .** **..* * ***.***:*:*****.*.***** ** *******::***-

Tthe_MTB3 PSICIHCTQGYYLLPDQNSCVQTCPPPTVAHQQTATCQPCFQHQECLQCQSQNPAACTSC

Tell_MTB PQICIQCTQGYYLLPHQNSCVDTCPPPTVAHPQTATCKPCLQEQECLQCQSQDQAACTSC

*.***:*********.*****:********* *****:**:*.*********: ******

Tthe_MTB3 SPTYSLNSTLLPYCYVPLPPSSSASSSVTKDVVNRTPSNSTFSGALNRPEPGQPSQKQQQ

Tell_MTB APTYSLNSTALPYCYVPLPSGSS--SPISKGVATRKSSDSRFSGGYNRLEQGQLSQKQ--

:******** ********* .** * ::* *..*. *:* ***. ** * ** ****--

Tthe_MTB3 QQQQQQQQEQQQQQQQQQEAQASDQRGFAHFLAQTKSYTKGFILTLLIPLSILGACLTRL

Tell_MTB ------------------QVQASDEGGFVDLLGEMKHFTKGFILTLLIPLSILGAWLTRL

------------------:.****: **..:*.: * :***************** ****

Tthe_MTB3 VTFCLKKREKKVHPPLPSESRSAQIAQNLDERQETQKDGNGGDEEQMRASSRVDTA--NM

Tell_MTB LSFCLKKGGKKVSAVPSASRNGQVGVENLDERQETQKDVNGGDEEQMRPSSRLDTANGNM

::***** *** :. .. .:*********** ********* ***:*** **

Tthe_MTB3 CPLNSRRGEQLQLEGVQQQSDGGVGGGESEGNGYLAFCWIAVILLLGNVGDLVEVPYIIF

Tell_MTB YPLNS-RGEQLLLQSVQSQGDGA---VQSEGTAPLAFCWIAVILLFSNLGDLVEVPYIIF

-**** ***** *:.**.*.**. :***.. ***********:.*:***********

Tthe_MTB3 SQQNSFSNKSTTNVFDLQFSEADMGQICCLSYIALNAVCYLICIVMMVKAIIFETGSGEP

Tell_MTB SQQNSSS-KQSINIFDLSFSEADMGQIFCLSYIVLNAVCYLICVVMMVKAILFENGSDSP

***** * *.: *:***.********* *****.*********:*******:**.** .*

Tthe_MTB3 LFCIYEVKLSSSSSFCGEEMKKDKSEAAACKGDSNAQNENGEKHKKVLSGRKLWKLIIDV

Tell_MTB LFSIYKVSLSNSSSYFSAGLIKKEKIEVEGKGNLSSLNQK-TNKQKVNNEPKLWQLILDI

**.**:*.**.***: . : *.:. . **: .: *:: :::** . ***:**:*:

Tthe_MTB3 FLRCLVVVGGKAFCMVYSNVANVKGWLTCQVDKNLRAFRLFYMTLCIHAIFNMISAVFFT

Tell_MTB LVRCLMVLCGKAFCMVYSNMGNVKGWLTCQVNINVRIFRQFYIILCIHALFNMAATAFFT

::***:*: **********:.**********: *:* ** **: *****:*** ::.***

Tthe_MTB3 LMLTHFSFASWTA----VAQDSLTSQGSDGGVEFSFFVDILAFKFLMSLICFLNCLHIQQ

Tell_MTB LMLTHLSFASITADAAGAAQDSLTTQSSDGNVEFSFFTDVLTFKFLMSLICFFNCLHIQQ

*****:**** ** .******:*.*** ******.*:*:**********:*******

Tthe_MTB3 LISACKSPNLPGPQNPVSHRLQSPSTPSSSASPADAVERD--ACKVSYFENTPNAAEKTT

Tell_MTB LINSCKSLNLPIKERAAFQPVQNPSSPSSSSSPAAAAPGTATMGKASSFENTPNT-EKMT

**.:*** *** :. . : :*.**:****:*** *. *.* ******: ** *

Tthe_MTB3 PTAVTLASYRQQETSGTGSQNLIEGRPRRKKPSKLSLLLKRDSGQKSGSSLSSRQQETPS

Tell_MTB PTAVTLSSYRQQETPGTNYQSQFEGKARKKNSSKLSILLKRDPSQKSGNSLSSRNQETPS

******:******* ** *. :**: *:*: ****:***** .****.*****:*****

Tthe_MTB3 PNLPSYSPNLYPSQAYI

Tell_MTB PNLPTYSPNLYPSQAYI

****:************
