# Supplementary material for: Selecting One of Several Mating Types through Gene Segment Joining and Deletion in Tetrahymena thermophila
Source: PLoS Biol. 2013 Mar 26;11(3):e1001518. doi: 10.1371/journal.pbio.1001518 (PMC3608545; doi:10.1371/journal.pbio.1001518)
Supplement: Text S6 — Germline consensus sequences for the transmembrane exons of MTA and MTB . Polymorphic sites are indicated by red font. (DOC) [file pbio.1001518.s017.doc]

**Text S6. Germline consensus sequences for the transmembrane exons of *MTA* and *MTB*.**

>*MTA*-TM Germline Consensus

TCAAAGCTCTCCCTCGCTTTCTTAGATTTGATTTAAGGTGGAGATTAGCTTGTCTACATTTCTGATGTTAGTGAGACAGAGGATTGTCATTACAATATCAAAAATAATAAGATCGTAAATTTGCACGTAGCTGGAGAGTACTTCAGGATACTGCAGGATCAGAAAGGACACAAGTGAGCATAAACCTGAAACGTTGCTCTTGATTTAAGACGAAAGCACTTTTCTAAAGTTTGTCAGCACAATGGTAGCTCTTTCGACGTCTTCTCCATATGGATATCTAAACCACCCTTCAACTGAAAAGATATTAGTTAGTGTCAAGCTCACAGACTTAGGTATGAGGCAGAAAGCTAATCTTGCAGGTACTATTAGAAAAGAAAGACAGCCTTCCCTCTTTGAAGAGGGCAGAGGATTCAAAAGACAGCTAGTCTTTGGCGTGTTCTCCGAGTTTGCAAGGATAGAACCTAACAAATATGCATCAAAAATCCACAATAGAGTGCTCAATGCACAAGCTCCGAGCAAGGTTTACAAAACAGGACTCTCAAAATCCCCTCCTGATACTGAAAAACCCCACAAAAGAGTGTAAGGCATTTGAATAGCCTCAGCAACACTTAACAAAAAAAGCATAAGACAATCTATTCGCTAACCACGCAATTCCTTTTCTCTTTCTCTCACTCCTTTATGCTCTTTCCCACTCGCATTCCTTCCAGCGCTACTGCTACCACTGCTGCTGCTTCTACTGCCGCTGCTGCTCCTCCTCATGCCGCTGCTTCTCCCATTCGAATGACTCTTCCCTTTCAATTTATTGTAGGTGTATCTGAGAACGCTCTAACTCACACTCACTGCCAATGCTGCCAACACTATCCATATAAAGATTGCTCCTCCTCCTTTTAAAGCGCCTACAGTATCTTGCAACTGGCCCATAACCTTACTGCCAGTATTGCTGCTGCTTTCGCCGCCTTCCCTTTAATCGGCCTTAGCACTTGAACTTTCTTCAGTCATTCTAGTCAAGGATTGCGCTTTGTCTGATTTTTGTGCATCATCTGGATGAGTAGTGTCTATATTATCTGCAAGATTTGTAGATAAAGAGTAATCTAATAATCTTGATCTTGCTGTCTGAAGCAAGTGACTATCGACGCACCCGTTGAACTCAGGCACCAGCGTATATCCTTGATTGCAGCTTGAGCATTAAGTGAGATTTGAAGGCGAACATGACAAGCATTAAGGGTCTTTTAGCTAGCAAGCTAAACAAGTACCATTATAGGCTACATATTAAGGGCTGCATGTAGGAAGACAAGCAAGGACGGCGTGATGATTGTACTGCGACTTGAGATATCCAGGAGCACAAGCTGTACAAGCAGCGTAATTTGATGCGCAAGCTTAGCAATTGGGTGAGCAAGTATATTTGGAAGTGATTGGGAGACTAACTGATTATATAACCCAAACATTTTTGAAACTATTTCCTTATTGTGTACTCCATATCAATTAAAAATTATATGTCTAGGCTACATTTTATTCATTTTATTCAACAATACCAGAGGGATTTCTCAAACCACTTATTCTAAGTTA

>*MTB*-TM Germline Consensus

AATATCACAATTCATAATCCTGAAATAAATAACTAAAATTCAACAGGAGGTGCTGCTTAATCAATAGCAATTAATTGGAATTTAACAAGCACTACAAGCTCTTAAACTCTTTTGTCGGGACACACTTCATTAACTACCAACACCTCCTAATGTCCTTAACCTCACTGCGCCACTTGCACATCTCCTCCATCCATTTGCATTCACTGCACCCAAGGCTATTATTTACTTCCAGATCAAAACTCATGCGTCCAGACTTGTCCACCTCCAACAGTCGCTCATCAACAAACAGCTACTTGCCAGCCATGCTTCCAGCACCAAGAGTGTCTTCAATGCCAGTCTCAAAACCCAGCTGCTTGCACCTCATGTTCTCCCACCTATTCCCTCAATTCCACTCTCCTCCCCTATTGCTACGTGCCTCTTCCTCCTTCTAGTAGCGCTTCTTCCTCCGTCACCAAGGATGTTGTCAATCGCACGCCTTCTAACTCAACCTTTTCAGGAGCACTCAATCGACCTGAGCCAGGACAGCCCTCACAAAAGTAGCAGTAGCAGTAGTAGTAGTAGCAGCAGGAGCAGCAGCAACAACAACAACAACAGCAGGAGGCCCAAGCTAGCGATCAGCGAGGCTTTGCTCATTTCTTAGCTCAAACCAAGTCTTATACGAAAGGCTTTATTTTGACACTGCTGATTCCCCTCTCAATACTCGGTGCTTGCCTCACTAGGCTAGTCACCTTTTGTCTGAAAAAGCGAGAAAAGAAGGTGCATCCACCACTGCCTAGTGAATCGAGGAGTGCACAGATTGCACAAAACTTGGATGAAAGGTAAGAAACACAAAAAGATGGAAATGGTGGCGATGAAGAACAGATGAGAGCGTCTTCGAGGGTAGACACAGCAAATATGTGTCCTCTGAACTCAAGAAGAGGCGAGTAGTTATAATTGGAAGGTGTGCAGTAATAAAGCGATGGTGGTGTTGGTGGTGGGGAAAGTGAAGGAAACGGGTATTTAGCGTTCTGCTGGATAGCAGTGATCCTTTTGTTGGGAAATGTGGGTGATTTAGTAGAGGTGCCTTATATTATCTTCTCACAGTAGAACTCGTTTTCTAACAAAAGCACAACAAATGTATTTGACTTGTAATTTTCAGAAGCAGACATGGGCTAGATTTGTTGTTTGAGCTACATCGCACTAAACGCAGTTTGCTACTTGATCTGCGTTGTAATGATGGTAAAGGCTATAATTTTTGAGACTGGAAGTGGTGAGCCTCTGTTTTGTATTTACGAAGTGAAGCTGAGTAGCAGTAGCAGTTTTTGTGGAGAAGAAATGAAGAAGGATAAAAGTGAAGCTGCTGCGTGCAAAGGGGATTCGAATGCTTAGAATGAGAATGGAGAGAAGCACAAAAAAGTGTTGAGTGGGCGAAAGCTATGGAAGCTAATAATAGATGTTTTCTTGAGATGTCTGGTAGTTGTTGGTGGAAAAGCTTTCTGCATGGTTTATTCAAACGTGGCAAATGTAAAGGGATGGTTAACATGCTAAGTAGATAAAAATTTGAGAGCATTTAGATTGTTCTATATGACTTTGTGCATCCATGCAATCTTCAATATGATATCAGCAGTATTCTTTACTCTCATGCTTACTCATTTCTCCTTTGCTTCTTGGACTGCAGTAGCACAAGATAGCCTTACAAGCTAAGGTAGCGATGGTGGAGTCGAATTTTCGTTCTTTGTTGACATCCTTGCATTTAAATTTTTAATGTCTCTGATTTGTTTTTTGAACTGCCTGCATATCTAACAGCTAATTAGTGCCTGCAAATCTCCTAATCTTCCTGGACCCTAAAATCCCGTTTCCCATCGACTCTAAAGCCCATCTACTCCTTCTTCTTCCGCATCACCTGCAGACGCAGTAGAAAGAGACGCTTGCAAAGTTTCATACTTTGAAAATACACCAAATGCTGCTGAAAAGACTACTCCAACTGCGGTAACGCTTGCCTCTTATCGCCAGCAAGAAACATCAGGAACTGGTTCTTAAAACCTCATAGAAGGTAGACCAAGGAGAAAAAAACCATCAAAGTTGAGTCTATTGCTAAAAAGAGATTCAGGATAAAAATCTGGTAGTTCACTTTCATCTAGACAGCAAGAAACACCTTCGCCAAATTTGCCTTCCTACTCTCCTAATCTCTACCCATCTCAAGCATACATTTGA
