# Supplementary material for: Selecting One of Several Mating Types through Gene Segment Joining and Deletion in Tetrahymena thermophila
Source: PLoS Biol. 2013 Mar 26;11(3):e1001518. doi: 10.1371/journal.pbio.1001518 (PMC3608545; doi:10.1371/journal.pbio.1001518)
Supplement: Text S7 — Collapsed alignments of germline and exconjugant somatic MTA -TM exon sequences. Exconjugant progeny cells had not yet undergone their first division (Figure S1 stage 3). See Figure 6 for an overview of these results. The 3′ end of each exon sequence is at the left of the alignment, to preserve genomic orientation relative to the MTB gene. Alignment numbering (written vertically) begins at the 5′ end of the germline MTA2-TM exon. To collapse the alignment, extended regions of identical bases in all exon sequences were replaced with a single dot. Deletions are represented as “-”. Bases shown are those that deviate from the germline consensus sequence (Text S5). Bases in yellow are unique to a germline mating type tm segment. The switch from dots to blank spaces indicates the end of a tm segment. Germ, germline sequence. First row below numbering: consensus sequence of germline TM exons, followed by the germline TM/tm exon sequences in order as found at the germline mat locus. Rows beginning with MTA: somatic TM exon sequences grouped by mating type. x followed by number, number of sequenced inserts having that sequence. *, location of a base not present in the germline; these changes could be due to either PCR errors or replication repair errors. *MTA2, 1065 C>A×1; *MTA3, 421 G>A×1. Somatic TM exon sequences are from progeny of parent strains SB210 and SB1969. (DOC) [file pbio.1001518.s018.doc]

**Text S7. Collapsed alignments of germline and exconjugant somatic *MTA*-TM exon sequences.**

**1000’s 1 1 1 1 1 1 1 11 1 1 1 1 1**

**100’s 6 6 6 6 6 6 77 7 7 777 7 7 7 77 7 7 7 8 8 8 8 8 8 9 0 0 1 2 2 3 4 55 5 5 5 5 5**

**10’s 6 7 8 8 9 9 00 1 6 677 7 7 7 88 8 9 9 0 2 3 3 5 6 8 1 7 1 3 6 0 3 11 2 3 3 4 5**

**1’s 8 0 2 5 0 5 78 8 7 901 3 5 7 23 5 1 6 6 3 2 7 1 9 6 0 3 4 8 1 3 7 01 0 1 7 9 3**

**Germ consensus ...C.C.C.T.C.G.GC.A....C.TCT.C.A.T.TG.C.C.C.T.G.C.T.G.A.C.G.T.C.C.G.G.C.TT.T..T.G.A.C.**

**Germ *MTA*2-TM .........................G..............................................G--21--.C.G...**

**Germ *MTA*5-tm .T.....A...A....T...........................A...................**

**Germ *MTA*6-tm ...T.A.T.A.A.A.AA.----54----........................A...A.A...........G...............**

**Germ *MTA*4-tm ........................**

**Germ *MTA*7-tm .....T....T...AA.T.A...A.T.T.A...T...A.....T...T.G...............**

**Germ *MTA*3-tm ...................A.................A.T..........-9-........G.**

***MTA*2-TM x10* .........................G..............................................G--21--.C.G...**

***MTA*3-TM x7* .........................G...............................................-9-........G.**

***MTA*3-TM x4 .........................G..................................A.T..........-9-........G.**

***MTA*4-TM x9 .........................G............................................................**

***MTA*4-TM x1 .........................G........................................A...................**

***MTA*5-TM x6 .........................G............................................................**

***MTA*5-TM x5 .........................G........................................A...................**

***MTA*6-TM x8 .........................G............................................G...............**

***MTA*6-TM x2 .........................G............................................................**

***MTA*7-TM x8 .........................G............................................................**

***MTA*7-TM x1 .........................G............................................G...............**

***MTA*7-TM x1 .........................G......................................T...T.G...............**
