# Supplementary material for: Selecting One of Several Mating Types through Gene Segment Joining and Deletion in Tetrahymena thermophila
Source: PLoS Biol. 2013 Mar 26;11(3):e1001518. doi: 10.1371/journal.pbio.1001518 (PMC3608545; doi:10.1371/journal.pbio.1001518)
Supplement: Text S8 — Collapsed alignments of germline and exconjugant somatic MTB -TM exon sequences. See Figure 6 for a diagrammatic overview of these results. Exon sequences were aligned as in Text S7. Symbols are as in Text S7. *MTB2, 134 C>T×1; *MTB3, 1365 T>C×1; *MTB5, 1368 G>T×1; *MTB6, 583 del ACA ×1; *MTB6, 977 G>T×1. (DOC) [file pbio.1001518.s019.doc]

**Text S8. Collapsed alignments of germline and exconjugant somatic *MTB* -TM exon sequences.**

**1000’s 1 1 1 1 1 1 1 1 1**

**100’s 1 2 2 3 3 3 3 4 4 5 5 5 5 5 5 5 5555 5 5555 5 55 555 6 6 6 6 6 6 6 6 7 8 2 2 2 2 3 3 3 3 4**

**10’s 7 9 0 9 4 6 6 9 1 9 0 0 1 2 3 3 4 4445 5 6666 6 77 888 0 0 1 2 4 5 6 6 2 6 0 0 6 7 1 2 5 7 1**

**1’s 4 1 9 7 5 0 5 0 2 4 2 9 2 3 0 2 3 7890 9 1234 7 67 678 2 7 2 4 8 1 0 5 9 4 7 9 5 2 7 0 4 4 7**

**Germ consensus .A.C.C.C.C.C.G.C.G.C.C.G.C.C.C.C.G.CAGT.T.G---.G.GC.---.C.G.C.C.C.G.G.G.C.G.G.T.G.C.G.G.G.G.T.**

**Germ *MTB*2-tm ...A...........T.....A.............T....C..CAG.A.AA.---.........T...........................C.**

**Germ *MTB*5-tm ...A.........T**

**Germ *MTB*6-tm ...A...T.........T.A...A.T.A.T.T.T.TCAC.G.------42-------.T.T.T.T.T.A.A.**

**Germ *MTB*4-tm .....T.....................................---......---.................T.....C.....A.....A...**

**Germ *MTB*7-tm .G.......T.................................---......GAA...................A.....A.T...A.T.....**

**Germ *MTB*3-TM ...........A...............................TAG......---.....................A.................**

***MTB*2-TM x9 ...........A...............................TAG......---.....................A.................**

***MTB*2-TM x8 ...A.....T.................................TAG......GAA.....................A.................**

***MTB*2-TM x5 .........T.................................TAG......---.............A.......A.................**

***MTB*2-TM x4 ...A.......A...............................TAG......---.....................A.................**

***MTB*2-TM x1 ...............T...........................TAG......GAA.......................C...............**

***MTB*2-TM x1 ...........A...............................TAG......GAA.....................A.................**

***MTB*2-TM x1 ...........A...............................TAG......---.............A.........................**

***MTB*2-TM x1* ...A.......A...............................TAG......---.............A.......A.................**

***MTB*2-TM x1 .........T.................................TAG......---.....................A.................**

***MTB*3-TM x23* ...........A...............................TAG......---.....................A.................**

***MTB*4-TM x7 ...........A...............................TAG......---.....................A.................**

***MTB*4-TM x5 .....T.....................................---......---.................T...A.................**

***MTB*4-TM x5 .....T.....................................TAG......---.....................A.................**

***MTB*4-TM x4 .....T.....................................---......---.....................A.................**

***MTB*4-TM x2 .....T.....A...............................TAG......---.....................A.................**

***MTB*4-TM x1 ...........................................TAG......---.................T.....C...............**

***MTB*4-TM x1 ...........A...............................---......---.................T...A.................**

***MTB*4-TM x1 .....T.....................................TAG......---.......................C.....A.....A...**

***MTB*4-TM x1 .....T.....................................---......---.................T.....C.....A.....A...**

***MTB*4-TM x1 .....T.....................................---......---.......................C...............**

**1000’s 1 1 1 1 1 1 1 1 1**

**100’s 1 2 2 3 3 3 3 4 4 5 5 5 5 5 5 5 5555 5 5555 5 55 555 6 6 6 6 6 6 6 6 7 8 2 2 2 2 3 3 3 3 4**

**10’s 7 9 0 9 4 6 6 9 1 9 0 0 1 2 3 3 4 4445 5 6666 6 77 888 0 0 1 2 4 5 6 6 2 6 0 0 6 7 1 2 5 7 1**

**1’s 4 1 9 7 5 0 5 0 2 4 2 9 2 3 0 2 3 7890 9 1234 7 67 678 2 7 2 4 8 1 0 5 9 4 7 9 5 2 7 0 4 4 7**

**Germ consensus .A.C.C.C.C.C.G.C.G.C.C.G.C.C.C.C.G.CAGT.T.G---.G.GC.---.C.G.C.C.C.G.G.G.C.G.G.T.G.C.G.G.G.G.T.**

**Germ *MTB*2-tm ...A...........T.....A.............T....C..CAG.A.AA.---.........T...........................C.**

**Germ *MTB*5-tm ...A.........T**

**Germ *MTB*6-tm ...A...T.........T.A...A.T.A.T.T.T.TCAC.G.------42-------.T.T.T.T.T.A.A.**

**Germ *MTB*4-tm .....T.....................................---......---.................T.....C.....A.....A...**

**Germ *MTB*7-tm .G.......T.................................---......GAA...................A.....A.T...A.T.....**

**Germ *MTB*3-TM ...........A...............................TAG......---.....................A.................**

***MTB*5-TM x14 ...........A...............................TAG......---.....................A.................**

***MTB*5-TM x5 ...A.......A...............................TAG......---.....................A.................**

***MTB*5-TM x2 .....T.....................................TAG......---.....................A.................**

***MTB*5-TM x2 ...A...........T.....A.....................CAG.A.AA.---.........T...........A.................**

***MTB*5-TM x1 ...........A...............................---......---.....................A.................**

***MTB*5-TM x1* ...........A...............................TAG......---.....................A.................**

***MTB*5-TM x1 .....T.....A...............................TAG......---.....................A.................**

***MTB*5-TM x1 ...A...........T.....A.....................CAG.A.AA.---.........T.............................**

***MTB*5-TM x1 ...A.......................................TAG......---.....................A.................**

***MTB*5-TM x1 ...A...........T...........................TAG......---.....................A.................**

***MTB*5-TM x1 ...A...........T.....A.....................CAG.A.AA.---.....................A.................**

***MTB*6-TM x16* ...........A...............................TAG......---.....................A.................**

***MTB*6-TM x5 ...A.......A...............................TAG......---.....................A.................**

***MTB*6-TM x3 ...........A...............................TAG......---.........T...........A.................**

***MTB*6-TM x2 ...A...........T.....A.............T....C..CAG.A.AA.---.........T...........A.................**

***MTB*6-TM x1 ...............T.....A.............T....C..CAG.A.AA.---.........T...........A.................**

***MTB*6-TM x1 ...A...........T.....A.............T....C..CAG.A.AA.---.........T.............................**

***MTB*6-TM x1 ...........A...............................---......---.....................A.................**

***MTB*6-TM x1* ...A...........T.....A.....................CAG......---.....................A.................**

***MTB*7-TM x10 .G.......T.................................---......GAA.....................A.................**

***MTB*7-TM x6 .G.......T.................................---......GAA...................A.A.................**

***MTB*7-TM x6 .G.......T.................................TAG......---.....................A.................**

***MTB*7-TM x4 .G.........A...............................---......GAA.....................A.................**

***MTB*7-TM x3 .G.........A...............................TAG......---.....................A.................**

***MTB*7-TM x1 .G.......T.................................---......GAA...................A...C...............**
