# Supplementary material for: Selecting One of Several Mating Types through Gene Segment Joining and Deletion in Tetrahymena thermophila
Source: PLoS Biol. 2013 Mar 26;11(3):e1001518. doi: 10.1371/journal.pbio.1001518 (PMC3608545; doi:10.1371/journal.pbio.1001518)
Supplement: Text S9 — PCR template switching could only have affected the apparent diversity of TM exon haplotypes in 24-h exconjugants. (DOC) [file pbio.1001518.s020.doc]

**Text S9. PCR template switching could only have affected the apparent diversity of TM exon haplotypes in 24-hr exconjugants.**

PCR template switching occurs in the late stages of PCR when product concentrations are high. When a given primer pair results in the exponential PCR amplification of different DNA segments, which share homology but have polymorphic differences, artifactual new combinations of the polymorphic nucleotides can be found among the products due to PCR template switching [29]. PCR amplification was used extensively in this work, and homologous, polymorphic transmembrane exon segments are present in the six germline mating type gene pairs and in the developing somatic nuclei, so it is important to assess the impact of PCR template switching on our results. The two most relevant cases involve amplification of the TM exons of: 1) 24-hr (zero-fission) exconjugants and 2) mature, assorted 120-fission cell lines. We provide several strong, independent lines of evidence that if any PCR template switching occurred, it did not significantly affect the most important conclusions of this work.

A) 24-hr exconjugant cultures.

Several independent lines of evidence suggest that the contribution of *linearly* amplified PCR products to template switching artifacts is minimal or nonexistent.

1) None of the 10 *MTA2* or the 23 *MTB3* sequenced TM exons showed any evidence of recombination, from either PCR template switching or of biological origin (Supplementary Text S3, S4 and S5).

2) *MTA* haplotypes of the remaining five mating types, 50/51 (98%) showed just the single, simple recombination event required to join the mt-specific segment to the full length TM exon of the germline *MTA2* gene. Even for *MTB*, which shows more recombination, 74% of the observed haplotypes can be explained by the single, simple recombination event required to join the mt-specific region to the *MTB3* TM exon.

3) Preliminary time course experiments during somatic nucleus differentiation show no PCR products up to the time when joining first occurs (our unpublished observations, referred to in the discussion section, main text).

4) Dozens of unique polymorphic nucleotides in the twelve germline transmembrane exon segments failed to appear in the sequenced PCR products from 61 somatic *MTA* and 172 somatic *MTB* TM exons (Supplementary Text S8 and S9 and Table S7).

On the other hand, cohorts of TM exons already joined to the same mt-specific segment were *exponentially* amplified in one another’s presence and thus are subject to PCR template switching. We cannot estimate what fraction of the observed recombinant haplotypes in 24-hr exconjugants are due to PCR template switching because additional secondary recombination events of biological origin are observed in mature, assorted cell lines (see below). Nevertheless, PCR template switching cannot increase overall nucleotide diversity of the products; it can only spuriously reshuffle it. Therefore, the only possible effect of any PCR template switching would have been to exaggerate the diversity of sequenced haplotypes and of join locations and the number of apparent gene conversions.

B) Mature, 120-fission clones.

Somatic *MTB* genes were sequenced from mature cell lines representing each mating type. Complex recombination patterns were observed in the *MTB* TM exons of each strain, including SB210, which was sequenced as part of the whole-genome macronuclear genome sequencing project [15]. Surprised by the extent of TM exon recombination, we sequenced the TM exons of 14 additional mature cell lines (at least 3 cell lines of each mating types in total; data in Text S8 and S9, summary in Table S7).

No PCR was used in the sequencing of SB210, so PCR template switching is excluded as the source of the TM exon recombination observed in this cell line. For the other strains both germline (linearly amplified) and somatic (exponentially amplified) DNA were present in the genomic DNA we used for PCR amplification, so the possibility exists that PCR template switching could be contributing to the observed recombination. To determine if this was the case, RNA isolated from starved cells of each strain was DNase treated and the *MTB* TM exons were amplified by RT-PCR. Cells of each strain express a single somatic mating type; the germline is not expressed, so there is no possibility of artifacts due to PCR template switching. In every case, the sequences previously obtained by sequencing genomic PCR products agreed exactly with those obtained by sequencing RT-PCR products. Thus PCR template switching was not responsible for any of the complex recombinant patterns observed in these 120-fission cell lines.

C. Summary.

We find no evidence that template switching caused by *linearly* amplified PCR products significantly affected the diversity of haplotypes observed in either 24-hr (zero fission) cultures or mature (120 fission) cell lines. PCR template switching is problematic for the 24-hr exconjugants, but even there the worst consequence would have been to exaggerate the diversity of sequenced haplotypes and joint locations and the number of apparent gene conversions.
