# Supplementary material for: Selecting One of Several Mating Types through Gene Segment Joining and Deletion in Tetrahymena thermophila
Source: PLoS Biol. 2013 Mar 26;11(3):e1001518. doi: 10.1371/journal.pbio.1001518 (PMC3608545; doi:10.1371/journal.pbio.1001518)
Supplement: Text S10 — Collapsed alignments of the somatic MTA -TM exon sequences from mature strains. SB210 and SB1969 are the parents of the SB4200's F1 cell lines. Soma, somatic nucleus. Orientation and symbols are as in Text S7. (DOC) [file pbio.1001518.s021.doc]

**Text S10. Collapsed alignments of the somatic *MTA*-TM exon sequences from mature strains.**

**1000’s 1 1 1 1 1 1 1 11 1 1 1 1**

**100’s 77 77 7 7 7 77 7 7 7 8 8 8 8 8 8 9 0 0 1 2 2 3 4 55 5 5 5 5**

**10’s 66 67 7 7 7 88 8 9 9 0 2 3 3 6 7 8 1 7 1 3 6 0 3 11 3 3 4 5**

**1’s 67 90 3 5 7 23 5 1 6 6 3 2 7 1 9 6 0 3 4 8 1 3 7 89 1 7 9 3**

**Germ consensus ..GC.TC.C.A.T.TG.C.C.C.T.G.C.T.G.A.C.G.T.C.C.G.G.C.TT..A.G.A.C.**

**Germ *MTA*2-TM ........G............................................-21-.G.C.G...**

**Germ *MTA*5-tm ...T....A...A....T...........................A.................**

**Germ *MTA*6-tm .....A..G.........................A...A.A...........G.............**

**Germ *MTA*4-tm .....................**

**Germ *MTA*7-tm .......T...T...AA.T.A...A.T.T.A...T...A.....T...T.G.............**

**Germ *MTA*3-tm SB210 .....................A.................A.T.........-9..........**

**Germ *MTA*3-tm SB1969 .....................A.................A.T.........-9..........**

**Soma *MTA*2-TM SB1969 ........G............................................-21-.G.C.G...**

**Soma *MTA*2-TM SB4208 ........G............................................-21-.G.C.G...**

**Soma *MTA*2-TM SB4209 ........G............................................-21-.G.C.G...**

**Soma *MTA*2-TM SB4210 ........G............................................-21-.G.C.G...**

**Soma *MTA*3-TM SB4211 ........G.............................................-9..........**

**Soma *MTA*3-TM SB4212 ........G.............................................-9..........**

**Soma *MTA*3-TM SB4213 ........G.............................................-9..........**

**Soma *MTA*4-TM SB4214 ........G.........................................................**

**Soma *MTA*4-TM SB4215 ........G.........................................................**

**Soma *MTA*4-TM SB4216 ........G.........................................................**

**Soma *MTA*5-TM SB4217 ........G.........................................................**

**Soma *MTA*5-TM SB4218 ........G.........................................................**

**Soma *MTA*5-TM SB4219 ........G...........................................G.............**

**Soma *MTA*6-TM SB210 ........G...........................................G.............**

**Soma *MTA*6-TM SB4220 ........G.........................................................**

**Soma *MTA*6-TM SB4221 ........G...........................................G.............**

**Soma *MTA*6-TM SB4222 ........G.........................................................**

**Soma *MTA*7-TM SB4223 ........G...........................................G.............**

**Soma *MTA*7-TM SB4224 ........G...........................................G.............**

**Soma *MTA*7-TM SB4225 ........G...........................................G.............**
