# Supplementary material for: Selecting One of Several Mating Types through Gene Segment Joining and Deletion in Tetrahymena thermophila
Source: PLoS Biol. 2013 Mar 26;11(3):e1001518. doi: 10.1371/journal.pbio.1001518 (PMC3608545; doi:10.1371/journal.pbio.1001518)
Supplement: Text S11 — Collapsed alignments of the somatic MTB -TM exon sequences from mature strains. Orientation is as in Text S8 and symbols are as in Text S7. (DOC) [file pbio.1001518.s022.doc]

**Text S11. Collapsed alignments of the somatic *MTB*-TM exon sequences from mature strains.**

**1000’s 1 1 1 1 1 1 1 1 1**

**100’s 1 2 2 3 3 3 3 4 4 5 5 5 5 5 5 5 5555 5 5555 5 55 555 6 6 6 6 6 6 6 6 7 8 2 2 2 2 3 3 3 3 4**

**10’s 7 9 0 9 4 6 6 9 1 9 0 0 1 2 3 3 4 4445 5 6666 6 77 888 0 0 1 2 4 5 6 6 2 6 0 0 6 7 1 2 5 7 1**

**1’s 4 1 9 7 5 0 5 0 2 4 2 9 2 3 0 2 3 7890 9 1234 7 67 678 2 7 2 4 8 1 0 5 9 4 7 9 5 2 7 0 4 4 7**

**Germ consensus .A.C.C.C.C.C.G.C.G.C.C.G.C.C.C.C.G.CAGT.T.G---.G.GC.---.C.G.C.C.C.G.G.G.C.G.G.T.G.C.G.G.G.G.T.**

**Germ *MTB*2-tm ...A...........T.....A.............T....C..CAG.A.AA.---.........T...........................C.**

**Germ *MTB*5-tm ...A.........T**

**Germ *MTB*6-tm ...A...T.........T.A...A.T.A.T.T.T.TCAC.G.------42-------.T.T.T.T.T.A.A.**

**Germ *MTB*4-tm .....T.....................................---......---.................T.....C.....A.....A...**

**Germ *MTB*7-tm .G.......T.................................---......GAA...................A.....A.T...A.T.....**

**Germ *MTB*3-TM SB1969 ...........A...............................TAG..............................A.................**

**Germ *MTB*3-TM SB210 ...........A...............................TAG..............................A.................**

**Soma *MTB*2-TM SB4208 ...A...........T.....A.............T....C..CAG.A.AA.---.........T...........A.................**

**Soma *MTB*2-TM SB4209 .....T.....A...............................TAG..............................A.................**

**Soma *MTB*2-TM SB4210 ...........A...............................TAG..............................A.................**

**Soma *MTB*2-TM SB1969 ...........A...............................TAG..............................A.................**

**Soma *MTB*3-TM SB4213 ...........A...............................TAG..............................A.................**

**Soma *MTB*3-TM SB4211 ...........A...............................TAG..............................................C.**

**Soma *MTB*3-TM SB4212 ...........................................TAG......GAA.....................A.................**

**Soma *MTB*4-TM SB4215 ...........................................TAG......GAA.....................A.................**

**Soma *MTB*4-TM SB4214 .G.........................................TAG......GAA.......................C...............**

**Soma *MTB*4-TM SB4216 .G.........................................TAG......GAA.......................C...............**

**Soma *MTB*5-TM SB4217 ...........................................TAG..............................A.................**

**Soma *MTB*5-TM SB4218 .G.........................................TAG......GAA.......................................**

**Soma *MTB*5-TM SB4219 .G.......T.................................TAG......GAA.......................C...............**

**Soma *MTB*6-TM SB4220 .G.........A...............................TAG......GAA.....................A.................**

**Soma *MTB*6-TM SB4221 .G.........................................TAG......GAA.......................C...............**

**Soma *MTB*6-TM SB4222 .G.........................................TAG......GAA.....................A.................**

**Soma *MTB*6-TM SB210 ...........................................TAG......GAA.......................C...............**

**Soma *MTB*7-TM SB4223 .G.........................................TAG......GAA.....................A.................**

**Soma *MTB*7-TM SB4224 .G.........A...............................TAG......GAA.......................................**

**Soma *MTB*7-TM SB4225 .G.......T.................................TAG......GAA.....................A.................**
